# Supplementary figures and images for: Inhibition of Wnt signaling in primary human hepatocytes promotes Plasmodium falciparum liver stage development
Source: PLoS Pathog. 2025 Dec 22;21(12):e1013800. doi: 10.1371/journal.ppat.1013800 (PMC12795456; doi:10.1371/journal.ppat.1013800)

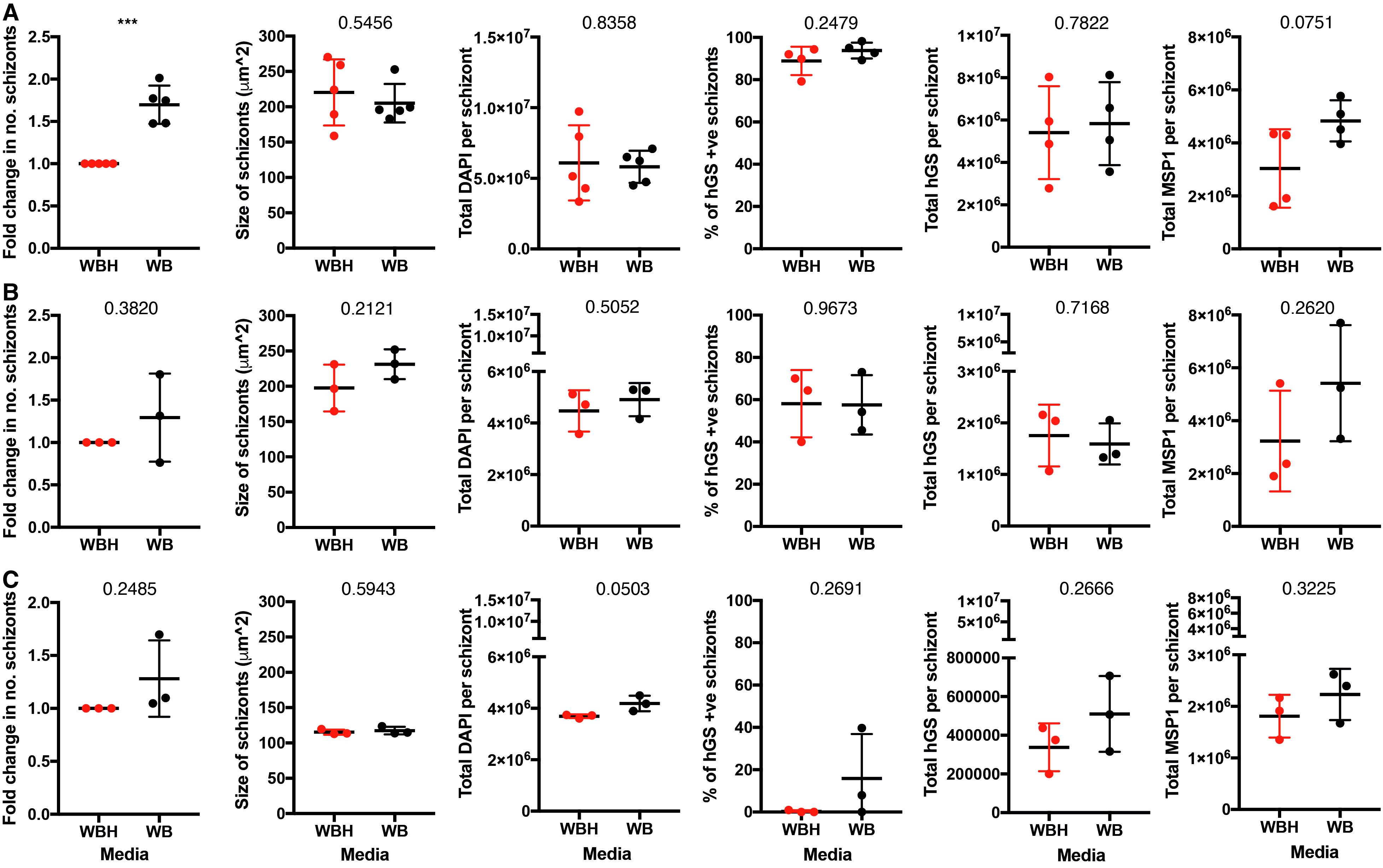

Supplement: S1 Fig — PHH monolayers were cultured in either William’s B (WB) or William’s B supplemented with 10% heat inactivated sera (WBH) for six days p.p. and infected with either NF175 (A) or NF135 (B) or NF54 (C). Data are shown for respectively schizont number, size, total DAPI content, percentage of human glutamine synthetase (hGS) positive schizonts, total hGS level per schizont and total merozoite surface protein 1 (MSP1) level per schizont. As for the number of schizonts, each dot represents an independent experiment showing the mean of two replicates. As for the size and total DAPI content graphs, each dot represents an independent experiment showing the mean of the median of at least 100 schizonts per replicate (2 replicates) were measured (except in NF54 condition where there is not enough schizonts present). For the percentage of hGS positive schizonts, each dot represents an independent experiment where at least 100 schizonts were examined from one replicate. For the total hGS level and total MSP1 level, each dot represents an independent experiment of the median from at least 100 schizonts (except in NF54 condition where there is not enough schizonts present). The error bars show the mean with the standard deviation. The p-values are generated by performing an unpaired t-test between WBH and WB and can be found in S1 Data (*** = 0.0001). (TIF) [file ppat.1013800.s001.tif]

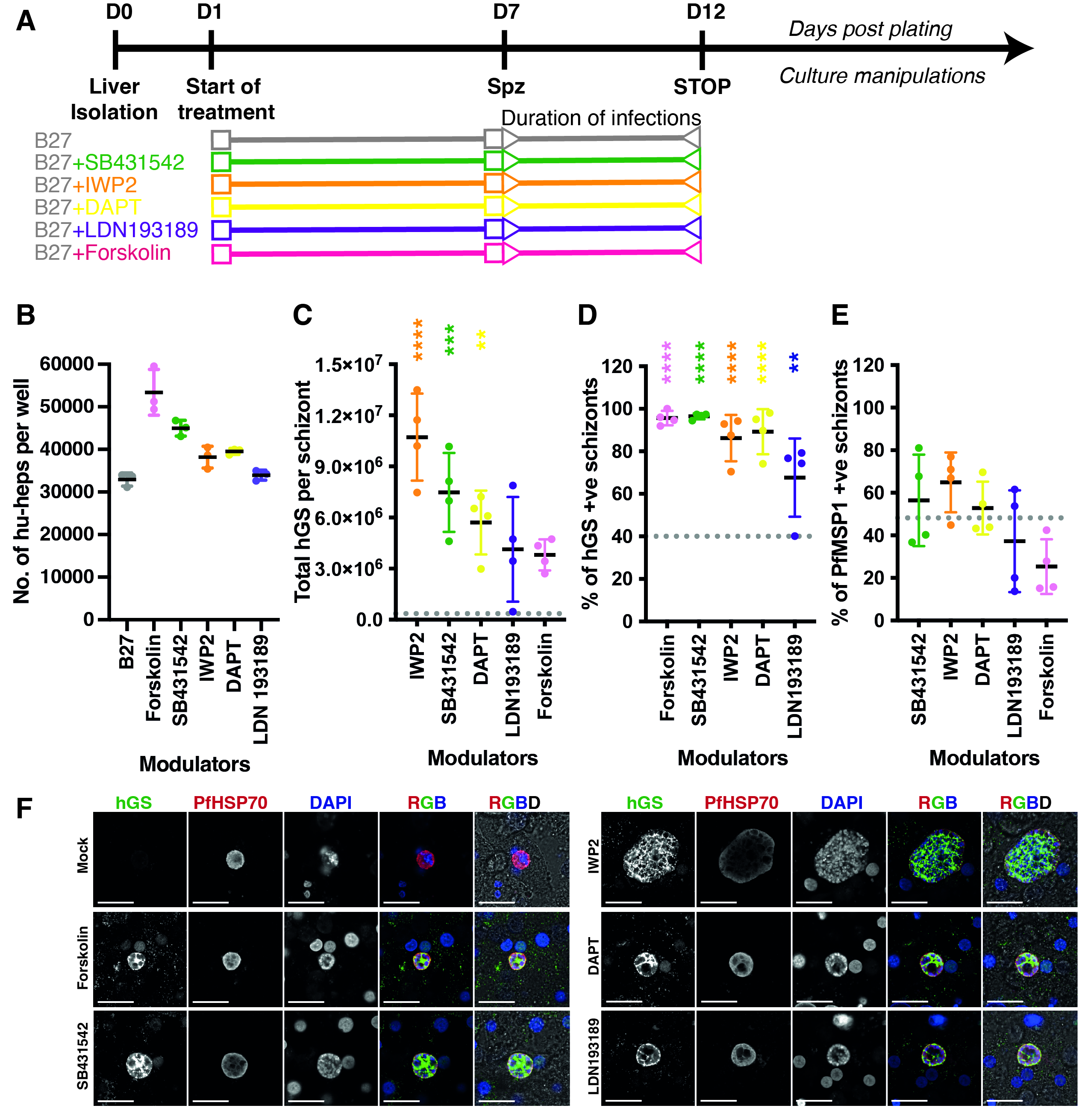

Supplement: S2 Fig — PHHs were treated for 6 days p.p with either B27 or B27 supplemented IWP2/SB431542/Forskolin/DAPT/LDN193189. The experiment was analysed at day 5 p.i. with NF175 B) Total number of hepatocytes per well with each dot representing the average three wells of one biological replicate. C)Total hGS per schizont with each dot representing the median of at least 100 schizonts. The percentage of hGS (D) and MSP1 (E) positive schizonts: each dot is an independent experiment where at least 100 schizont is measured. The grey dotted line (per graphs C-E) shows the median measurements of schizonts grown in B27 (from four independent experiments) as each host pathway inhibitor treatment was made in media containing B27. The mean and standard deviation is shown for each graph. The p-values from a Dunnett’s multiple comparisons test are displayed. F) Representative confocal images (from four independent biological experiments) showing schizonts grown in B27 alone and B27 supplemented with IWP2, SB431542, DAPT, LDN193189, Forskolin stained with hGS, HSP70 and DAPI. Scale bar is 25 microns. (TIF) [file ppat.1013800.s002.tif]

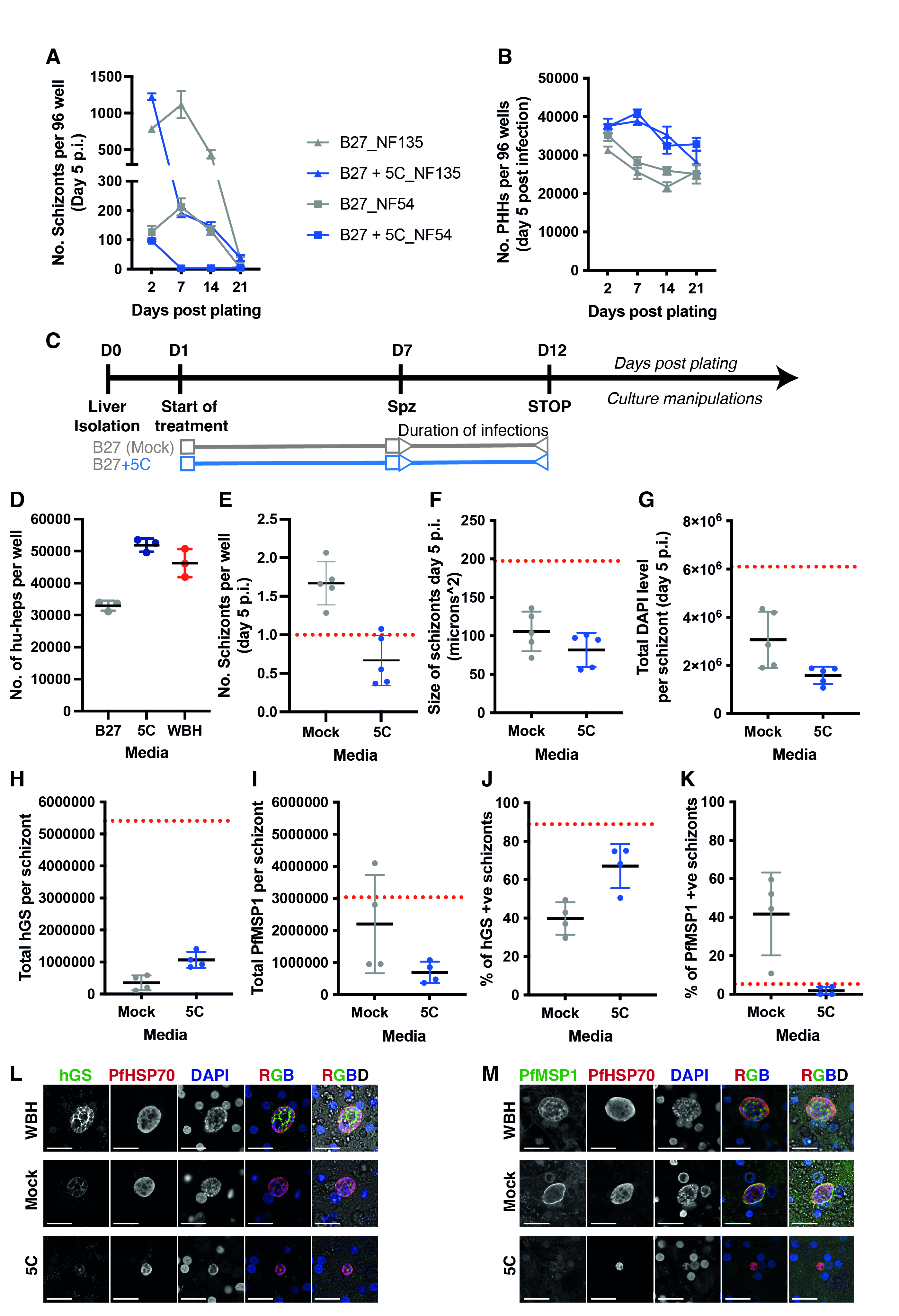

Supplement: S3 Fig — The number of schizonts (A) and hepatocytes (B) on day 5 p.i. for NF135 and NF54 (n = 1) at indicated time points p.p. when cultured in either B27 medium (grey) or B27 supplemented with 5C (blue). Each dot shows the mean of three replicates and the error bars show the standard deviation. C) Experimental setup of graphs D-L. PHHs were cultured for 6 days p.p. with either B27 or B27 with 5C and infected with NF175 parasites and analysed at day 5 p.i. D) Total number of hepatocytes per well with each dot representing the average three wells of one biological replicate. E)The number of schizonts per well was normalised to the number of schizonts counted in WBH (red dotted line). Each dot is the mean of an independent experiment with two replicates. For the schizont size (F) and total DAPI content per schizont (G), each dot represents the mean of an independent experiment of at least 100 schizonts. For the total hGS (H) and MSP1 (I) per schizont, each dot represents the median of an independent experiment of at least 100 schizont. For the percentage of hGS (J) and MSP1 (K) positive cells, each dot represents an independent experiment of at least 100 schizonts. D-K shows the mean with the standard deviation). Representative confocal images (from four independent experiments) showing schizonts grown in WBH or B27 alone or B27 supplemented with 5C on day 5 p.i. stained with hGS (L) or MSP1 (M), HSP70 and DAPI. Scale bar is 25 microns. (TIF) [file ppat.1013800.s003.tif]

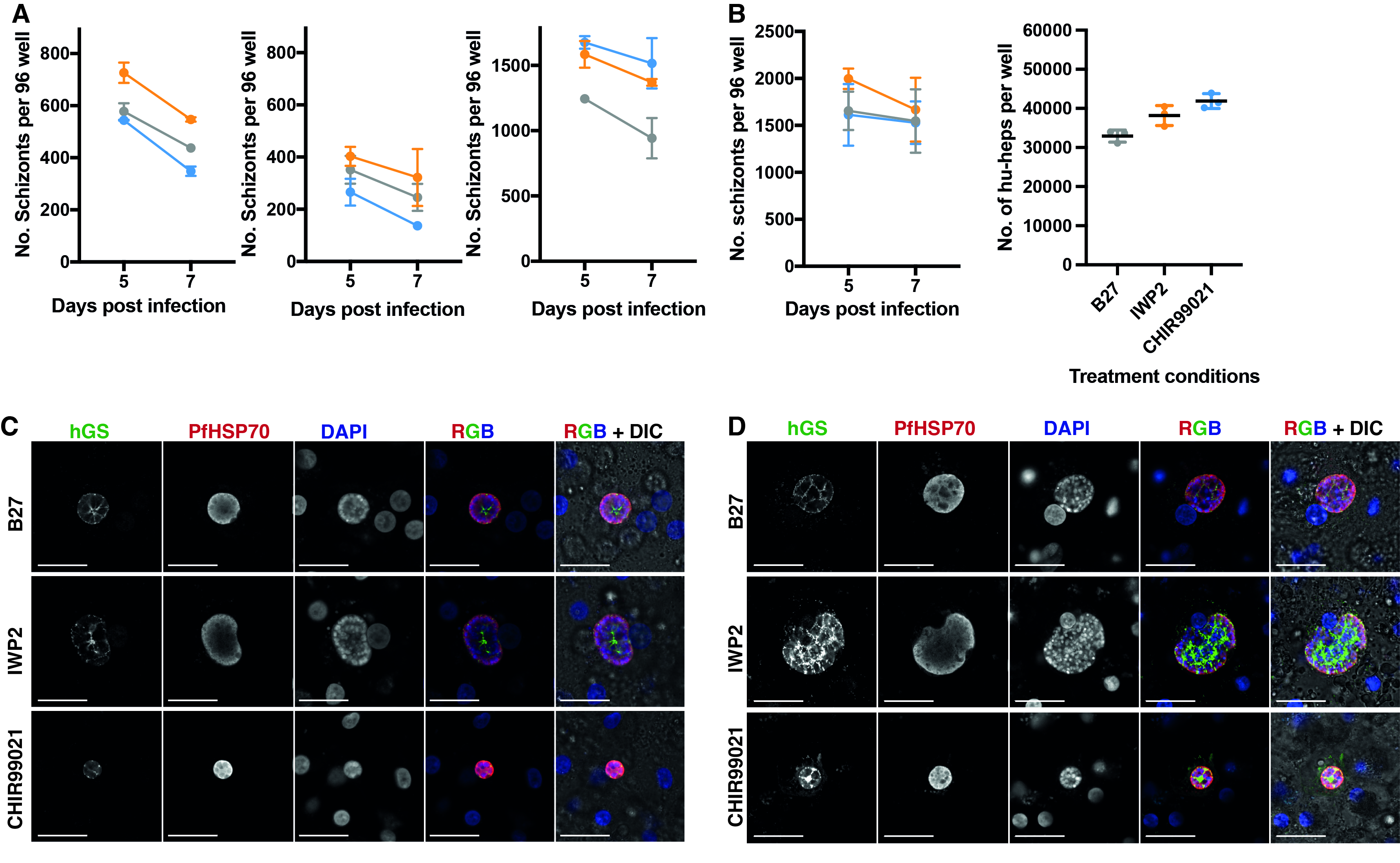

Supplement: S4 Fig — A) The number of schizonts in NF135 infected PHHs treated with B27 or B27 supplemented with IWP2/CHIR99021. Each graph shows an independent experiment, each with duplicates per condition. B) Number of schizonts (left) and number of hepatocytes (right) in NF175-infected PHHs treated with B27 or B27 supplemented with IWP2/CHIR99021. Each graph shows the mean and the standard deviation of three independent experiments: each experiments have duplicate wells. There was no statistic difference between conditions within the same day post infection. Representative confocal images (from three independent biological experiments) showing NF135 (C) and NF175 (D) schizonts B27 or B27 supplemented with IWP2/CHIR99021 on day 5 p.i. stained with hGS, HSP70 and DAPI. Scale bar is 25 microns. (TIF) [file ppat.1013800.s004.tif]

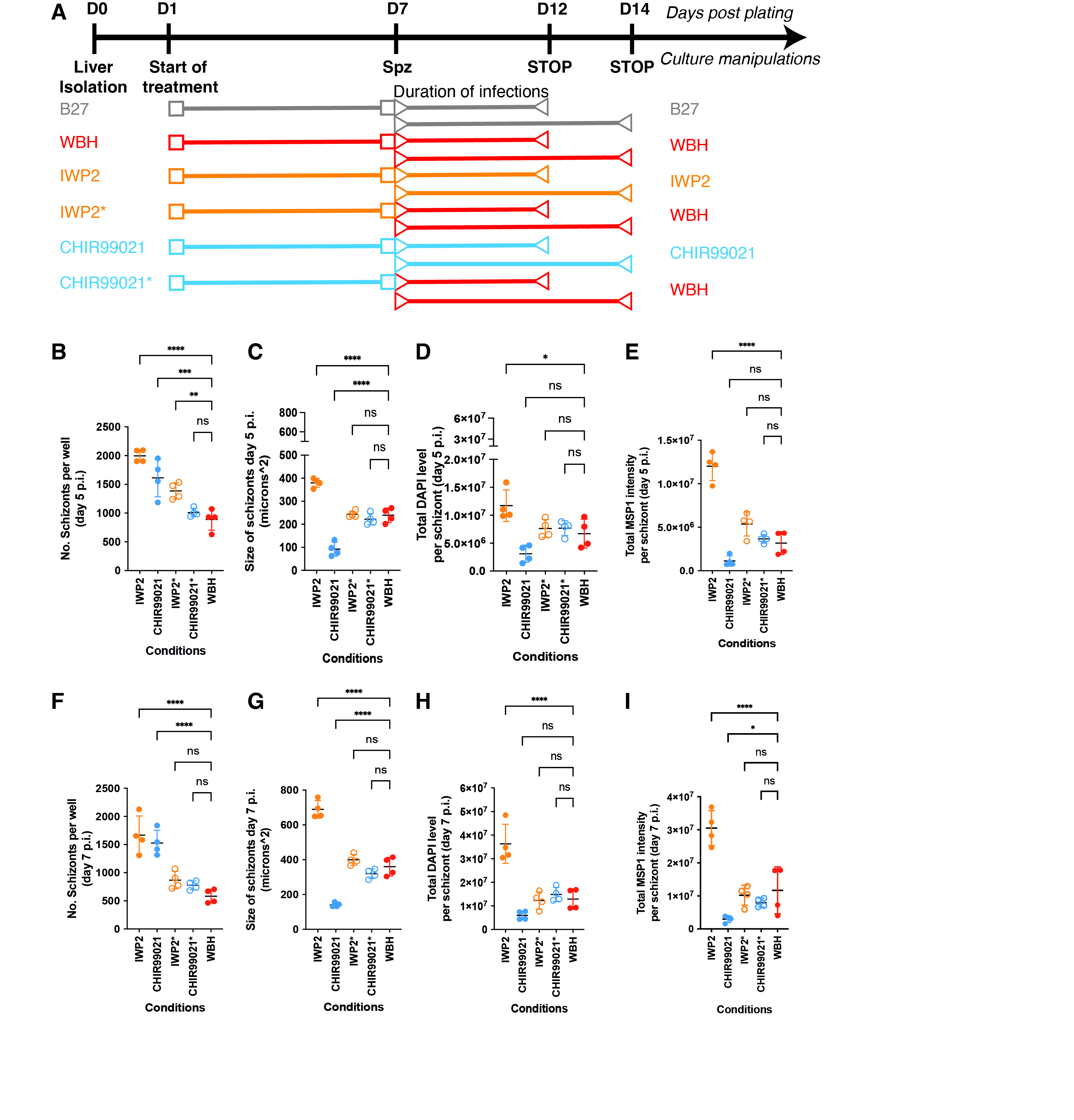

Supplement: S5 Fig — Hepatocytes are treated with B27 or B27 supplemented with IWP2/CHIR99021. After exposure to NF175, cultures were either kept in B27 or B27 supplemented with IWP2/CHIR99021 (closed circles) or returned to WBH (IWP2* and CHIR99021* - open circles). The number of schizonts (B), size of schizonts (C), total nuclear content per schizont (D) and total MSP1 per schizont (E) on day 5 post infection. The number of schizonts (F), size of schizonts (G), total nuclear content per schizont (H) and total MSP1 per schizont (I) on day 7 post infection. Each dot represents an independent biological donor where the median of at least 100 schizonts were measured. Dunnett’s multiple comparison test (to WBH) were performed for each graph, and the statistical significance is indicated: see supplemental information excel sheet for the full p values (S1 Data). (TIF) [file ppat.1013800.s005.tif]

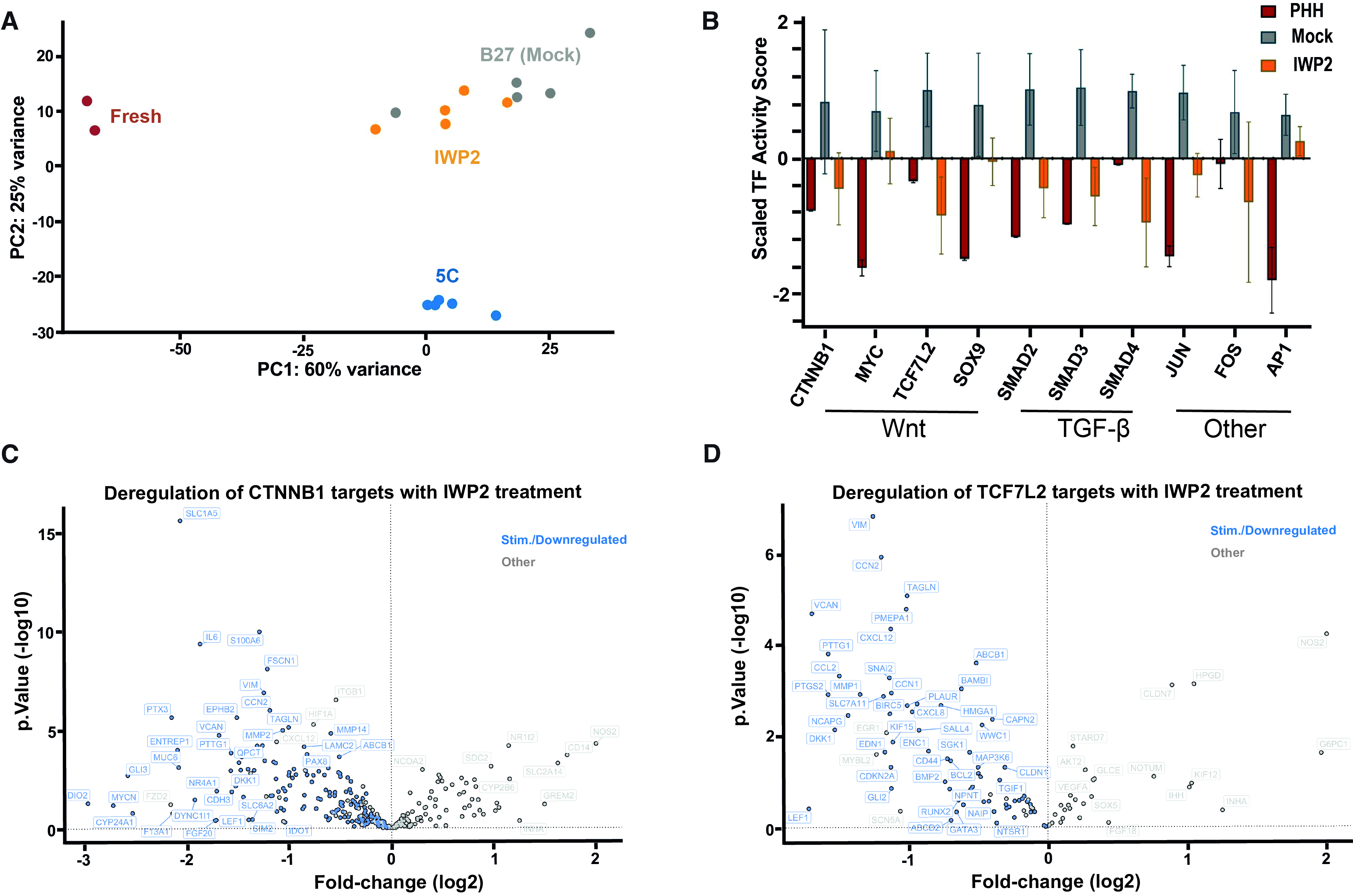

Supplement: S6 Fig — A) PCA plot of all replicates of freshly-isolated, “mock-cultured”, IWP2 or 5C-treated hepatocytes. B) Bar-plot depicting the scaled activity scores of key Wnt, TGFβ and non-canonical Wnt transcription factors across freshly-isolated hepatocytes, “mock-” and IWP2-treated hepatocytes. C-D) Volcano plots depicting a repressive effect of IWP2 treatment on the target genes underlying key Wnt signaling transcription factors CTNNB1 (β-catenin; C) and TCF7L2 (D). Genes that are positively modulated by these TFs and suppressed upon IWP2 treatment are highlighted in blue. (TIF) [file ppat.1013800.s006.tif]

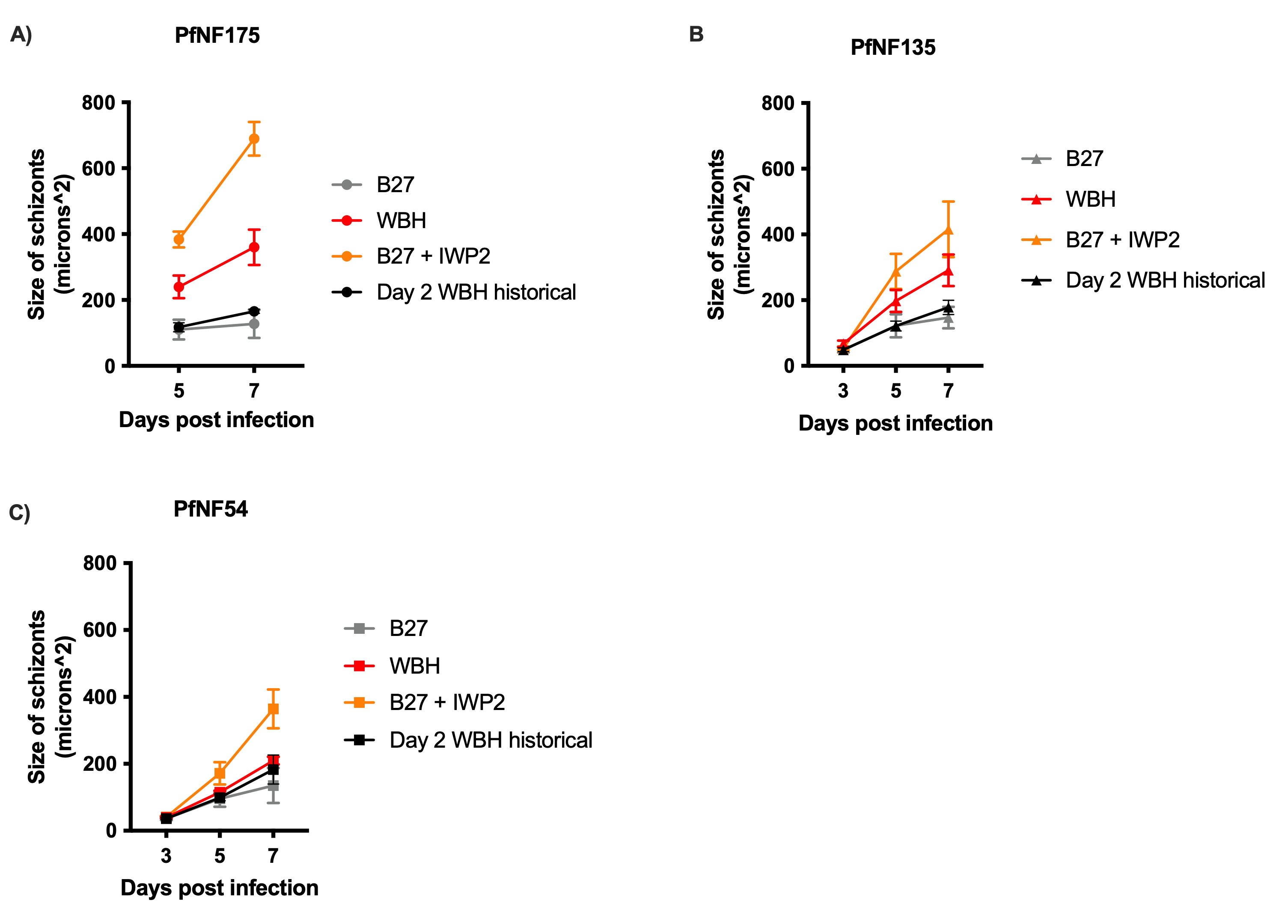

Supplement: S7 Fig — (TIFF) [file ppat.1013800.s007.tiff]

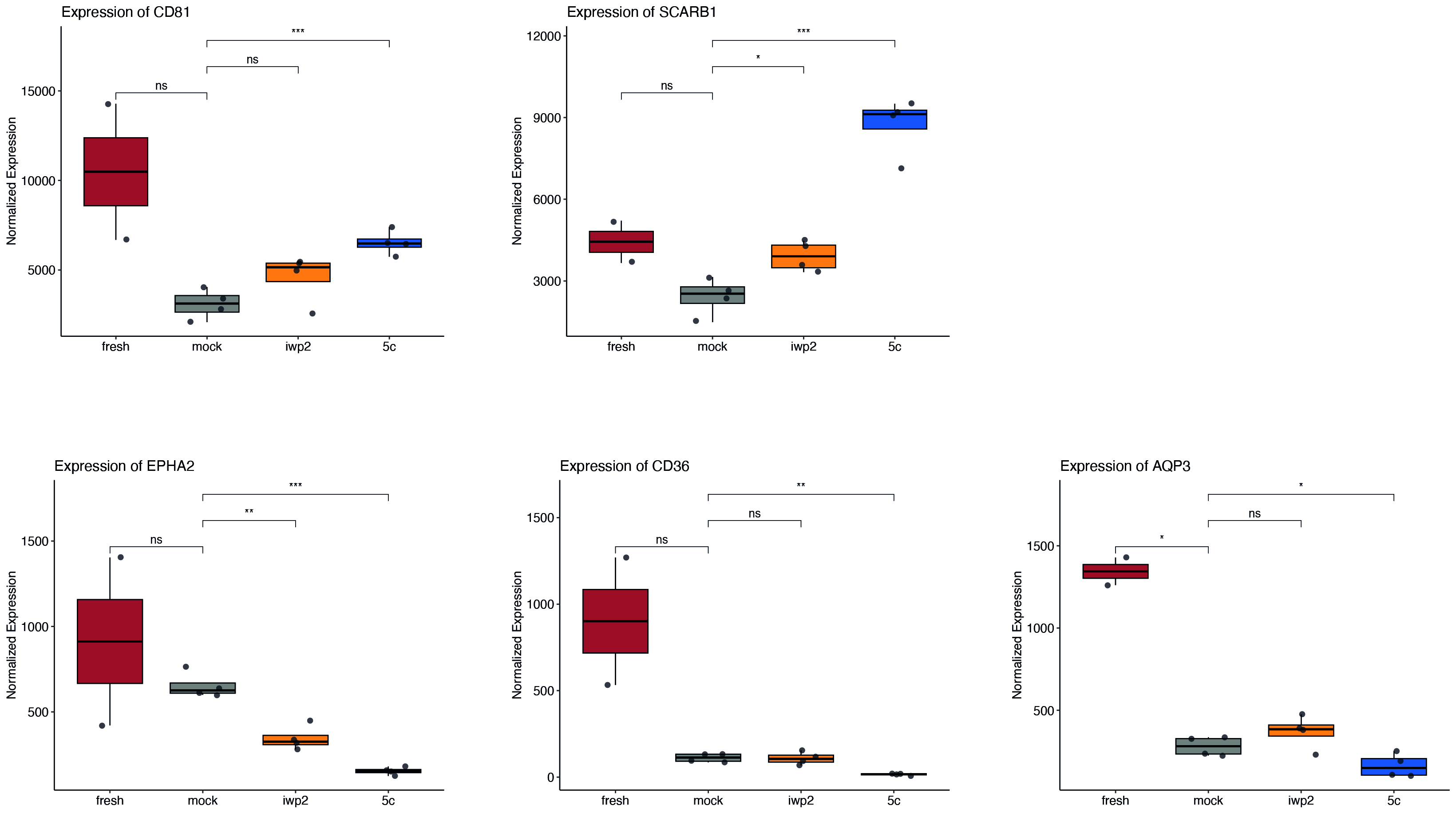

Supplement: S8 Fig — (TIF) [file ppat.1013800.s008.tif]

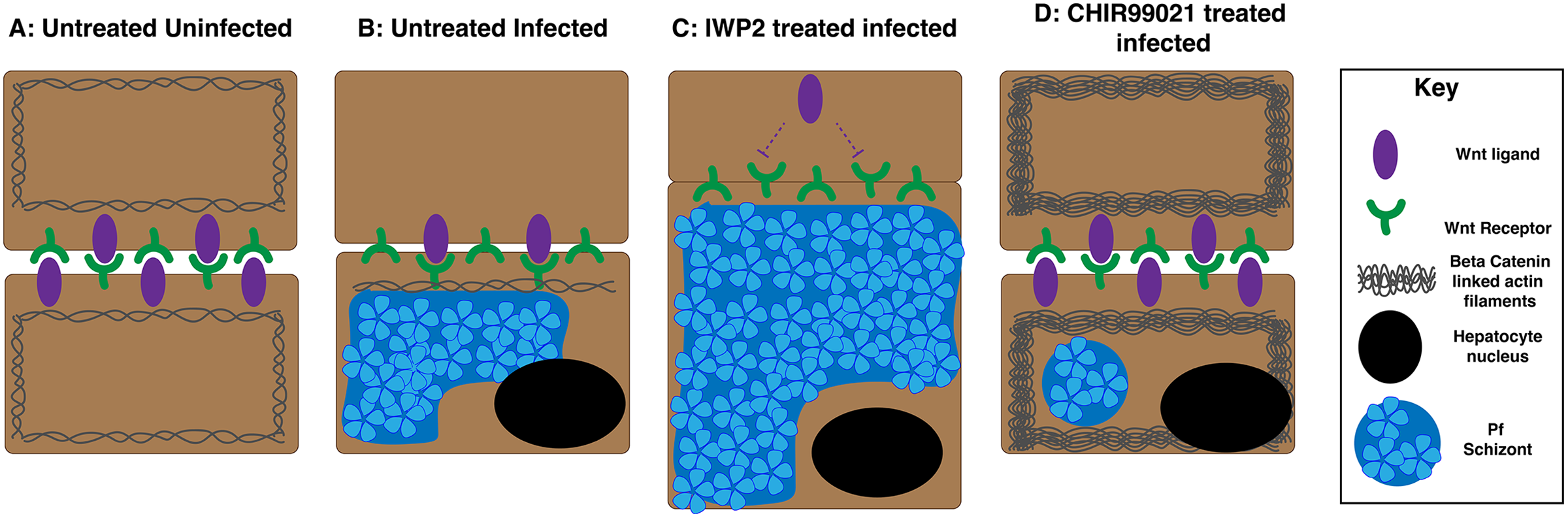

Supplement: S9 Fig — A)Under untreated and uninfected condition, there is a steady level of beta-catenin (β-catenin). In addition to its role in gene transcription, β-catenin is the intracellular component that links the cadherin adhesion molecules to actin filaments and therefore controls the “rigidity” of the cells in relation to its neighbours. B) In an infected hepatocyte (after 3 days post infection), the growing Pf schizont takes up so much of the hepatocyte volume that the trafficking of the Wnt ligands to the surface is disrupted. Wnt receptor of the neighbouring uninfected cells are not activated, and β-catenin molecules are degraded due to the phosphorylation of the enzyme, glycogen synthase kinase 3 (GSK3): this allows some flexibility in the uninfected neighbouring cells to accommodate the growth of the infected cell. However, in these (uninfected cells), Wnt ligands are still present and can interact with the existing Wnt receptors on the infected hepatocytes to maintain some β-catenin in the infected cell, thus limiting the growth/size of the schizont. C) Under IWP2 treatment, Wnt ligands are not trafficked to the surface of both uninfected and infected hepatocytes due to the inhibition of the enzyme porcupine (target of IWP2). Porcupine “labels” (via palmitoylation) Wnt ligands for correct trafficking to the plasma membrane. As a result, Wnt receptors on both uninfected and infected cells are not activated and existing β-catenin are degraded leading to reduced connection between cadherin and actin filaments (i.e., cell-cell contacts) and ultimately reducing rigidity. D) Under CHIR99021 (GSK3 inhibitor) treatment, β-catenin in both uninfected and infected cells cannot be phosphorylated nor degraded. Both cell types are more rigid due to the improve connection between actin filaments and cadherin which severely limits the size of the Pf schizont. (TIF) [file ppat.1013800.s016.tif]
