# Supplementary material for: Inhibition of Wnt signaling in primary human hepatocytes promotes Plasmodium falciparum liver stage development
Source: PLoS Pathog. 2025 Dec 22;21(12):e1013800. doi: 10.1371/journal.ppat.1013800 (PMC12795456; doi:10.1371/journal.ppat.1013800)
Supplement: Attachment — Submitted filename: PPATHOGENS-D-25-00264_R1_REV.pdf [file ppat.1013800.s011.pdf]

PLOS Pathogens

Modulation of host signaling pathways reveal a major role for Wnt signaling in the maturation of Plasmodium falciparum liver schizonts

--Manuscript Draft--

**Manuscript Number:**	PPATHOGENS-D-25-00264R1
**Full Title:**	Modulation of host signaling pathways reveal a major role for Wnt signaling in the maturation of Plasmodium falciparum liver schizonts
**Short Title:**	Changes of host Wnt signalling impacts of the maturation of Plasmodium falciparum liver schizonts
**Article Type:**	Research Article
**Section/Category:**	Parasitology - Protozoa
**Keywords:**	Plasmodium falciparum; malaria; liver stage; hepatocytes; wnt signalling pathway
**Abstract:**	After infection of the human host, the initial stage of the Plasmodium falciparum (Pf) lifecycle takes place in the liver. However, understanding of the host-parasite interaction has been limited by the rapid loss of functionality in cultured primary human hepatocytes (PHHs). Here, we link loss of hepatic functionality to drastic loss in Pf permissiveness, which we effectively prevent by using a novel medium containing serum-replacement and signal transduction inhibitors. Integrating transcriptomic analysis and phenotypic assessment of infection outcome, we identified several host signalling pathways that influence Pf liver stage development. Inhibition of the Wnt pathway in particular plays a major role in determining the size and maturity of Pf-liver schizonts, via retaining metabolic activity and epithelial nature of hepatocytes. Host signalling pathways determining Pf liver stage permissiveness provide insight into the complex host-parasite interaction and may accelerate development of novel therapeutic strategies for Pf-liver stages.
**Additional Information:**	
**Question**	**Response**
**Government Employee**<br><br>Are you or any of the contributing authors an employee of the United States government?<br><br>Manuscripts authored by one or more US Government employees are not copyrighted, but are licensed under a CC0 Public Domain Dedication, which allows unlimited distribution and reuse of the article for any lawful purpose. This is a legal requirement for US Government employees.<br><br>This will be typeset if the manuscript is accepted for publication.	No - No authors are employees of the U.S. government.
**Financial Disclosure**<br><br>Enter a financial disclosure statement that	Yes

describes the sources of funding for the work included in this submission. Review the submission guidelines for detailed requirements. View published research articles from *PLOS Pathogens* for specific examples.<br><br>This statement is required for submission and **will appear in the published article** if the submission is accepted. Please make sure it is accurate.<br><br>**Funded studies**<br>Enter a statement with the following details:<br>• Initials of the authors who received each award<br>• Grant numbers awarded to each author<br>• The full name of each funder<br>• URL of each funder website<br>• Did the sponsors or funders play any role in the study design, data collection and analysis, decision to publish, or preparation of the manuscript?<br><br>Did you receive funding for this work?	
Please add funding details.<br>as follow-up to "**Financial Disclosure**<br><br>Enter a financial disclosure statement that describes the sources of funding for the work included in this submission. Review the submission guidelines for detailed requirements. View published research articles from *PLOS Pathogens* for specific examples.<br><br>This statement is required for submission and **will appear in the published article** if the submission is accepted. Please make sure it is accurate.	NWO | Exacte en Natuurwetenschappen (ENW):Annie Shu-Ping Yang VI.Veni.192.171; ZonMw (Netherlands Organisation for Health Research and Development):Annie Shu-Ping Yang Off Road 04510012010050; ZonMw (Netherlands Organisation for Health Research and Development):Abhishek Kanyal,Richard Bartfai TOP-grant #91218010

**Funded studies**<br>Enter a statement with the following details:<br>• Initials of the authors who received each award<br>• Grant numbers awarded to each author<br>• The full name of each funder<br>• URL of each funder website<br>• Did the sponsors or funders play any role in the study design, data collection and analysis, decision to publish, or preparation of the manuscript?<br><br>Did you receive funding for this work?"	
Please select the country of your main research funder (please select carefully as in some cases this is used in fee calculation).<br>as follow-up to "**Financial Disclosure**<br><br>Enter a financial disclosure statement that describes the sources of funding for the work included in this submission. Review the submission guidelines for detailed requirements. View published research articles from *PLOS Pathogens* for specific examples.<br><br>This statement is required for submission and **will appear in the published article** if the submission is accepted. Please make sure it is accurate.<br><br>**Funded studies**<br>Enter a statement with the following details:<br>• Initials of the authors who received each award<br>• Grant numbers awarded to each author<br>• The full name of each funder<br>• URL of each funder website<br>• Did the sponsors or funders play any role in the study design, data collection and analysis, decision to publish, or preparation of the manuscript?<br><br>Did you receive funding for this work?"	NETHERLANDS - NL
**Competing Interests**	The authors have declared that no competing interests exist.

Powered by Editorial Manager® and ProduXion Manager® from Aries Systems Corporation

On behalf of all authors, disclose any competing interests that could be perceived to bias this work.<br><br>This statement will be typeset if the manuscript is accepted for publication.<br><br>Review the instructions link below and PLOS Pathogens' competing interests policy to determine what information must be disclosed at submission.	
**Data Availability**<br><br>Provide a **Data Availability Statement** in the box below. This statement should detail where the data used in this submission can be accessed. This statement will be typeset if the manuscript is accepted for publication.<br><br>Before publication, authors are required to make all data underlying their findings fully available, without restriction. Review our PLOS Data Policy page for detailed information on this policy. Instructions for writing your Data Availability statement can be accessed via the Instructions link below.	We have now provided a reviewers token to access the RNAseq data which will be made fully public upon acceptance/publication.<br><br>To review GEO accession GSE263643:<br>Go to https://www.ncbi.nlm.nih.gov/geo/query/acc.cgi?acc=GSE263643<br>Enter token ehkrsqmwtdofdmv into the box

We thank all reviewers for their critical, but overall positive evaluation of our manuscript. Below please find our answers to their suggestions and our plan to revise the manuscript accordingly.

Reviewer #1 (Evidence, reproducibility and clarity (Required)):

Challenges associated with growing the liver stage of human malaria parasites in culture is a major roadblock to research. Primary human hepatocytes are currently the model of choice. This manuscript addresses the problem that these cells dedifferentiate quickly in culture, which correlates with a much-reduced ability to support parasite development. They use transcriptomics to describe the dedifferentiation process, conclude a potential role for Wnt signalling as a mediator and demonstrate that the Wnt inhibitor IWP2 preservers the ability of HPPs to support parasite growth over a longer period. They also use a Wnt activator, whose ability to reduce parasite development is less consistent.

Overall, this study provides an interesting chemical fix for increasing culture efficiency for P. falciparum, which labs working with this model may choose to adopt. However, the study does not go beyond presenting pharmacological evidence. Title and abstract claim that the work establishes a causal link between Wnt signalling, hepatocyte differentiation state and parasite development. It is my opinion that claims of a mechanism or causal link need to be supported by more than pharmacological evidence, and that in this case transcriptomics as the only way of assessing the state of the cells is insufficient.

Questions that come to mind are whether nuclear translocation of catenin really occurs in an inhibitor-sensitive way, whether specific known Wnt targets are regulated at the protein/biochemical pathway level and which of these specific targets/pathways are important for parasite development. However, I am no expert on the cell biology of Wnt signalling, neither am I aware of all the tools available.
We appreciate the reviewer's concern about the chemical compounds (namely IWP2 and CHIR99021) and their specificity in targeting Wnt pathway. While we agree with the reviewer that in general chemical inhibition is not a full-proof approach on its own, considering the substantial amount of published literature and our transcriptomics assessment of the excerpted effects (see later), we are confident that IWP2 and CHIR99021 are modulating the Wnt pathway as we claimed by the targeting of key effectors of this pathway.

The original paper by Chen et al[1] characterizing IWP2 as WNT inhibitor showed a decrease in B catenin level after 24 hours at 5uM (10 fold higher than what is used in this study). It also demonstrated IWP2 specificity by showing that overexpression of the compound target, Porcupine can restore the loss of wnt activity (measured using a Wnt/B catenin pathway responsive firefly luciferase reporter) at 0.25, 0.5 and 1uM treatment with IWP2. An extensive but not all-inclusive literature search (n= 9[1-9] including the original Chen et al) showed the usage of IWP2 (0.1uM to 20uM from 24 hours to 5-6 days) on a variety of cell types (mostly cancerous cell-lines with the Wnt/B catenin pathway responsive firefly luciferase reporter) showing either a loss of B catenin and/or loss of luciferase activity due to lack of activation of the reporter construct. We picked a concentration of IWP2 that has been used and published in primary human hepatocytes[9] as it was the most relevant cell type for this study.

With regards to the usage of CHIR99021, the original paper by Ring and colleagues[10] showed that this compound exhibited 500 to >10000 fold selective for its target GSK3; being equally potent to both isoforms of the enzyme but concurrently able to discriminate between GSK and its closest homologues (cdc2 and erk2). There, the authors concluded that CHIR99021 was a highly potent and selective inhibitor of GSK3. Again, an extensive (but not all-inclusive[2,3,6,8,11-14]) literature search shows that CHIR99021 is used on a variety of cell types at a concentration of 2-3 uM (3uM was used in our manuscript). Of note, Lee et al[2] and Arensmen et al[3] have already shown the increase in B catenin levels (in their respective cells used) using western blot, immunofluorescence and/or the transfected Wnt/B catenin pathway responsive firefly luciferase reporter.

Notwithstanding the need for some clarification, as far as they go, the data linking pharmacological agents and parasite development appear sound and I would say the same for the transcriptomic analysis as such. My concern is that neither go deep enough to claim more than preliminary evidence for a link between hepatocyte Wnt signalling and parasite development. I could imagine this manuscript rewritten as a more technical report on how to improve P. falciparum liver stage culture in practice or as a starting point for a deeper study into mechanism. The latter would be needed to support the claims from title and abstract.

With all due respect to the reviewer, we feel that our manuscript goes way beyond a simple technical note. We would have loved to confirm our observations regarding WNT inhibition using genetic tools, but these are very challenging in this experimental system. Genetic deletion of the B-catenin (key component of the wnt signalling i.e. mimicking the IWP2 condition) in mice results in severe defect in hepatocyte maturation[15], and therefore highly likely to prevent the study of Pf liver stage development. We showed previously that Pf sporozoites prefers mature hepatocytes for successful development and maturation[16]. Importantly, efficient overexpression or depletion of target proteins in primary human hepatocytes are non-existent technologies currently. Notwithstanding our conviction that genetic manipulation would yield very similar result, we will mention the potential limitation of chemical inhibitors in the manuscript and indicate the validation via genetic means is desired in the future.

Furthermore, we now also provide data demonstrating that Pf parasites do not show differences in size when grown in PHHs with pre-treatment with IWP2 or CHIR99021 but then returned to the standard growth media of WBH (Figure S5) i.e. the chemical (ant)agonists must be present during parasite development to have the indicated impact. This further cements the importance of Wnt pathway in determining parasite development.

Major points

The rapid dedifferentiation of primary human hepatocytes poses a challenge to their application in toxicology, studies of human liver functions and to develop 3D tissue culture models. How to maintain hepatic functions is a long-standing problem and previous attempts at solving it have included small molecules to maintain the cells

differentiated state for longer (for instance PMID: 31432507). The author here use development of P. falciparum liver stages as readout, but there must be a broader context of published work from other uses of PHHs, which the authors should consider, including possibly transcriptomics. A quick search of GEO finds >2000 projects related to "primary human hepatocytes", for instance PMID: 36241695. The authors appear to be taking a rather narrow view of past work. Placing their data in a broader context would help the non-expert feel reassured they are not reinventing the wheel.

We are certainly agree with the reviewer that there are plentiful transcriptomic data available in the literature and might be relevant to use. However, with all honesty, we are unclear as to what the reviewer is proposing here. To the best of our knowledge there is no transcriptomic data available in the literature using these specific inhibitors on PHHs, but even if such data would be available, given potential donor and experimental condition-specific differences, we feel it is still valuable to repeat these experiments in the very same cell culture system where the infections are also performed.

What does it mean in terms of mechanism if Wnt signalling is associated with the transcriptomic signature of the cultured cells. Are genes of the signalling pathway enriched or beta-catenin targets, for instance?

The most likely effect of the repression of the signalling pathway is the downregulation of associated target genes and consequent alteration of the cellular state. To assess this we have reanalysed the RNA-seq data and investigated the relative activity of transcription factors (via quantifying the deregulation of their targets genes). As expected the Wnt-associated transcription factors (e.g. beta-catenin/CTNNB1, TFC7L2) displayed increased activity during *in vitro* culturing and most of their target genes displayed clear down-regulation upon IWP2 treatment (**Figure 2**). Next to these we also observed altered activity of the SMAD (TGFb) and AP1 (noncanonical WNT) transcription factors, which is likely due to indirect effect of WNT deregulation. This is in agreement with published literature stating the interlinked nature of the two pathways (WNT and TGFb). However, given the lack of impact of TGFb inhibitor (SB) alone on schizont size (Figure 2G of the manuscript), we are confident of the WNT pathway's impact on parasite development.

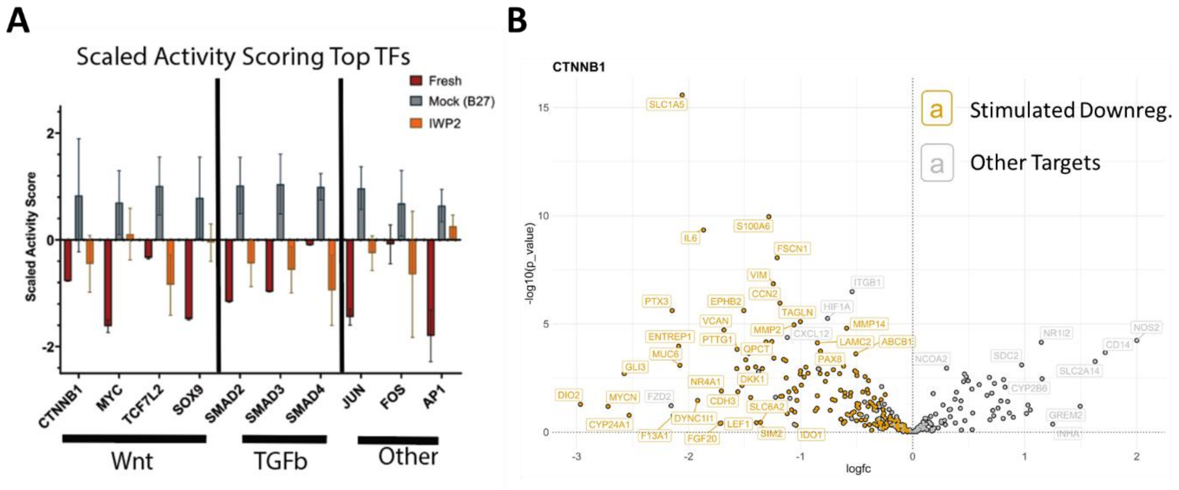

***Figure 2**: **IWP2 treatment does supress expression of WNT associate transcription factor target genes**. **A)** Scaled activity scores for canonical Wnt/TGFβ signalling and non-canonical Wnt (AP1 family) transcription factors. **B)** Volcano plot depicting the deregulated expression of CTNNB1(beta-catenin) target genes in IWP2 treated cells compared to culture in B27 supplemented medium. Target genes which are stimulated by CTNNB1 and downregulated in IWP2 treatment (demonstrating at suppression of CTNNB1 activity) are highlighted in amber.*

Next to changes in WNT target gene expression IWP2 treatment also reduced the expression of core WNT pathway components (e.g. ligand WNT, receptor: Frizzled) providing further evidence for the effect of IWP2 on this pathway.

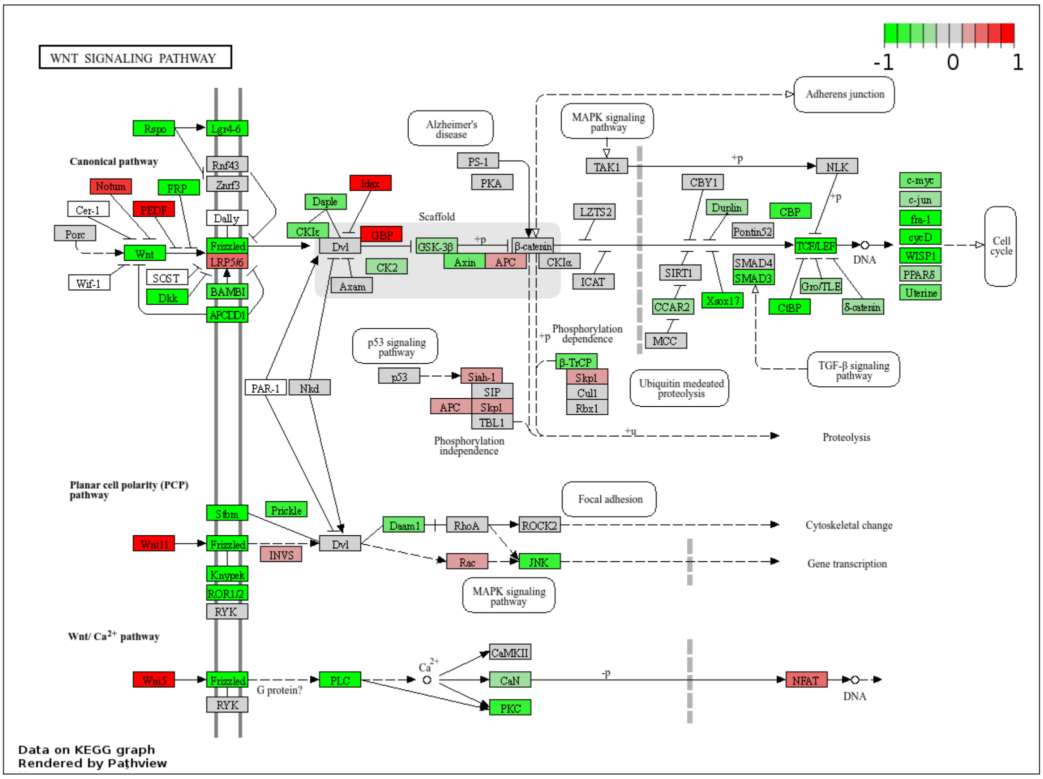

***Figure 3**: Schematic of elements of Wnt signalling pathway deregulated in IWP2-treated vs mock treated cultured PHHs. Overall decreased expression of several canon signalling elements is highlighted in green and upregulation of pathway inhibitors in red.*

I am not convinced from Fig. 3G that the treated cells are transcriptomically more like the starting condition than the mock treated cells. The statement in line 155 that "that IWP2 suppressed the transcriptional changes observed during the in vitro culture in B27" needs to be qualified and supported with data. The populations overlap on the PCA and both are clearly distinct from the starting condition. They go on to show through analysis of the transcriptomic data that differences between treated vs untreated cells are consistent with reduced Wnt signalling and less dedifferentiation. However, from the PCA plot this would appear to be on a background of much variation. Can the authors come up with a better measure of overall similary?

Based on spatial proximity in the PCA space, B27 cultured cells were distinct from fresh hepatocytes (Figure 3G in the manuscript). Upon IWP2 treatment we observed a notable shift of cultured samples towards fresh samples on the PCA space. To quantify this, we calculated the sample distance scores which further indicated

increased similarity between IWP2-treated cells and fresh hepatocyte (mean distance score: 156) as compared to mock treatment (mean distance score: 137) (Note that lower distance scores define higher similarity between pairwise samples under comparison). Furthermore, treatments with IWP2 suppressed the deregulation of many genes that were otherwise upregulated during *in vitro* culture in B27 medium (**Figure 3**). We will include these extra information in the revised manuscript to further substantiate our conclusion.

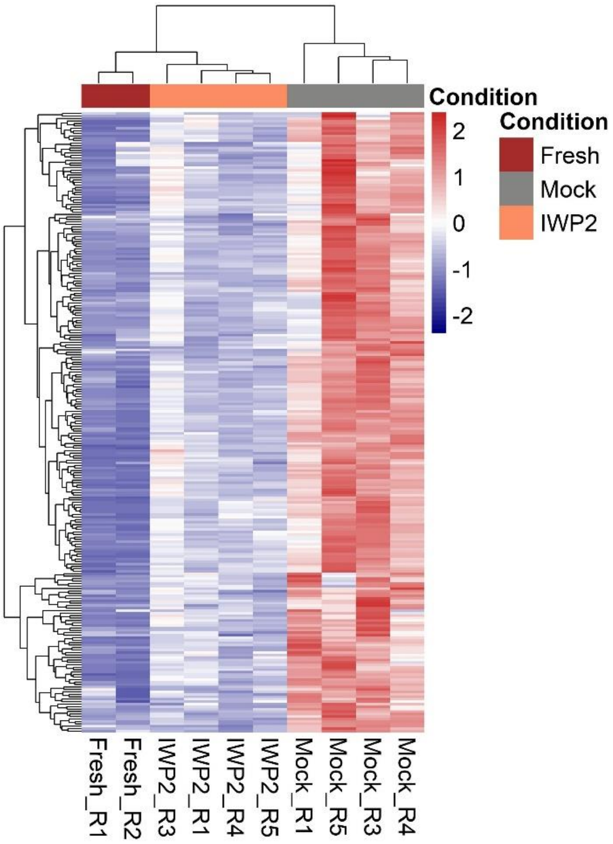

Figure 4: Upregulation of numerous genes upregulated during* in vitro *culture is effectively inhibited by IWP2 treatment. *Gene expression heatmap of fresh (brown) and in vitro cultured mock as well as IWP2 treated PHH samples (orange and grey, respectively). The gene set under investigation here is restricted to genes originally identified to be upregulated in in vitro cultured PHHs compared to fresh hepatocytes. As apparent from the heatmap, upregulation of these genes is efficiently suppressed by IWP2 treatment.*

Minor points:

Errors related to numerus are in the title and in lines 63, 126, 142, Fig. S1 legend.

Incomplete sentences or sentences needing rephrasing starting lines 65, 96, 147, 210, 224

Line 99: Why "return"? Use more precise language. Once gone, schizonts cannot be recreated.

Line 101: "to into"?

Line 116: "cannot fully prevent loss of hepatic functions"... Rephrase. Functions were not measured.

Line 153: reference to Fig. 4A must be 3G.

Line 193: How does Fig. S4 relate to "all these up-regulated pathways"? The authors

should state which pathways and which of their data they mean. Is this about the inhibitor cocktail? What if its effect is off target and not due to the precise combined inhibition of ""all these up-regulated pathways"? There is not much one can conclude from an inhibitor cocktail or even a single pharmacological agent.
We have now added “Furthermore, 5C treatment appear to exert transcriptional and/or cellular changes beyond the prevention of EMT (data not shown).”

Fig. S1: Panel lettering in the figure and legend do not match.
In the legend, the sencence starting "As for..." is garbled. Also, what is "per replicate (2 replicates)" and how does it relate to there being more than 2 dots? What is ***?
We have created an excel sheet (Supplementary information) where all the statistical tests and their corresponding p values are listed per figure. We hope that creates more clarity.

Fig. S4: Rephrase "Data show mean" and provide a legend for A and B.

We thank the reviewer for all the minor points raised, which we will all effectively address in the revised manuscript.

Referees cross-commenting

The reviews do not strike me as incongruous, in that there is general agreement that the experimental work is sound and of practical utility for parasitologists studying Pf liver stages.

Whether one accepts pharmacological data as sufficient to claim a mechanistic insight is a matter of judgement. I would not be comfortable with this work entering the literature as evidence for a role for Wnt signalling in host-parasite interactions, as implied in the title because, a) it only investigates how to fix the problem with a culture system and b) it does so without the rigor human cell biologists can apply to questions of signal transduction and cellular differentiation.

I'd have no problem with this if the authors wanted to publish their work under the title "An inhibitor of Wnt signalling improves Pf development in primary human hepatocytes". The challenge I see is that journals using Review Commons tend to want mechanistic insight.

Reviewer #1 (Significance (Required)):
Time required for revision depends on whether the authors choose to revise their presentation and claims or provide deeper insights into mechanism of the pharmacological agent used.

Reviewer #2 (Evidence, reproducibility and clarity (Required)):
This paper reports on the role of cellular state of hepatocytes on their infectability by the human malaria parasite P. falciparum. The authors find that longer cultivation of primary hepatocytes leads to a drop in infectivity by parasites. Several gene regulatory networks were found to be associated to this phenomenon and blocking of some of them, most prominently wnt signalling affects the development of P. falciparum. The data are clear and statistically significant

The paper is well written and nearly ready to publish. I only have a few minor comments:

Line 57: rephrase Eph A2 or also cite the study calling this discovery into question
We will cite the study calling this discovery into question.

Figure 1:
Please be coherent in writing, e.g. schizonts vs Schizonts in Y-axis of panels
We will correct this inconsistency.

Figure panels 1F-H , 2A, 3G-J: not sure the grey bars on top and right of panel are needed. I would advise to delete
We are uncertain as to which grey bar the reviewer is referring to and these might only be visible after pdf conversion or in a specific computer environment. Would these still appear in the revised manuscript we will find a way to delete them.

Figure 5, should the label 'b-catenin' not be changed to 'b-catenin linked actin filaments' (or similar)?
We will change the “b-catenin” to “b-catenin linked actin filaments”.

The paper will be interested to researchers investigating the wnt signaling pathway (>42000 Pubmed entries on 'wnt signalling') and malaria liver stages (>2000 Pubmed entries). Hence interest should transcend the infectious disease community.
I am working on the cell biology of malaria parasites.

Referees cross-commenting

I agree with the note of reviewer 1.

Reviewer #2 (Significance (Required)):

in this paper the authors provide a well conducted study in the effects of de-differentiation of primary hepatocytes for their capacity to serve as host cells for P. falciparum, the parasite eliciting the most deadly form of human malaria. The parasite undergo a massive first expansion in the liver, which is clinically silent, before they burst out into the blood stream, where they cause the symptoms of this disease. The molecular details of parasite-host cell crosstalk in the liver are studied by only a few brave groups due to the fact that parasites need to be grown in mosquitoes in high security insectaries and primary liver cells are also not easy to get. The paper is the first to my knowledge that implements wnt signaling, an ancient signaling cascade, in the growth of liver stage parasites using a mix of infection studies and transcriptome analysis.
We thank the reviewer for his/her kind comments regarding the importance of finding Wnt signalling impact on the growth of liver stage parasites and its potential follow-on impact on other liver pathogens.

Reviewer #3 (Evidence, reproducibility and clarity (Required)):

In this study, the authors address the host-parasite interaction in the context of malaria liver stage and more precisely which host signaling pathways could be involved in the development of the intracellular Plasmodium parasite within human hepatocytes. To do so, they use an in vitro culture system relying on freshly isolated primary human hepatocytes (PHH) infected at different time points post-plating with Plasmodium falciparum sporozoites.

- for all the study they compare the susceptibility of PHH to different strains of P. falciparum (PfNF54, PfNF175 and PfNF135) which are known for displaying different levels of infection and growth kinetics in PHH;
- they use appropriate markers for estimating liver stage parasite maturation : the size of the intracellular schizont, the number of parasite nuclei contained in the schizont and the expression of a middle-late liver stage antigen, the MSP1 protein; for estimating hepatocyte quality they chose the number of nuclei as a surrogate for cell viability and human glutamine synthetase expression for quantitative analysis of hepatocyte health;
- as stated by the authors, it is indeed well known that PHH rapidly loose their phenotype after in vitro culture. Their first show that PHH susceptibility to P. falciparum (Pf) infection, regardless of the PHH batch and Pf strain, is severely diminished as soon as day 5-7 post-hepatocyte plating (p.p.) compared to day 2 and this is not due to a decrease in hepatocyte survival;
- then they identify a culture medium (referred as "B27") that improves infection levels and hepatocyte survival up to day 7 post-plating but still these two parameters dramatically decline after day 7 p.p..
- in order to identify the hepatocyte factors that would be responsible for this loss in differentiated hepatocyte phenotype and Pf susceptibility, the authors compared the transcriptomic profiles of uninfected PHH on the day of isolation (day 1) with day 7;
- their strategy then was to counterbalance the upregulation of some genes observed on day 7 to try to recover the phenotype of day 1 PHH. They thus identified 5 pathways (Wnt, TGF-b, Notch, BMP, Adenylyl-cyclase) and tested each using specific antagonists. Their data are consistent with those formerly published by Xiang et al (Nat Com 2019);
- The antagonists for the TGF-beta and Wnt pathways were the two showing the most significant effect on parasite's number, size and maturation (MSP1+), as well as hGS expression by infected cell. However the mix of the 5 molecules, previously described to promote functional maintenance of PHH in vitro together with VHB replication life cycle (Xiang et al, Nat Com 2019), did not improve susceptibility to Pf nor its development within treated PHH;
- Then, they confirmed that the transcriptome of PHH treated or not with Wnt antagonists are different and highlight genes and biological processes that are either upregulated or suppressed in Wnt antagonist treated cells;
- Finally, they confirm that when hepatocytes are treated with a Wnt activator then parasite number and size are decreased albeit to a lesser extent when compared to the opposite effect of Wnt inhibitor.

Major comments:

- Are the key conclusions convincing? Yes.

- Should the authors qualify some of their claims as preliminary or speculative, or remove them altogether? No, excepted maybe the Figure 5 which can be considered too much speculative.
Figure 5 provides mechanistic insight that and a potential explanation of how the molecular effectors of the Wnt pathway interact to regulate parasite growth and development. It is (what we feel) as a nice summary to end with.

- Would additional experiments be essential to support the claims of the paper? Request additional experiments only where necessary for the paper as it is, and do not ask authors to open new lines of experimentation.
The effect of the different treatments on hepatocyte health could be more documented (nuclei number shown for all tested experimental conditions and/or LDH quantification in the supernatant to assess for cell viability and/or albumin quantification in the supernatant.
We agree with the reviewer that such extra information could be useful, but we feel that it is not strictly essential to support our main conclusions as regards to parasite growth. Notably, providing such data would require repeating the experiments (since supernatants are not preserved), an effort, in our opinion, not proportional to the information gained. We will however, provide numbers of hepatocyte count as suggested below.

- Are the suggested experiments realistic in terms of time and resources? It would help if you could add an estimated cost and time investment for substantial experiments.
Measure of nuclei number should be accessible without the need for performing additional experiments as long as the authors stored the plates. Other experiments could be possible if the authors stored culture supernatants frozen; also both LDH and Albumin quantification are quite expensive.

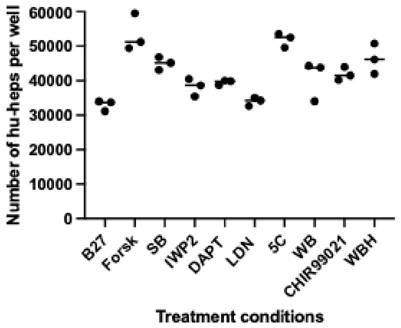

We have analysed the existing data to show the number of hepatocytes per well as a measure of hepatocyte health. We will insert this appropriately in a supplementary figure. This graph has been now split into Figure S2, 3 and 4, depending on the treatment condition.

- Are the data and the methods presented in such a way that they can be reproduced? Yes. The authors should specify how many sporozoites they inoculate to the hepatocyte cultures and how often they renew the culture medium.
We agree with the reviewer and will add these details to the methods section.

- Are the experiments adequately replicated and statistical analysis adequate? The number of replicates allow to have confidence in the data. This represents a big amount of work given the nature of the study, e.g. working with freshly isolated PHH and mosquitoes infected with different strains of Pf. One of the challenge when working

with fresh human hepatocytes is the variability from one batch to another. Here the authors provide data from three independent experiments per results which can be considered sufficiently robust and moreover they obtain consistent results for these different batches, which also consolidates the data.
We thank the reviewer for recognising the challenge with working with fresh human hepatocytes and with getting consistent data despite the donor-to-donor variations. Like you mentioned, the consistency achieved in this data set irrespective of the donor gave us great confidence (despite lack of genetic data) that the wnt pathway is involved in Pf liver stage development.

- Other major comment:
The authors should explain how they chose the antagonists or activator that they use and also to which extent they are specific for each pathway. They also should specify how they chose the concentration of each antagonist that they use. The authors should explain why they chose to treat PHH with Wnt antagonist for a duration of 6-8 days before performing infection with Pf parasites.
We apologise for lack of details in the antagonist and activator used in this paper. We will rectify in the introduction/discussion (see reviewer 1, point 1). Similarly, we will motivate in the result section why we treated for 6-8 days: based on Figure 1B, untreated PHH showed the lowest permissibility for parasite development (lowest number of schizonts) by day 7 post plating, therefore we wanted to make sure the treatment was robust enough to overcome this phenomenon. The range of treatment days were due to practical/logistic reasons such as when sporozoites or primary human hepatocytes are available.

Minor comments: We agree with the reviewer's minor comments and will address accordingly.

- Specific experimental issues that are easily addressable. See above.
Host nuclei counts are now provided. (see Figure S2-4).

- Are prior studies referenced appropriately? Yes albeit the authors could have discussed their data in comparison with those of other transcriptomic studies on human malaria liver stage addressing the response of the hepatocyte to the infection.
The majority of these studies are performed in the rodent models of malaria and may not be translatable.

- Are the text and figures clear and accurate?

Yes but there are many sentences that could benefit from rewriting/reformulation to gain in clarity, mainly in the results section:
Lines 120-122: "Testing the potential relevance of specific signalling pathways in culture-induced upregulation of genes (Figure 1F) showed a strong enrichment of genes associated with Wnt signalling."

Line 134: "DAPT-treated hepatocytes contained parasites displayed limited, but significant.."

Lines 137-138: "Similarly, in IWP2-treated hepatocytes schizonts showed improvements especially in size ..."

Lines 147-148: "PHHs were treated with either IWP2 (6 days) prior to and during Pf infection"; the authors should remove "either" from the sentence

Line 195: "appear" should be corrected for "appears"

- Do you have suggestions that would help the authors improve the presentation of their data and conclusions?
No.

Reviewer #3 (Significance (Required)):

- Describe the nature and significance of the advance (e.g. conceptual, technical, clinical) for the field.

The nature of the advance is conceptual. This work provides interesting results regarding the biological pathways of the hepatocyte that are involved in the development of Plasmodium falciparum, a field for which very little is known. By highlighting the role of the Wnt pathway on the parasite development it provides new data to the field of research in malaria liver stage.

- Place the work in the context of the existing literature (provide references, where appropriate).
There is very few studies addressing the interactions between hepatocyte and Plasmodium falciparum during the liver stage.

- State what audience might be interested in and influenced by the reported findings.
This work will be beneficial for the community working on malaria liver stage, for basic research but could also provide new avenues of research aiming at identifying novel antimalarial therapies.

- Define your field of expertise with a few keywords to help the authors contextualize your point of view. Indicate if there are any parts of the paper that you do not have sufficient expertise to evaluate.

The key words describing my area of expertise are:
Malaria liver stage, Plasmodium species that infect humans, PHH, immunity, in vitro and in vivo models.

I do not have sufficient expertise in RNA seq analyses to evaluate this part of the work.
We thank the reviewer for his/her recognition of the strength of our dataset, for highlighting the lack of existing knowledge on Pf liver stage development and therefore the impact of our findings for the field.

1 Chen, B. *et al.* Small molecule-mediated disruption of Wnt-dependent signaling in tissue regeneration and cancer. *Nat Chem Biol* **5**, 100-107, doi:10.1038/nchembio.137 (2009).

2 Lee, H. J., Wolosin, J. M. & Chung, S. H. Divergent effects of Wnt/beta-catenin signaling modifiers on the preservation of human limbal epithelial progenitors according to culture condition. *Sci Rep* **7**, 15241, doi:10.1038/s41598-017-15454-x (2017).

3 Arensman, M. D. *et al.* WNT7B mediates autocrine Wnt/beta-catenin signaling and anchorage-independent growth in pancreatic adenocarcinoma. *Oncogene* **33**, 899-908, doi:10.1038/onc.2013.23 (2014).

4 Kleszcz, R., Szymanska, A., Krajka-Kuzniak, V., Baer-Dubowska, W. & Paluszczak, J. Inhibition of CBP/beta-catenin and porcupine attenuates Wnt signaling and induces apoptosis in head and neck carcinoma cells. *Cell Oncol (Dordr)* **42**, 505-520, doi:10.1007/s13402-019-00440-4 (2019).

5 Zhang, J. *et al.* DKK1 promotes migration and invasion of non-small cell lung cancer via beta-catenin signaling pathway. *Tumour Biol* **39**, 1010428317703820, doi:10.1177/1010428317703820 (2017).

6 Shin, Y. S. *et al.* The Wnt/beta-catenin signaling pathway plays a role in drug-induced liver injury by regulating cytochrome P450 2E1 expression. *Toxicol Res* **39**, 443-453, doi:10.1007/s43188-023-00180-6 (2023).

7 Janda, C. Y. *et al.* Surrogate Wnt agonists that phenocopy canonical Wnt and beta-catenin signalling. *Nature* **545**, 234-237, doi:10.1038/nature22306 (2017).

8 Xu, H. *et al.* Unfolded protein response is required for the definitive endodermal specification of mouse embryonic stem cells via Smad2 and beta-catenin signaling. *J Biol Chem* **289**, 26290-26301, doi:10.1074/jbc.M114.572560 (2014).

9 Xiang, C. *et al.* Long-term functional maintenance of primary human hepatocytes in vitro. *Science* **364**, 399-402, doi:10.1126/science.aau7307 (2019).

10 Ring, D. B. *et al.* Selective glycogen synthase kinase 3 inhibitors potentiate insulin activation of glucose transport and utilization in vitro and in vivo. *Diabetes* **52**, 588-595, doi:10.2337/diabetes.52.3.588 (2003).

11 Huang, J., Guo, X., Li, W. & Zhang, H. Activation of Wnt/beta-catenin signalling via GSK3 inhibitors direct differentiation of human adipose stem cells into functional hepatocytes. *Sci Rep* **7**, 40716, doi:10.1038/srep40716 (2017).

12 Touboul, T. *et al.* Stage-specific regulation of the WNT/beta-catenin pathway enhances differentiation of hESCs into hepatocytes. *J Hepatol* **64**, 1315-1326, doi:10.1016/j.jhep.2016.02.028 (2016).

13 Ma, Y. *et al.* CHIR-99021 regulates mitochondrial remodelling via beta-catenin signalling and miRNA expression during endodermal differentiation. *J Cell Sci* **132**, doi:10.1242/jcs.229948 (2019).

14 Jin, C., Samuelson, L., Cui, C. B., Sun, Y. & Gerber, D. A. MAPK/ERK and Wnt/beta-Catenin pathways are synergistically involved in proliferation of Sca-1 positive hepatic progenitor cells. *Biochem Biophys Res Commun* **409**, 803-807, doi:10.1016/j.bbrc.2011.05.094 (2011).

15 Tan, X. *et al.* Beta-catenin deletion in hepatoblasts disrupts hepatic morphogenesis and survival during mouse development. *Hepatology* **47**, 1667-1679, doi:10.1002/hep.22225 (2008).

16 Yang, A. S. P. *et al.* Development of Plasmodium falciparum liver-stages in hepatocytes derived from human fetal liver organoid cultures. *Nat Commun* **14**, 4631, doi:10.1038/s41467-023-40298-7 (2023).

Manuscript (clean)

Click here to access/download;Manuscript;Kanyal_manuscript_clean_20240

Modulation of host signaling pathways reveal a major role for Wnt signaling in the maturation of *Plasmodium falciparum* liver schizonts

Abhishek Kanyal[1]*, Geert-Jan van Gemert[2]*, Haoyu Wu[1,3]*, Alex van der Starre[2], Johannes HW de Wilt[4], Teun Bousema[2], Robert W. Sauerwein[2,], Richard Bartfai[1]*, Annie SP Yang[2,5,6]*

*these authors contributed equally

1. Department of Molecular Biology, Faculty of Science, Radboud Institute for Molecular Life Science, Radboud University, Nijmegen, The Netherlands

2. Radboud Center of Infectious Diseases, Department of Medical Microbiology, Radboud University Medical Center, Nijmegen, the Netherlands

3. CAS Key Laboratory of Regenerative Biology, Guangzhou Institutes of Biomedicine and Health, Chinese Academy of Sciences, Guangzhou 510530, China

4. Department of Surgery, Radboud University Medical Center, Nijmegen, the Netherlands

5. Present address: Leiden University Center for Infectious Diseases (LUCID), Leiden University Medical Center, Leiden, the Netherlands

6. Lead contact.

Correspondence: a.s.yang@lumc.nl (ASPY) or r.bartfai@science.ru.nl (RB)

Abstract:

After infection of the human host, the initial stage of the *Plasmodium falciparum* (Pf) lifecycle takes place in the liver. However, understanding of the host-parasite interaction has been limited by the rapid loss of functionality in cultured primary human hepatocytes (PHHs). Here, we link loss of hepatic functionality to drastic loss in Pf permissiveness, which we effectively prevent by using a novel medium containing serum-replacement and signal transduction inhibitors. Integrating transcriptomic analysis and phenotypic assessment of infection outcome, we identified several host signaling pathways that influence Pf liver stage development. Inhibition of the Wnt pathway in particular plays a major role in determining the size and maturity of Pf-liver schizonts, via retaining metabolic activity and epithelial nature of hepatocytes. Host signaling pathways determining Pf liver stage permissiveness provide insight into the complex host-parasite interaction and may accelerate development of novel therapeutic strategies for Pf-liver stages.

Authors' summary

Despite substantial elimination and control measures malaria still causes substantial mortality and global disease burden. Liver stage development of the malaria parasite is a critical first step preceding clinical symptoms and is the prime target of antimalarial vaccine/drug development. ~~We, h~~owever, lack sufficient understanding of critical components of the host liver environment supporting or preventing parasite development. In this study, we observed substantial decline in the ability of cultured human hepatocytes to support parasite development and identified molecular signatures and altered activity of host signalling pathways associated with the loss of permissiveness. ~~We,~~ ~~in particular,~~ demonstrate the substantial impact of the Wnt pathway on the growth and maturation of liver-stage parasites. We hence provide insight into how host tissue homeostasis can impact ~~on~~ parasite development and pave the way for further dissection of host pathogen interaction and development of improved intervention strategies.

Introduction

The mosquito-borne disease, malaria, remains a devastating global burden with approximately 250 million cases and 620,000 deaths annually [1]. Most of the deaths are due to the parasite *Plasmodium falciparum* (Pf), which begins its developmental life-cycle inside liver cells (hepatocytes) after initial deposition into the skin by an infected mosquito. Over a period of approximately one week, a small number of these parasites (sporozoites) will replicate and differentiate into intracellular schizont, each containing approximately 90000 blood-infective daughter merozoites [2]. The release of these merozoites into the circulation and their subsequent cyclical asexual replication in red blood cells (RBCs) is responsible for clinical symptoms associated with the disease.

Despite its fundamental importance in enabling effective infection, ~~currently fundamental~~ understanding of the complex parasite-host interaction within ~~the~~ infected hepatocytes remains elusive. Studies with the rodent *Plasmodium berghei* (Pb) model have been informative as a proxy for Pf but findings are often not translatable to Pf-host interactions ~~as in particular~~ the rodent parasite has only a 2-day liver-stage period compared to 7-days for Pf [3]. Specific host membrane receptors have been identified to be involved in Pb sporozoite invasion of hepatocytes ~~such as~~ CD81 and Scavenger Receptor B1 (SR-B1) [4-6]; similarly, host factors involved in nutrient acquisition [7-10] and/or prevention of host cell death pathways [11-14].

Research in Pf primarily involves *in vivo* models such as mice with humanised liver [2, 15, 16] or *in vitro* models where hepatocyte cell-lines such as HC-04 [17, 18] and primary human hepatocytes (PHHs) are used. The usage of cell-lines has been limited to the identification of host receptors involved in parasite invasion such as EphA2 [4, 19] and glypican 3 [18]. ~~Contrastingly,~~ PHHs are considered the gold standard in drug assays [3, 20] and used to identify host factors needed for the development of Pf liver schizont such as SR-B1 [10, 21, 22] and glutamine synthetase [23]. A well-known phenomenon of *in vitro* cultured PHHs, however, is the quick loss of hepatic features [24, 25], yet ~~its~~ impact has not been explored ~~in Pf liver stages~~ and may hinder the discovery of host factors that are important for full maturation of liver schizonts. Establishment of an *in vitro* hepatocyte model that preserve hepatocyte features is therefore a prerogative ~~and the next logical frontier in research~~. The loss of functional hepatic features is controlled by specific host signaling pathways [24] of which, their potential impact on parasite development have not been previously examined.

Here, we set out to 1) identify the impact of loss of hepatic functions in ~~*in vitro* hepatocytes~~ and their permissiveness to Pf infection, 2) improve and stabilize hepatocyte culture conditions for Pf permissiveness, facilitating complete schizont development and 3) identify host pathways in hepatocytes that influence the developmental kinetics of Pf liver stages. Using RNA sequencing to compare the transcriptomics profile between ~~the~~ freshly isolated and cultured PHH, we identified upregulation of the Wnt (Wingless and Int 1), TGF-β (Transforming Growth Factor Beta), Notch and Rho signaling pathways. Treatment with specific chemical antagonists of some pathways led to improvement in Pf schizont development ~~for some pathways~~. Furthermore, the Wnt pathway was shown to be most influential in determining the size of the ~~growing~~ Pf schizont.

Results~~:~~

In vitro cultured PHHs lose permissibility for Pf liver stage infection and development

Traditionally fresh ~~primary human hepatocytes (~~PHH~~)~~ are cultured in standard medium containing human sera (WBH) [23, 26, 27] and infected with Pf ~~at~~ 48 hours after plating. However, cultured hepatocytes quickly lose their cellular identity, and this may impact on the host cells' permissibility to sustain Pf infection. To investigate the effect of longer culturing, we infected PHH at different time points post-plating (p.p) with different Pf strains with different developmental kinetics (PfNF54, PfNF175 and PfNF135 [23]); schizont numbers

were evaluated at five days post-infection (p.i) (Figure 1A). We observed a sharp decrease in number of schizonts in relation to the p.p. period for all Pf strains tested with the most drastic decline occurring between days 2 and 5 p.p. This reduction in the number of infected cells could not be explained by a decrease in the number of viable hepatocytes, which became prominent only after day 9 p.p (Figure 1C).

A previous study has shown that medium containing human sera drastically affects function of hepatocytes [21]. Therefore, we examined the impact of sera on hepatocyte permissiveness for parasite development via the removal of sera (WB versus WBH) (Figure S1). Only mild improvements in schizont numbers and the presence of the maturation marker PfMSP1 were observed in sera negative medium (WB). We hence concluded that a novel hepatic culture medium is needed to restore schizont numbers to those numbers seen on day 2 p.p. PHH. A serum replacement supplement (B27) on a William's E Glutamax base was tested given its beneficial effect for differentiation of stem cells [28] and hepatic organoids into hepatocytes [29, 30] as well as other hepatic culture systems [24]. Addition of B27 to into the basal medium William's E Glutamax substantially delayed the loss of hepatocyte permissiveness by at least 7 days (Figure 1D). However, this was not due to changes in hepatocyte numbers which remained comparable to the WBH medium thus reflecting a change in the phenotype of the PHHs (Figure 1E).

Irrespective of these positive effects on Pf infections, uninfected PHHs cultured for 7 days displayed a transcriptional profile that was vastly different from freshly isolated PHH showing an upregulation of 2420 gene transcripts and a down regulation of 2756 genes (Figure 1G). The most significantly up-regulated genes were those involved in actin filament organization, cell-substrate adhesions, wound healing and extracellular matrix organization (Figure 1H). All these pathways are classically associated with epithelial to mesenchymal transition (EMT) [3]. As for down-regulated genes, we found a strong enrichment for those involved in metabolism i.e. fatty acid metabolism, lipid catabolism, organic anion transport and alcohol metabolism among others (Figure 1F). Together, while serum replacement (via B27) has a substantial positive effect on PHH permissiveness to Pf infection, it cannot fully prevent the loss of hepatic functions, which may be potentially relevant for the maturation of liver schizonts.

Specific host signal transduction pathways influence Pf liver stage development

Transcriptomic data (Figure 1F) showed a strong enrichment of genes associated with Wnt (Wingless and Int-1) signaling, Ras protein signaling, transforming growth factor beta (TGF-β), apoptotic signaling pathway and Notch signaling pathways (Figure 2A). Compared to the fresh PHH, genes involved in the Wnt, TGF-β and Notch pathways were strongly upregulated (Figure 2B-D), while BMP and Adenylyl-cyclase pathways mildly upregulated in B27 cultured PHH (Figure 2E). To examine the impact of these host pathway on Pf liver stage development, we treated PHH for 6-8 days p.p., with selective modulators of the individual pathways (IWP2, SB431542, DAPT, LDN193189 and Forskolin) (Figure 2F-J). Quantitative measurements assessed the replication (size, nuclear DNA content via DAPI; Figure 2F-G), health (human glutamine synthetase: hGS; Figure S2B-C) and maturation (PfMSP1; Figure 2I) status of the schizonts, respectively (Figure 2F-J, Figure S3). Modulation of both the BMP and the adenylyl-cyclase pathways through the usage of LDN193189 and Forskolin, respectively did not result in significant improvements in ~~neither~~ the number of schizonts (Figure 2F) nor phenotypic parameters. DAPT-treated hepatocytes ~~have~~ significant improvements in the number and size of schizonts observed but not in other parasite phenotypic parameters. Improvements were ~~found by~~ SB431542 treatment for all five parameters most notably in number of schizonts and expression of maturation marker, PfMSP1. Similarly, in IWP2-treated hepatocytes showed significant improvements in the sizes of the schizonts, DNA content per schizont and expression of PfMSP1 per schizont. Finally, we tested a previously published cocktail containing all five of these inhibitors (5C) [24] (Figure S3). Interestingly, hepatocyte monolayers cultured in the presence of 5C showed poor support of PfNF175 schizont size and numbers (Figure S4D-J). Together, these data demonstrated that inhibition of specific host pathways, and in particular Wnt, TGF-β, are important for supporting Pf permissiveness and/or schizont development.

Inhibition of the Wnt preserves metabolic profile and epithelial nature of PHHs and improves developmental kinetics of Pf

To study the earliest impact of the Wnt pathway on Pf liver stage development, PHHs were treated with IWP2 (6 days) prior to and during Pf infection (Figure 3A-F). IWP2 treatment promoted parasite development (size, nuclear content and PfMSP1 expression) from day 3 p.i. onwards for all Pf strains. To further capture the transcriptional changes associated with IWP2 treatment, RNA sequencing was performed on hepatocytes cultured in B27 (“mock”) or treated with IWP2 or 5C cocktail at day 5 p.i. The original experiment was performed with five

replicates per culture/treatment condition, but upon closer inspection we decided to exclude replicate 2 from our analysis owing to suboptimal quality (Figure S6A). Based on spatial proximity in the PCA space, B27 cultured cells were very distinct from freshly isolated "fresh" hepatocytes (67% of variance in the PC1 axis, Figure 3G) as also evident from Figure 1F-H. Upon IWP2 treatment we observed a notable shift of B27 cultured samples towards fresh samples on the PCA space. To quantify this drift, we calculated the sample distance scores which indicated increased similarity between IWP2-treated cells and fresh hepatocyte (mean distance score: 168) as compared to B27 cultured cells (mean distance score: 147) (lower scores define higher similarity between samples). Thus, treatment with IWP2 suppressed the transcriptional changes observed during the *in vitro* culture in B27 medium. Interestingly, 5C-treatment elicited additional transcriptional changes apparently not beneficial for Pf development (Figure 3G). In IWP2-treated cells, 394 genes were upregulated and 344 genes downregulated relative to B27 cultured ("mock") cells, (Figure 3I). Gene Ontology analysis of the genes upregulated in IWP2 treated cells revealed enrichment of metabolism ~~associated terms like~~ fatty acid, alcohol and steroid metabolism (Figure 3J). Among the biological processes suppressed upon IWP2 treatment, we found enrichment of extra-cellular matrix organization, surface-adhesion, epithelium migration, response to TGF-β and Wnt-signaling (Figure 3H). Importantly, IWP2 treatment mainly suppressed genes (252 genes) that are otherwise upregulated during in vitro culture in B27 medium (Figure 3K).

We, hence, concluded that IWP2 treatment improves the metabolic profile of cultured hepatocytes while simultaneously suppressing dedifferentiation processes like epithelium to mesenchymal transition, which associates with an improved developmental kinetics of liver schizonts.

Activation status of the Wnt pathway determines the size of Pf schizonts

We subsequently investigated the impact of IWP2 treatment on elements of the Wnt signaling pathway and identified modulation of core signaling components (e.g. suppressed expression of ligand: WNT; receptor: Frizzled; transcription factor: TCF/LEF) (Figure 3L). The most likely effect of the repression of the signaling pathway is the downregulation of associated target genes and consequent alteration of the cellular state. To assess this we investigated the activity of key transcription factors (via quantifying the deregulation of their targets genes). As expected the Wnt-associated transcription factors (e.g. beta-catenin/CTNNB1, TFC7L2) displayed increased activity during *in vitro* culturing and most of their target genes displayed

clear down-regulation upon IWP2 treatment (Figure S6B-D). Next to these we also observed altered activity of the SMAD (TGF-β) and AP1 (noncanonical WNT) transcription factors, which is likely due to indirect effect of WNT deregulation (Figure S6B). This is in agreement with published literature ~~stating the~~ interlinked nature of the two pathways (WNT and TGF-β) [31-33]. However, given the lack of impact of TGF-β inhibitor (SB431542) alone on schizont size (Figure 2G), we are confident of the WNT pathway's impact on parasite development.
To further examine the impact of the Wnt pathway on Pf schizont size, we treated PHHs with the Wnt activator CHIR99021 prior and during PfNF135 and PfNF175 infection (Figure 4A-D). Compared to the B27 control, the number of schizonts were mildly increased for IWP2 treated PHHs but not consistently changed for CHIR99021 (Figure S4A-B). As predicted, schizonts in CHIR99021-treated PHHs were slightly but significantly smaller than B27 cultured schizonts on day 5 p.i. for both Pf strains. There was also a significant size decrease for PfNF135 but not for PfNF175 at day 7 which may be the result of arrested development of PfNF175 under B27 conditions (Figure S3). CHIR99021 treatment also resulted in a relative decrease in PfMSP1 expression significant for PfNF175. Furthermore, pretreatment of hepatocytes with IWP2 or CHIR99021 was followed by return to WBH after NF175 infection, schizont features (i.e. size, DAPI content and PfMSP1 level) were comparable to schizonts observed in hepatocytes cultured in WBH the entire time. Thus, both inhibition and activation of the Wnt pathway in hepatocytes affects predominantly schizont size and maturation. Collectively, it demonstrates that the essential role played by the Wnt signaling pathway of host hepatocytes in determining schizont size and potentially developmental kinetics.

Discussion

In this study, we show that primary human hepatocytes in *in vitro* culture quickly lose their ability to sustain a Pf infection, irrespective of the parasite strain. This loss of permissibility coincides with up-regulation of specific signaling pathways in hepatocytes. Inhibition of individual up-regulated pathways (Wnt, TGF-β, and Notch) results in ~~the~~ enhanced or maintained hepatocyte permissibility ~~and/or~~ improvement in schizont development. In addition, we identify the Wnt-pathway as an important host factor that determines the size and maturity of the developing schizonts.

The combined ~~blockage~~ of four of the up-regulated pathways (Wnt, TGF-β, Notch and BMP) with an adenylate cyclase activator (Forskolin) i.e. the 5C cocktail greatly benefits hepatitis B

virus infection as shown previously [19]. However, this appears detrimental to hepatocytes permissiveness and development of Pf as shown in this study (Figure S4). This could be due to the differing intracellular host requirements by the two distinct pathogens: for example, the intrahepatic growth of HBV is 72 hours compared to the 7 days of Pf. Furthermore, 5C treatment appears to exert transcriptional and/or cellular changes beyond the prevention of EMT (Figure S6). In case of Pf, we show here that specific individual host pathways rather than the combination of all pathways bring strong improvement on parasite development.

We find that the inhibition of the Wnt pathway in cultured hepatocytes have a marked effect on parasite size and maturation. This increase in parasites size is concordant with the upregulation of genes involved in lipid, alcohol and steroid metabolism (Figure 4). While general metabolic health of hepatocyte is conceivably beneficial for parasite growth, fatty acid synthesis in particular is relevant to parasite development and formation of hepatic merozoites [34]. Furthermore, upon Wnt inhibition we find a downregulation of genes typical for EMT transition. In the context of *in vitro* cultures EMT is mainly due to the loss of tissue environment. EMT however can also occur *in vivo* during liver fibrosis [35] and might influence liver stage infection.

While our study highlights the relevance of the inhibition of the Wnt signaling pathway in *in vitro* cultures, this observation might also be relevant during *in vivo* zonal differentiation of hepatocytes. It is well established that within the liver lobule hepatocytes display marked difference in gene expression and metabolism along the portal central axis (also refer to as zonation, [36]) and components of Wnt pathway are predominantly present in zone 3 hepatocytes and heavily involved in maintaining hepatic zonation [37]. We have previously shown that Pf parasites strongly prefer zone 3 (pericentral) hepatocytes resulting in better parasite development [23], as similarly shown for *P. berghei* [38]. While zone 3 hepatocytes possess all components of the Wnt pathway i.e. the surface receptor and intracellular signaling components, the actual external Wnt ligand/signaling proteins may be possibly secreted by hepatocytes from neighbouring zones or other non-hepatocyte cells present in the liver [39] and may therefore also play a role in determining schizont size.

The slight increase in schizont size observed with the other pathway inhibitors may possibly also relate to the Wnt status. Schizonts grown in PHHs treated with forskolin (adenylate cyclase activator) or LDN193189 (inhibitor of Bone Morphogenic Protein pathway) are the relatively

smaller with lower PfMSP1 expression. In forskolin treated schizonts, activated adenylate cyclase leads to increased levels of cAMP which in turn can activate the Wnt pathway[40, 41]. However, the relationship between the BMP pathway (LDN193189) and the Wnt pathway is less obvious as the former can either inhibit or activate the Wnt pathway depending on the presence of wildtype of p53 or the loss of SMAD4 respectively [42]. This may explain the large variation observed in schizonts grown with LDN193189. Inhibition of the Notch pathway (by DAPT) maintains PHHs in their hepatocyte lineage (as opposed to a more bile ductal phenotype) as well as indirect inhibition the Wnt pathway [43]. This could hence explain the observation that, schizonts grown in DAPT-treated PHHs are larger compared to the control B27 medium. Finally, TGF-β signaling leads to the activation of the Wnt pathway [44] therefore its inhibition results in relative better schizonts as for size, nuclear content, and maturation.

Despite their differences in infectivity and phenotype, schizonts of all tested Pf strains are significantly larger and more mature in IWP2-treated PHHs than their counterparts in B27-treated PHHs. This larger size becomes significant during the process of schizont growth i.e. after day 3 p.i. and becomes prominent at day 5 p.i.; blockage of Wnt communication between infected- and uninfected cell could allow for better expansion of the growing schizont with less of host restriction. It has been estimated that an infected hepatocyte can expand up to 200 times its original volume to accommodate the growing Pf schizont [45]. This could necessitate some communication between the infected and uninfected hepatocyte and the Wnt signaling pathway may serve here as communication platform [33,35-37]. IWP2 specifically inhibits the enzyme, porcupine (Porcn), which is involved in transport of the wnt ligands [46]. One could hypothesize that neighbouring hepatocytes keep their strict size and volume via the steady basal level of wnt signaling and thereby limiting the growth of Pf schizonts [47]. A possible mechanism of action is summarised in Figure 5.

In conclusion, we show that PHHs cultured in the standard medium (WBH) rapidly lose their permissiveness to Pf liver stage development. This loss, however, can be substantially delayed by the usage of a defined serum replacement (B27) on top of a William's E Glutamax base. Furthermore, we identify three specific host signaling pathways (Wnt, TGF-β and Notch) to modulate Pf liver stage development. In particular, the Wnt signaling pathway is shown in a novel role to control the size of the developing Pf schizont and is therefore a key factor in

determining the Pf developmental kinetics for multiple parasite strains. Transcriptome analysis of Wnt inhibitor treated hepatocytes indicates that improvement of hepatic metabolism, prevention of EMT and cell division may lead to the positive effects on parasite size and maturation. Together, these findings provide steps forward in understanding the intricate interaction between host and parasite within infected hepatocytes which may benefit rational approaches for future therapeutic interventions.

Materials and Methods

Ethics

Primary human liver cells were freshly isolated from remnants of surgical material. The samples were anonymised and general approval for their use was granted in accordance with the Dutch ethical legislation as described in the Medical Research (Human Subjects) Act. It was confirmed by the Committee on Research involving Human Subjects in the region of Arnhem-Nijmegen, the Netherlands. Approval for use of remnant, anonymized surgical material for transcriptome analysis was specifically confirmed by the Committee on Research involving Human Subjects, in the region of Arnhem-Nijmegen, the Netherlands (CMO-2019-5908).

PHH isolation from liver segments

Primary human hepatocytes were isolated from patients undergoing elective partial hepatectomy as previously described [23, 27]. Freshly isolated hepatocytes are suspended in complete William's B medium (WB): William's E with glutamax (ThermoFisher Scientific: 32551087), 1x Insulin/transferrin/selenium (ThermoFisher Scientific: 41400045), 1mM sodium pyruvate (ThermoFisher Scientific: 11360070), 1x MEM Non-essential amino acid solution (ThermoFisher Scientific: 11140035), 100 units/ml Penicillin-Streptomycin (ThermoFisher Scientific: 15140122) and 1.6μM of dexamethasone (Sigma Aldrich: D4902). PHHs were plated at 62,500 cells per well in 96 well format and kept in a 37°C (5% CO_2) incubator with daily medium changes 96-well plates (Falcon: 353219) precoated with Type 1 collagen solution from rat tail (Sigma Aldrich: C3867).

Generation of sporozoites for liver infection

Pf asexual and sexual stages were cultured in a semi-automatic system as described [48-50]. *Anopheles stephensi* mosquitoes were reared at the Radboud University Medical Center

Insectary (Nijmegen, the Netherlands) in accordance with standard operating procedures. Salivary glands from infected mosquitoes (day 14 to day 23 post infection) were hand-dissected and collected in WB medium. Collected glands were homogenized using home-made glass grinders and sporozoites were counted in a Burker-Turk chamber using phase-contrast microscopes. Immediately prior to infection of human hepatocytes, the sporozoites were supplemented with heat-inactivated human sera (HIHS) at 10% of the total volume (i.e. WBH). The multiplicity of infection is 0.8 (e.g. 50.000 spz to 62500 hepatocytes) or 1 (62500 spz to 62500 hepatocytes) depending on the yield of the sporozoite batches. The MOI is the same within an experiment/biological replicate but may differ between experiments (as it depended on the sporozoite yield of the dissected mosquitoes.

Medium compositions and duration

Different plating time after isolation (Figure 1B, C): Isolated PHH were plated in WB medium. The following day, it was changed to WBH: this medium was refreshed daily until the end of the experiment on day 26 post plating. In figure 1D and E, isolated PHH were again plated in WB medium. The following day, it was changed to William's E with glutamax (ThermoFisher Scientific: 32551087) supplemented with 1x B27 Supplement (ThermoFisher Scientific: 17504044) and 100 units/ml Penicillin-Streptomycin (ThermoFisher Scientific: 15140122) which was referred to as B27 medium or mock in the article. On the day of infection, B27 medium was removed and replaced with sporozoites suspended in WBH for three hours. After the infection process has occurred (i.e. after three hours), the WBH (sporozoite) medium was replaced with B27 medium until the conclusion of the experiment.

Different medium treatments: Isolated PHH were plated in WB medium. The following day, it was changed to the following medium conditions: B27 or B27 supplemented with 20μM of Forskolin (Enzo Life Sciences: BML-CN100-0010), or 10μM of SB431542 (Tocris: 1614), or 0.5μM of IWP2 (Tocris: 3533), or 5μM of DAPT (Tocris: 2634) or 0.1μM of LDN193189 (Tocris: 6053) or the combination of all the compound inhibitors (i.e. 5C). The hepatocytes were kept on this treatment for another 6 days (i.e. 7 days post plating) and then infected with sporozoites where the medium composition changes to WBH for three hours. After three hours, the monolayers were returned to their respective treatments. Media was refreshed on a daily (24hour) basis.

Investigating the Wnt pathway: Isolated PHH were plated in WB medium. The following day, it was changed to the following medium conditions: B27 or B27 supplemented with 0.5μM of IWP2 or 3μM CHIR-99021 (Sigma-Aldrich: SML1046). The hepatocytes were kept on this treatment for another 6 days (i.e. 7 days post plating) and then infected with sporozoites where the medium composition changes to WBH for three hours. After three hours, the monolayers were returned to their respective treatments. Media was refreshed on a daily (24hour) basis.

Immunofluorescence readout

Monolayers were fixed with 4% paraformaldehyde (ThermoFisher Scientific: 28906) for 10 minutes and permeabilised using 1% Triton for 5 minutes. The samples are stained with the various primary Pf or human antibodies: Rabbit PfHSP70 at 1:75 dilution (StressMarq Biosciences: SPC186), Mouse PfMSP1 at 1:100 dilution (Sanaria and NIH/NIAD: AD233), and mouse human glutamine synthethase at 1:100-250 dilution (Abcam: ab64613). Secondary antibodies were used at these following dilutions: Goat anti-rabbit Alexa Fluor 594 at 1:200 dilution (ThermoFisher Scientific: A11012) and goat anti-mouse Alexa Fluor 488 at 1:200 dilution (ThermoFisher Scientific: A11029). DAPI was used at 300 nM to stain the nuclear material of the monolayer.

Microscopy

The Zeiss LSM880 with Airyscan at 63x objectives (oil) and 2x zoom were used for detailed images. For high content images, the Zeiss Axio Observer Inverted Microscope Platform with AI assisted experimental startup was used. The images were acquired at 20x objectives with a numerical aperture of 0.8.

Data analysis using FIJI

Infection rate: For each well, 77 images were acquired in a tiled format. Approximately half (i.e. 39) images were counted on FIJI [51] for NF135 and NF175 infections. All the tiles were counted for NF54 due to the lower infection rate. The number of hepatic nuclei were counted for 1% of the total image (i.e. 7-10 images) and then extrapolated to get number of PHHs per well.

Measurement of schizont size: See Yang et al for further details [23]. Images obtained on the high content microscope were opened in FIJI. Random images were chosen until at least 100

schizonts were measured (per well) unless the infection is with NF54 (at least 50 schizonts). Schizonts were selected using the region of interest (ROI) tool based on PfHSP70 positivity (red channel) and measured. This ROI mask is applied onto the other colour channels i.e. blue and green to obtain values of nuclear content (DAPI) and hGS or PfMSP1 signal. For hGS and PfMSP1, background non-specific staining was considered and subtracted from the final signal. Further details regarding the methods can be found in [21, 23].

RNA Isolation and RNA sequencing library preparation

Freshly isolated or in vitro cultured hepatocytes (mock or treatment) were homogenized in TRIzol solution and cryopreserved at -80°C. RNA was subsequently isolated using the Zymo research Direct zol RNA purification kit (Cat. No. #R2053). The isolated RNA was assessed for quality and quantified using Nanodrop and agarose gel electrophoresis. RNA sequencing libraries from isolated/purified RNA were prepared using the Kapa mRNA Hyperprep Kit (Cat. No. #KK8581) using slight modifications of the manufacturer's instructions for mRNA enrichment method. The input total RNA for the various libraries was selected between 150ng, 250ng or 500ng. The enriched mRNA was fragmented at 94°C for 6min and dA tailing performed at 55°C for 15min. In order to better capture the AT rich *Pf* transcriptome we modified the PCR protocol as follows: Initial denaturation at 98°C for 2min; Cycling denaturation at 98°C for 20sec, annealing + extension at 62°C for 2min; final extension at 62°C for 3min. We selected PCR cycles for library amplification (12, 11 and 10 respectively) based on starting input total RNA amount. The amplified libraries were quantified using Denovix dsDNA high sensitivity kit (Cat. No. #TN145)on a Qubit fluorometer. Further qualitative assessment of library size distribution was performed using the Agilent high sensitivity DNA kit (Cat. No. #5067-4626) on the Agilent 2100 bioanalyzer platform. Each library was sequenced in 42bp paired-end format for roughly 18 million reads on the Illumina Nextseq 500 platform.

RNA sequencing data analysis

The sequencing endline fastq files were quality checked using FASTQC (ver. 0.11.9). The reads were subsequently trimmed for quality (-q 30) and adapter removal using the Trim_Galore software (ver. 0.6.7). The trimmed reads were aligned onto the *Homo sapiens* ver. 38 genome using STAR aligner (ver. 2.7.10a). The reads mapped onto the respective genomes were counted using the –quantMode GeneCounts option in STAR. Reads mapping only to sense strand were corrected for batch effects stemming from different hepatocyte donors using the

Combat-seq tool (sva package ver. 3.44.0). The batch corrected counts were used for subsequent differential expression analysis (for conditions/treatments) in DESEq2 package (ver. 1.36.0) in RStudio (ver. 4.2.2 "Innocent and Trusting"). PCA plot for the various sequenced libraries/samples was generated using plotPCA command. Volcano plots for differentially expressed genes and genes of interest were generated using custom scripts and ggplot2 (ver. 3.4.2) in R. Gene Ontology enrichment analysis was performed in using the clusterProfiler tool (ver. 4.4.4) with subsequent dot-plots generated using inbuilt functions in ggplot2. Heatmaps for differentially expressed genes were generated using the Morpheus online tool by Broad Institute and custom scripts in R (ggplot2). KEGG pathway schematic for Wnt signaling was generated using the pathview tool package (ver. 1.46.0) in R. Transcription factor activity analysis was performed using the decoupleR package (ver. 2.6.0 utilizing collecTRI database) from the Julio Saez-Rodriguez lab [52, 53]. Bar-plot visualization for TF activity scores was done in GraphPad Prism 8.

Statistical analysis

For the majority of the experiments, at least three biological replicates were performed with two technical replicates. All statistical tests were performed using Prism 10. See figure legends for details regarding the statistical tests.

Data availability

The next-generation sequencing dataset associated with this study has been uploaded to Gene Expression Omnibus: GSE263643

The dataset comprises of:
The metadata sheet with information on experiments and samples
Raw data files: fastq files for RNA-sequencing
Processed data files: i) raw counts files generated from Combat-seq and ii) normalized count files generated from DESeq2

Author contributions:

AK, GJG, HW, AS, and ASPY performed the experiments. JHWdW coordinated the collection of the fresh human liver segments. ASPY and AK performed the analysis on the data. TB was involved in the writing and reading of the manuscript. AK, HW, RWS, RB and ASPY were involved in the conceptualization and writing of the manuscript.

Acknowledgements:

We are grateful for R. Stoter, W. Graumans, M. Vegte-Bolmer, A. Pouwelsen, L. Pelser-Posthumus and J. Kuhnen of the Malaria and Insectary Unit at the Radboud University Medical Center for parasite, mosquito and sporozoite production. We would like to thank the Microscopic Imaging Center (MIC) of the Radboud University for access to its facilities. A.S.P.Y was a recipient of the Veni grant from the Dutch Research Council (NWO) talent program (VI.Veni.192.171) and ZonMW Off-Road grant (04510012010050). A.K was supported by the Dutch Research Council (ZonMW-TOP-grant #91218010).

Financial Disclosure:

The salary of A.S.P.Y was supported by the Dutch Research Council (NWO) talent program (VI.Veni.192.171) and ZonMW Off-Road grant. A.K. was supported by the Dutch Research Council (ZonMW-TOP-grant #91218010). However, the funders had no role in study design, data collection, analysis, decision to publish or preparation of the manuscript.

References:

1. WHO, W.H.O., *World Malaria Report 2022*. 2022.
2. Vaughan, A.M., et al., *Complete Plasmodium falciparum liver-stage development in liver-chimeric mice.* J Clin Invest, 2012. **122**(10): p. 3618-28.
3. Mo, A.X. and G. McGugan, *Understanding the Liver-Stage Biology of Malaria Parasites: Insights to Enable and Accelerate the Development of a Highly Efficacious Vaccine.* Am J Trop Med Hyg, 2018. **99**(4): p. 827-832.
4. Langlois, A.C., et al., *Plasmodium sporozoites can invade hepatocytic cells independently of the Ephrin receptor A2.* PLoS One, 2018. **13**(7): p. e0200032.
5. Silvie, O., et al., *Expression of human CD81 differently affects host cell susceptibility to malaria sporozoites depending on the Plasmodium species.* Cell Microbiol, 2006. **8**(7): p. 1134-46.
6. Silvie, O., et al., *Hepatocyte CD81 is required for Plasmodium falciparum and Plasmodium yoelii sporozoite infectivity.* Nat Med, 2003. **9**(1): p. 93-6.
7. Mancio-Silva, L., et al., *Nutrient sensing modulates malaria parasite virulence.* Nature, 2017. **547**(7662): p. 213-216.
8. Lahree, A., J. Mello-Vieira, and M.M. Mota, *The nutrient games - Plasmodium metabolism during hepatic development.* Trends Parasitol, 2023. **39**(6): p. 445-460.
9. Posfai, D., et al., *Plasmodium parasite exploits host aquaporin-3 during liver stage malaria infection.* PLoS Pathog, 2018. **14**(5): p. e1007057.
10. Rodrigues, C.D., et al., *Host scavenger receptor SR-BI plays a dual role in the establishment of malaria parasite liver infection.* Cell Host Microbe, 2008. **4**(3): p. 271-82.
11. Real, E., et al., *Plasmodium UIS3 sequesters host LC3 to avoid elimination by autophagy in hepatocytes.* Nat Microbiol, 2018. **3**(1): p. 17-25.
12. Thieleke-Matos, C., et al., *Host cell autophagy contributes to Plasmodium liver development.* Cell Microbiol, 2016. **18**(3): p. 437-50.
13. van de Sand, C., et al., *The liver stage of Plasmodium berghei inhibits host cell apoptosis.* Mol Microbiol, 2005. **58**(3): p. 731-42.
14. Ebert, G., et al., *Targeting the Extrinsic Pathway of Hepatocyte Apoptosis Promotes Clearance of Plasmodium Liver Infection.* Cell Rep, 2020. **30**(13): p. 4343-4354 e4.
15. Soulard, V., et al., *Plasmodium falciparum full life cycle and Plasmodium ovale liver stages in humanized mice.* Nat Commun, 2015. **6**: p. 7690.
16. Sacci, J.B., Jr., et al., *Plasmodium falciparum infection and exoerythrocytic development in mice with chimeric human livers.* Int J Parasitol, 2006. **36**(3): p. 353-60.
17. Sattabongkot, J., et al., *Establishment of a human hepatocyte line that supports in vitro development of the exo-erythrocytic stages of the malaria parasites Plasmodium falciparum and P. vivax.* Am J Trop Med Hyg, 2006. **74**(5): p. 708-15.
18. Tweedell, R.E., et al., *The Selection of a Hepatocyte Cell Line Susceptible to Plasmodium falciparum Sporozoite Invasion That Is Associated With Expression of Glypican-3.* Front Microbiol, 2019. **10**: p. 127.
19. Kaushansky, A., et al., *Malaria parasites target the hepatocyte receptor EphA2 for successful host infection.* Science, 2015. **350**(6264): p. 1089-92.
20. Roth, A., et al., *A comprehensive model for assessment of liver stage therapies targeting Plasmodium vivax and Plasmodium falciparum.* Nat Commun, 2018. **9**(1): p. 1837.

21. Yang, A.S.P., et al., *Development of Plasmodium falciparum liver-stages in hepatocytes derived from human fetal liver organoid cultures.* Nat Commun, 2023. **14**(1): p. 4631.
22. Yalaoui, S., et al., *Scavenger receptor BI boosts hepatocyte permissiveness to Plasmodium infection.* Cell Host Microbe, 2008. **4**(3): p. 283-92.
23. Yang, A.S.P., et al., *Zonal human hepatocytes are differentially permissive to Plasmodium falciparum malaria parasites.* EMBO J, 2021. **40**(6): p. e106583.
24. Xiang, C., et al., *Long-term functional maintenance of primary human hepatocytes in vitro.* Science, 2019. **364**(6438): p. 399-402.
25. Gomez-Lechon, M.J., et al., *Competency of different cell models to predict human hepatotoxic drugs.* Expert Opin Drug Metab Toxicol, 2014. **10**(11): p. 1553-68.
26. Mazier, D., et al., *Complete development of hepatic stages of Plasmodium falciparum in vitro.* Science, 1985. **227**(4685): p. 440-2.
27. Walk, J., et al., *Modest heterologous protection after Plasmodium falciparum sporozoite immunization: a double-blind randomized controlled clinical trial.* BMC Med, 2017. **15**(1): p. 168.
28. Mallanna, S.K. and S.A. Duncan, *Differentiation of hepatocytes from pluripotent stem cells.* Curr Protoc Stem Cell Biol, 2013. **26**: p. 1G 4 1-1G 4 13.
29. Huch, M., et al., *Long-term culture of genome-stable bipotent stem cells from adult human liver.* Cell, 2015. **160**(1-2): p. 299-312.
30. Hu, H., et al., *Long-Term Expansion of Functional Mouse and Human Hepatocytes as 3D Organoids.* Cell, 2018. **175**(6): p. 1591-1606 e19.
31. Guo, X. and X.-F. Wang, *Signaling cross-talk between TGF-β/BMP and other pathways.* Cell research, 2009. **19**(1): p. 71-88.
32. Luo, K., *Signaling cross talk between TGF-β/Smad and other signaling pathways.* Cold Spring Harbor perspectives in biology, 2017. **9**(1): p. a022137.
33. Guo, X., et al., *Axin and GSK3-β control Smad3 protein stability and modulate TGF-β signaling.* Genes & development, 2008. **22**(1): p. 106-120.
34. van Schaijk, B.C., et al., *Type II fatty acid biosynthesis is essential for Plasmodium falciparum sporozoite development in the midgut of Anopheles mosquitoes.* Eukaryot Cell, 2014. **13**(5): p. 550-9.
35. Fabris, L., et al., *Revisiting Epithelial-to-Mesenchymal Transition in Liver Fibrosis: Clues for a Better Understanding of the "Reactive" Biliary Epithelial Phenotype.* Stem Cells Int, 2016. **2016**: p. 2953727.
36. Gebhardt, R., *Metabolic zonation of the liver: regulation and implications for liver function.* Pharmacol Ther, 1992. **53**(3): p. 275-354.
37. Russell, J.O. and S.P. Monga, *Wnt/beta-Catenin Signaling in Liver Development, Homeostasis, and Pathobiology.* Annu Rev Pathol, 2018. **13**: p. 351-378.
38. Afriat, A., et al., *A spatiotemporally resolved single-cell atlas of the Plasmodium liver stage.* Nature, 2022. **611**(7936): p. 563-569.
39. Zeng, G., et al., *Wnt'er in liver: Expression of Wnt and frizzled genes in mouse.* Hepatology, 2007. **45**(1): p. 195-204.
40. Suzuki, A., et al., *PTH/cAMP/PKA signaling facilitates canonical Wnt signaling via inactivation of glycogen synthase kinase-3beta in osteoblastic Saos-2 cells.* J Cell Biochem, 2008. **104**(1): p. 304-17.
41. Guo, R., et al., *Targeting Adenylate Cyclase Family: New Concept of Targeted Cancer Therapy.* Front Oncol, 2022. **12**: p. 829212.

42. Voorneveld, P.W., et al., *The BMP pathway either enhances or inhibits the Wnt pathway depending on the SMAD4 and p53 status in CRC.* Br J Cancer, 2015. **112**(1): p. 122-30.
43. Geisler, F. and M. Strazzabosco, *Emerging roles of Notch signaling in liver disease.* Hepatology, 2015. **61**(1): p. 382-92.
44. Bogaerts, E., et al., *The roles of transforming growth factor-beta, Wnt, Notch and hypoxia on liver progenitor cells in primary liver tumours (Review).* Int J Oncol, 2014. **44**(4): p. 1015-22.
45. Vaughan, A.M. and S.H.I. Kappe, *Malaria Parasite Liver Infection and Exoerythrocytic Biology.* Cold Spring Harb Perspect Med, 2017. **7**(6).
46. Wang, X., et al., *The development of highly potent inhibitors for porcupine.* J Med Chem, 2013. **56**(6): p. 2700-4.
47. Acebron, S.P., et al., *Mitotic wnt signaling promotes protein stabilization and regulates cell size.* Mol Cell, 2014. **54**(4): p. 663-74.
48. Ifediba, T. and J.P. Vanderberg, *Complete in vitro maturation of Plasmodium falciparum gametocytes.* Nature, 1981. **294**(5839): p. 364-6.
49. Ponnudurai, T., et al., *Cultivation of fertile Plasmodium falciparum gametocytes in semi-automated systems. 1. Static cultures.* Trans R Soc Trop Med Hyg, 1982. **76**(6): p. 812-8.
50. Ponnudurai, T., et al., *Infectivity of cultured Plasmodium falciparum gametocytes to mosquitoes.* Parasitology, 1989. **98 Pt 2**: p. 165-73.
51. Schindelin, J., et al., *Fiji: an open-source platform for biological-image analysis.* Nat Methods, 2012. **9**(7): p. 676-82.
52. Müller-Dott, S., et al., *Expanding the coverage of regulons from high-confidence prior knowledge for accurate estimation of transcription factor activities.* Nucleic Acids Research, 2023. **51**(20): p. 10934-10949.
53. Badia-i-Mompel, P., et al., *decoupleR: ensemble of computational methods to infer biological activities from omics data.* Bioinformatics advances, 2022. **2**(1): p. vbac016.

Figure 1: *Pf*-schizont development post-hepatocyte plating and transcriptomic profiles of primary human hepatocytes (PHH). A) The solid arrows show the number of days after liver isolation (post-plating) and the lines with the clear triangles show the period of schizont development. B) The number of schizonts in PHH on day 5 post infection (p.i.) when infected on different days p.p. for parasite strains: NF175 (magenta;), NF135 (green;) and NF54 (blue) in two independent experiments (except NF175). C) The number of PHHs present on day 5 p.i. on indicated days p.p for the specific parasite strains. For each independent experiment, the mean of three replicates is shown along with the standard deviation. The number of schizonts (D) and hepatocytes (E) on day 5 post invasion (p.i.) for NF135 and NF54 (n=1) for different infection times post plating (p.p.) cultured in B27 medium (grey). Dot-plots depicting the biological processes downregulated (F) and upregulated (H) in cultured hepatocytes as compared to freshly-isolated hepatocytes. (G) Volcano plot highlighting the differentially

expressed genes between hepatocytes cultured in B27 medium ("mock") and freshly isolated hepatocytes ("fresh").

Figure 2: Effect of signaling pathways on Pf schizont development. A) Dot-plot depicting the signaling pathways exhibiting higher activity in *in vitro* cultured hepatocytes as compared to freshly isolated hepatocytes. Heatmaps representing normalized expression of genes from (B)Wnt, (C)TGFβ, (D)Notch, (E)BMP and Adenylate cyclase signaling pathways deregulated in cultured vs freshly isolated hepatocytes. The data is scaled and representative of 2 biological replicates of RNA sequencing. The effect of specific host pathway inhibitors (IWP2, SB431542, DAPT, LDN193189 and Forskolin) on respectively schizont numbers (F; each dot is the average count of two technical replicates), size of schizonts (G; each dot is the median of at least 100 schizonts), total DAPI per schizont (H; each dot is the median of at least 100 schizonts), and total PfMSP1 per schizont (I; each dot is the median of at least 100 schizonts) on day 5 p.i.. The grey dotted line (per graphs F-I) shows the median measurements of schizonts grown in B27 control (from four independent experiments). The p-values from a Dunnett's multiple comparisons test are shown – see supplementary information for full p values. J) Representative confocal images (from four independent experiments) showing schizonts grown in B27 alone and B27 supplemented with Forskolin, SB431542, IWP2, DAPT and LDN193189 on day 5 p.i. stained with PfMSP1, PfHSP70 and DAPI. Scale bar is 25 microns.

Figure 3: Effect of IWP2 on developmental kinetics of NF175 (A-B). NF135 (C-D) and NF54 (E-F) schizonts. For each Pf strain, the number schizonts, the size of schizonts, the total DAPI content and total PfMSP1 content are shown from left to right. The schizonts are either grown in hepatocytes cultured in either B27 (grey) or B27 supplemented with IWP2 (orange) for 6 days prior to infection and also during schizont development on days 3, 5 and 7 p.i.. Note briefly that day 3 schizonts of NF175 was not examined. For the number of schizonts, the mean of three independent experiments, each with 2-3 internal replicates is shown. For the schizont size and the total DAPI per schizont, at least 100 schizonts per each of three independent experiments (with two replicates per experiment) were measured. The mean of the median from each experiment is plotted and the graph shows the mean with standard deviation. For the total MSP1 content, at least 100 schizonts per each of three independent experiments were measured. The median is plotted and the graph shows the mean with standard deviation. A Dunnett's multiple comparisons test is performed: see supplementary information for full p values. B, D, F) Representative confocal images (from three independent experiments)

showing NF175, NF135 and NF54 schizonts grown in B27 alone and B27 supplemented with IWP2 on day 7 p.i. stained with MSP1, HSP70 and DAPI respectively. Scale bar is 25 microns. (G) PCA plot highlighting the unique transcriptional states of freshly isolated ("fresh"), cultured ("mock"), IWP2 or 5C-treated hepatocytes. Dot-plots depicting the biological processes downregulated (H) or upregulated (J) in IWP2-treated as compared to "mock" cultured hepatocytes. I) Volcano plot highlighting differential gene expression between IWP2-treatmentand "mock"-cultured hepatocytes. K) Gene expression heatmap highlighting the genes upregulated in mock cultured hepatocytes and suppressed upon IWP2 treatment. The expression counts have been normalized and scaled for plotting. L) Schematic overview of key genes involved in the Wnt signaling pathway that are modulated by IWP2 treatment. The Fold-change (log2) for the genes are scaled and plotted in blue-red color schematic.

Figure 4: The impact of the Wnt signaling pathway on schizont size. Hepatocytes treated with B27 or B27 supplemented with IWP2/CHIR99021 and infected with either NF175 (A) or NF135 (B). Schizont size (left), and total DAPI (middle) content are measured on days 5 and 7 post infection. The total amount of PfMSP1 per schizont was measured on day 7 p.i. (right). Each dot represents a schizont with at least 100 schizonts measured for three independent experiments. The median is shown, and a Dunn's multiple comparisons test is performed with the p values displayed: see supplementary information for full p values. Representative confocal images (from three independent experiments) showing NF175 (C) and NF135 (D) schizonts B27 or B27 supplemented with IWP2/CHIR99021 on day 7 p.i. stained with MSP1, HSP70 and DAPI. Scale bar is 25 microns.

Figure 5: Schematic of a possible mechanism in which the Wnt pathway controls Pf schizont development. A) Under untreated and uninfected condition, there is a steady level of beta-catenin (β-catenin). In addition to its role in gene transcription, β-catenin is the intracellular component that links the cadherin adhesion molecules to actin filaments and therefore controls the "rigidity" of the cells in relation to its neighbours. B) In an infected hepatocyte (after 3 days post infection), the growing Pf schizont takes up so much of the hepatocyte volume that the trafficking of the Wnt ligands to the surface is disrupted. Wnt receptor of the neighbouring uninfected cells are not activated, and β-catenin molecules are degraded due to the phosphorylation of the enzyme, glycogen synthase kinase 3 (GSK3): this allows some flexibility in the uninfected neighbouring cells to accommodate the growth of the infected cell. However in these (uninfected cells), Wnt ligands are still present and can interact

with the existing Wnt receptors on the infected hepatocytes to maintain some β-catenin in the infected cell, thus limiting the growth/size of the schizont. C) Under IWP2 treatment, Wnt ligands are not trafficked to the surface of both uninfected and infected hepatocytes due to the inhibition of the enzyme porcupine (target of IWP2). Porcupine "labels" (via palmitoylation) Wnt ligands for correct trafficking to the plasma membrane. As a result, Wnt receptors on both uninfected and infected cells are not activated and existing β-catenin are degraded leading to reduced connection between cadherin and actin filaments (i.e. cell-cell contacts) and ultimately reducing rigidity. D) Under CHIR99021 (GSK3 inhibitor) treatment, β-catenin in both uninfected and infected cells cannot be phosphorylated nor degraded. Both cell types are more rigid due to the improve connection between actin filaments and cadherin which severly limits the size of the Pf schizont.

Supplementary Figures

Figure S1: The impact of heat inactivated human sera on Pf schizont development. PHH monolayers were cultured in either William's B (WB) or William's B supplemented with 10% heat inactivated sera (WBH) for six days p.p. and infected with either NF175 (A) or NF135 (B) or NF54 (C). Data are shown for respectively schizont number, size, total DAPI content, percentage of human glutamine synthetase (hGS) positive schizonts, total hGS level per schizont and total merozoite surface protein 1 (MSP1) level per schizont. As for the number of schizonts, each dot represents an independent experiment showing the mean of two replicates. As for the size and total DAPI content graphs, each dot represents an independent experiment showing the mean of the median of at least 100 schizonts per replicate (2 replicates) were measured (except in NF54 condition where there is not enough schizonts present). For the percentage of hGS positive schizonts, each dot represents an independent experiment where at least 100 schizonts were examined from one replicate. For the total hGS level and total MSP1 level, each dot represents an independent experiment of the median from at least 100 schizonts (except in NF54 condition where there is not enough schizonts present). The error bars show the mean with the standard deviation. The p-values are generated by performing an unpaired t-test between WBH and WB and can be found in the supplementary information excel sheet (*** = 0.0001).

Figure S2: Effect of specific inhibitors of PHH gene-signaling pathways on the expression Pf schizont markers A) Schematic experimental setup of Figure 3 and S3. PHHs were treated for 6 days p.p with either B27 or B27 supplemented

IWP2/SB431542/Forskolin/DAPT/LDN193189. The experiment was analysed at day 5 p.i.. with NF175 B) Total number of hepatocytes per well with each dot representing the average three wells of one biological replicate. C)Total hGS per schizont with each dot representing the median of at least 100 schizonts. The percentage of hGS (D) and MSP1 (E) positive schizonts: each dot is an independent experiment where at least 100 schizont is measured. The grey dotted line (per graphs C-E) shows the median measurements of schizonts grown in B27 (from four independent experiments) as each host pathway inhibitor treatment was made in media containing B27. The mean and standard deviation is shown for each graph. The p-values from a Dunnett's multiple comparisons test are displayed. F) Representative confocal images (from four independent biological experiments) showing schizonts grown in B27 alone and B27 supplemented with IWP2, SB431542, DAPT, LDN193189, Forskolin stained with hGS, HSP70 and DAPI. Scale bar is 25 microns.

Figure S3: The effect of B27 or B27 supplemented with 5C on *Pf*-permissiveness and schizont development in PHH. The number of schizonts (A) and hepatocytes (B) on day 5 p.i. for NF135 and NF54 (n=1) at indicated time points p.p. when cultured in either B27 medium (grey) or B27 supplemented with 5C (blue). Each dot shows the mean of three replicates and the error bars show the standard deviation. C) Experimental setup of graphs D-L. PHHs were cultured for 6 days p.p. with either B27 or B27 with 5C and infected with NF175 parasites and analysed at day 5 p.i.. D) Total number of hepatocytes per well with each dot representing the average three wells of one biological replicate. E)The number of schizonts per well was normalised to the number of schizonts counted in WBH (red dotted line). Each dot is the mean of an independent experiment with two replicates. For the schizont size (F) and total DAPI content per schizont (G), each dot represents the mean of an independent experiment of at least 100 schizonts. For the total hGS (H) and MSP1 (I) per schizont, each dot represents the median of an independent experiment of at least 100 schizont For the percentage of hGS (J) and MSP1 (K) positive cells, each dot represents an independent experiment of at least 100 schizonts. D-K shows the mean with the standard deviation). Representative confocal images (from four independent experiments) showing schizonts grown in WBH or B27 alone or B27 supplemented with 5C on day 5 p.i. stained with hGS (L) or MSP1 (M), HSP70 and DAPI. Scale bar is 25 microns.

Figure S4: The impact of the Wnt signaling pathway on numbers of developing schizonts. A) The number of schizonts in NF135 infected PHHs treated with B27 or B27 supplemented

with IWP2/CHIR99021. Each graph shows an independent experiment, each with duplicates per condition. B) Number of schizonts (left) and number of hepatocytes (right) in NF175-infected PHHs treated with B27 or B27 supplemented with IWP2/CHIR99021. Each graph shows the mean and the standard deviation of three independent experiments: each experiments have duplicate wells. There was no statistic difference between conditions within the same day post infection. Representative confocal images (from three independent biological experiments) showing NF135 (C) and NF175 (D) schizonts B27 or B27 supplemented with IWP2/CHIR99021 on day 5 p.i. stained with hGS, HSP70 and DAPI. Scale bar is 25 microns.

Figure S5: The impact of Wnt signaling on determining NF175 development in the liver A) schematic showing the media treatment timing and conditions. Hepatocytes are treated with B27 or B27 supplemented with IWP2/CHIR99021. After exposure to NF175, cultures were either kept in B27 or B27 supplemented with IWP2/CHIR99021 (closed circles) or returned to WBH (IWP2* and CHIR99021* - open circles). The number of schizonts (B), size of schizonts (C), total nuclear content per schizont (D) and total MSP1 per schizont (E) on day 5 post infection. The number of schizonts (F), size of schizonts (G), total nuclear content per schizont (H) and total MSP1 per schizont (I) on day 7 post infection. Each dot represents an independent biological donor where the median of at least 100 schizonts were measured. Dunnett's multiple comparison test (to WBH) were performed for each graph and the statistical significance is indicated: see supplemental information excel sheet for the full p values.

Figure S6: IWP2 treatment modulates expression of target genes downstream of Wnt responsive transcription factors. A) PCA plot of all replicates of freshly-isolated, "mock-cultured", IWP2 or 5C-treated hepatocytes. B) Bar-plot depicting the scaled activity scores of key Wnt, TGFβ and non-canonical Wnt transcription factors across freshly-isolated hepatocytes, "mock-" and IWP2-treated hepatocytes. C-D) Volcano plots depicting a repressive effect of IWP2 treatment on the target genes underlying key Wnt signaling transcription factors CTNNB1 (β-catenin; C) and TCF7L2 (D). Genes that are positively modulated by these TFs and suppressed upon IWP2 treatment are highlighted in blue.

Supplementary information on statistical tests

Each tab of the excel sheet presents a table of the statistical tests performed per figure with the associated P-values.

Figure 1 Click here to access/download;Figure;Figure1_new.tif

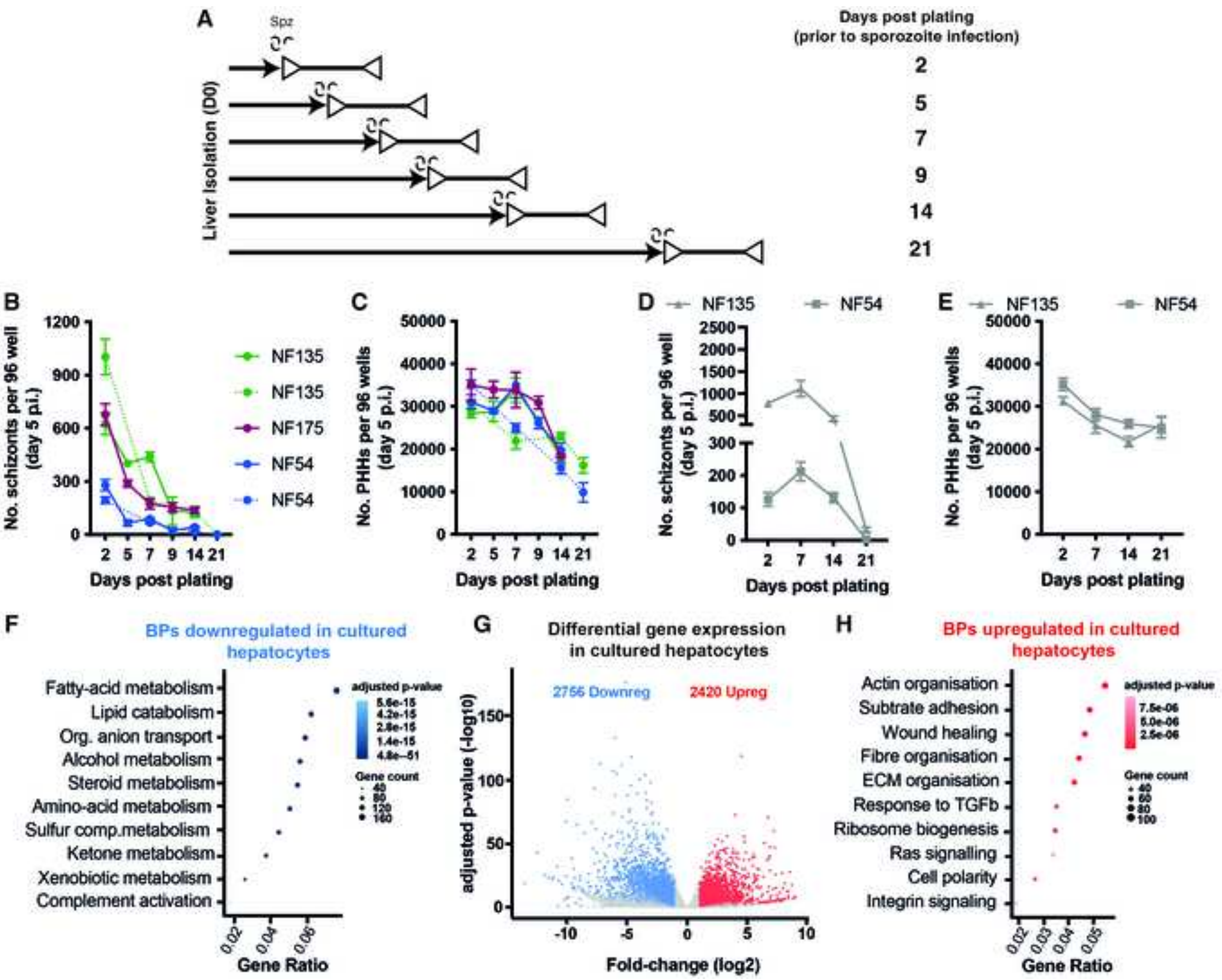

Figure 2

Click here to access/download;Figure;Figure2_NAAS.tif

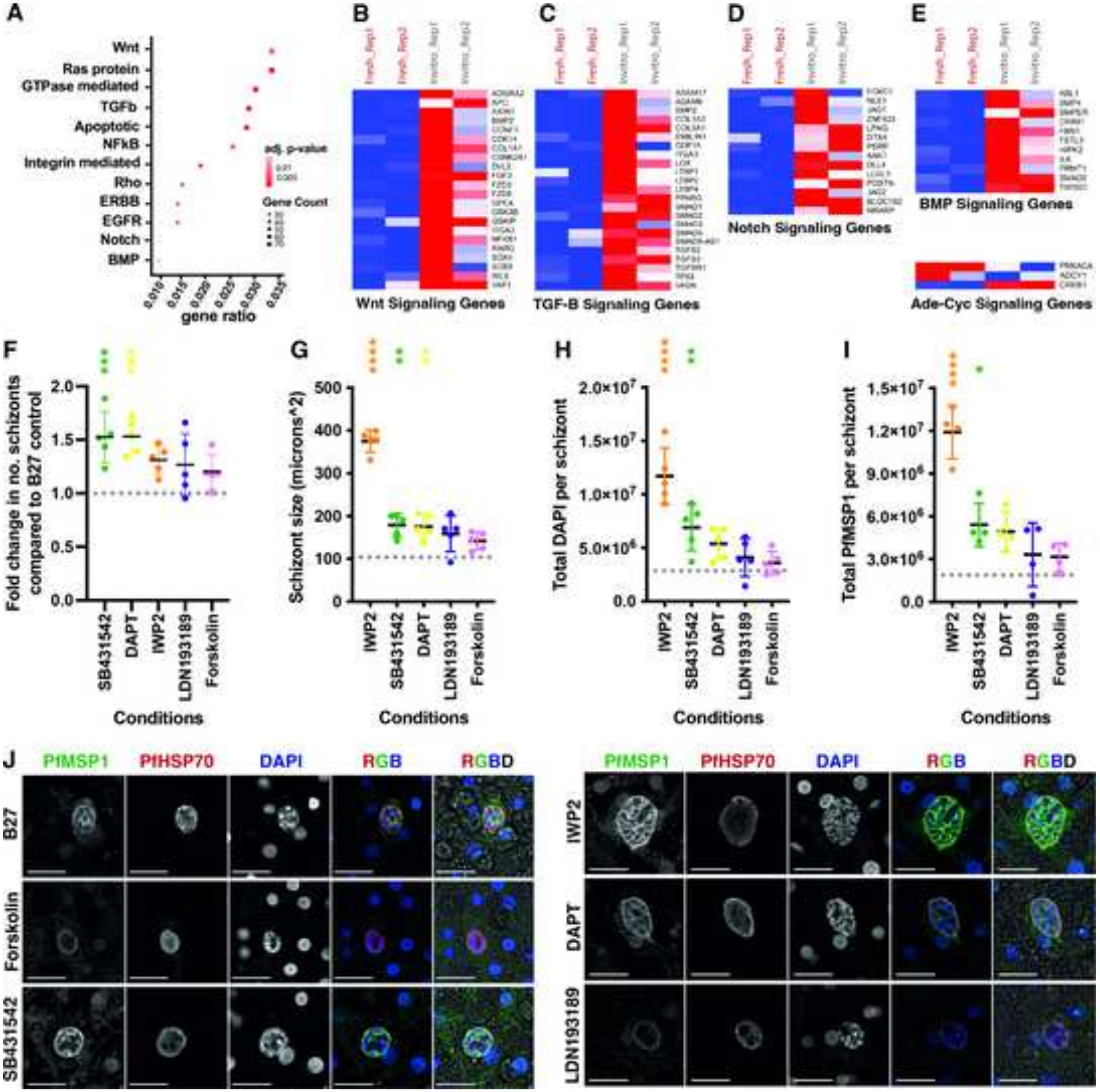

Figure 3 Click here to access/download;Figure;Figure3_NAAS.tif

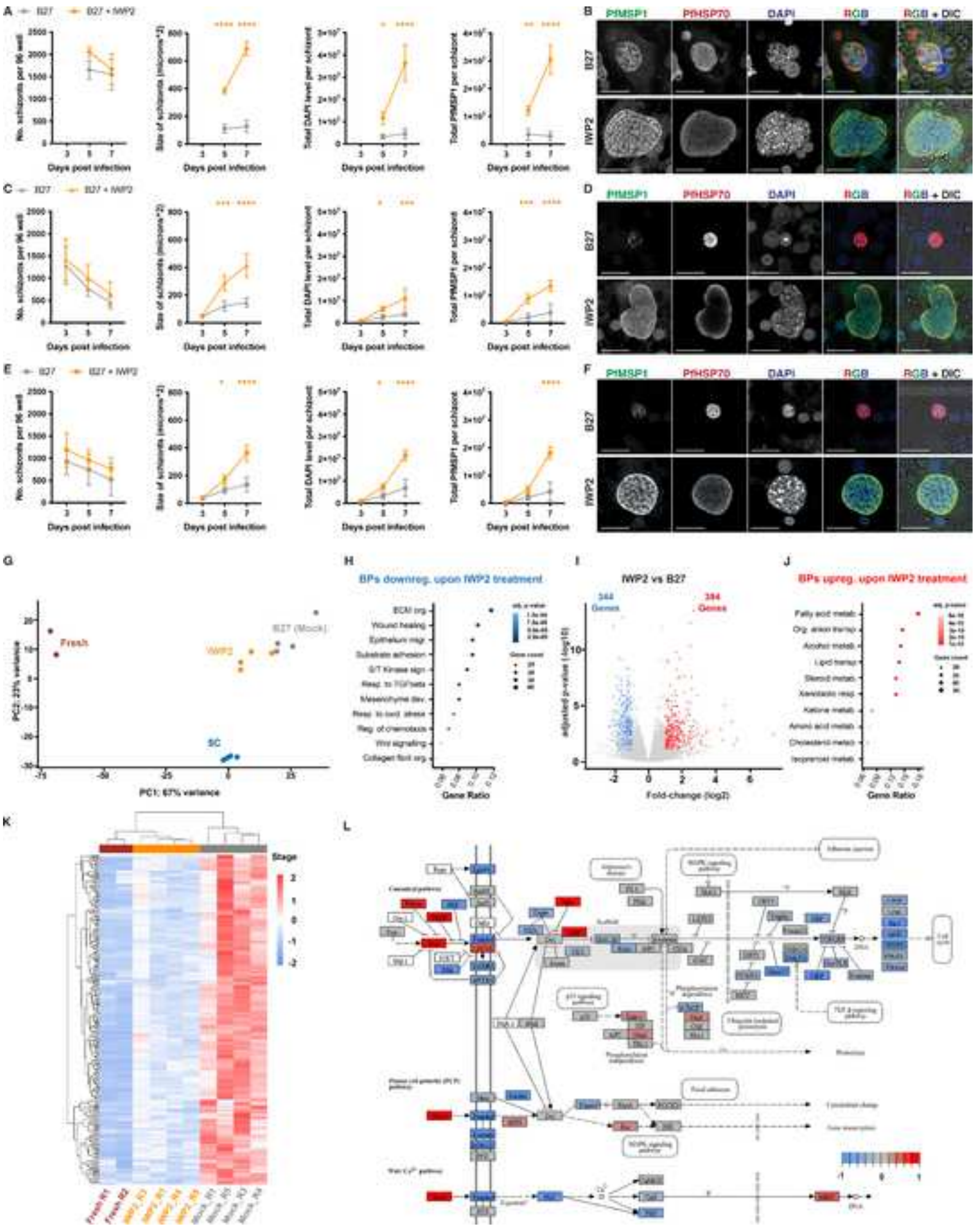

Figure 4

Click here to access/download;Figure;Figure4_NAAS.tif

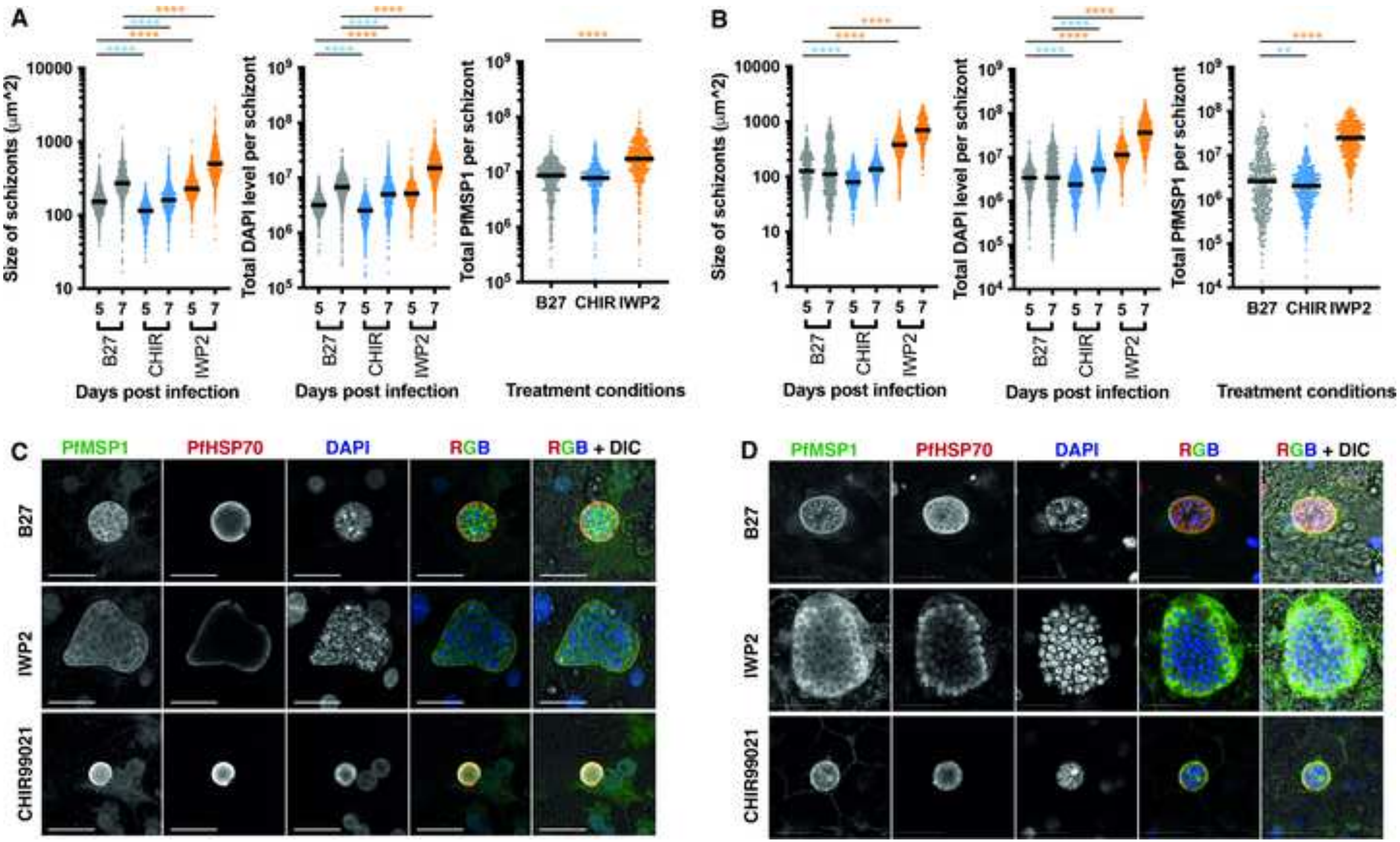

Click here to access/download;Figure;Figure5.tif

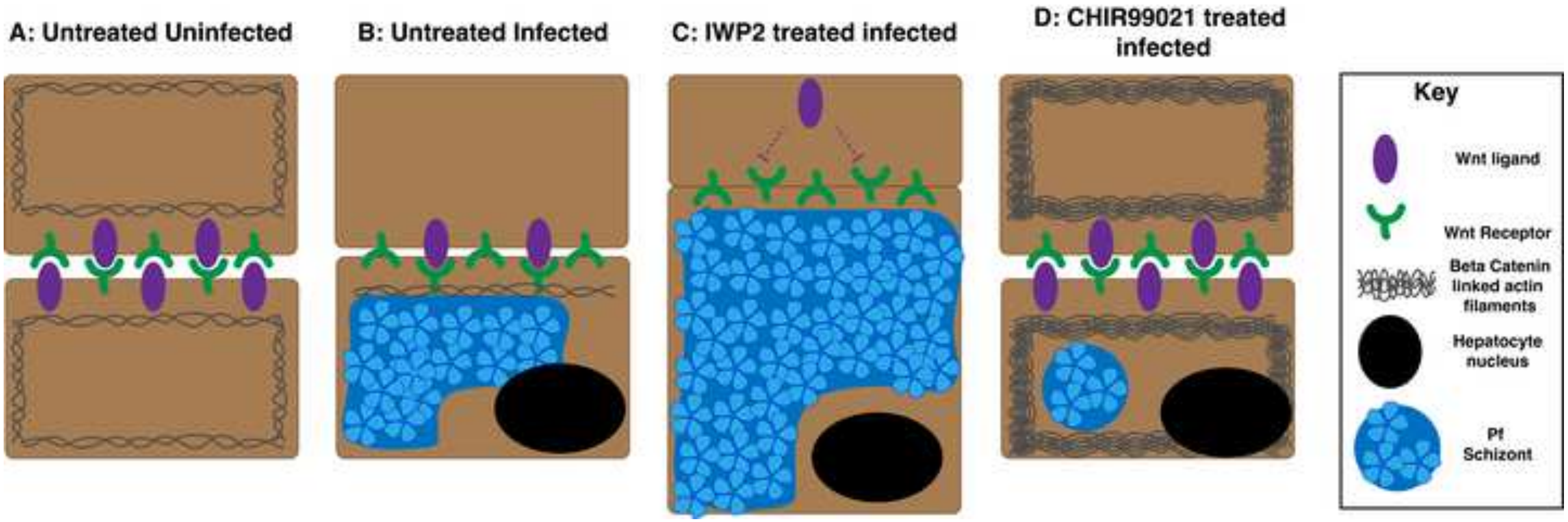

Click here to access/download
Supporting Information
Sup info stats test.xlsx

Click here to access/download
Supporting Information
Supplementary Figure.docx

Modulation of host signa~~l~~ling pathways reveal a major role for Wnt signa~~l~~ling in the maturation of *Plasmodium falciparum* liver schizonts

Abhishek Kanyal[1]*, Geert-Jan van Gemert[2]*, Haoyu Wu[1,3]*, Alex van der Starre[2], Johannes HW de Wilt[4], Teun Bousema[2], Robert W. Sauerwein[2,], Richard Bartfai[1]*, Annie SP Yang[2,5,6]*

*these authors contributed equally

1. Department of Molecular Biology, Faculty of Science, Radboud Institute for Molecular Life Science, Radboud University, Nijmegen, The Netherlands

2. Radboud Center of Infectious Diseases, Department of Medical Microbiology, Radboud University Medical Center, Nijmegen, the Netherlands

3. CAS Key Laboratory of Regenerative Biology, Guangzhou Institutes of Biomedicine and Health, Chinese Academy of Sciences, Guangzhou 510530, China

4. Department of Surgery, Radboud University Medical Center, Nijmegen, the Netherlands

5. Present address: ~~Department of Parasitology,~~ Leiden University Center for Infectious Diseases (LUCID), Leiden University Medical Center, Leiden, the Netherlands

6. Lead contact.

Correspondence: a.s.yang@lumc.nl (ASPY) or r.bartfai@science.ru.nl (RB)

Abstract:

After infection of the human host, the initial stage of the *Plasmodium falciparum* (Pf) lifecycle takes place in the liver. However, understanding of the host-parasite interaction has been limited by the rapid loss of functionality in cultured primary human hepatocytes (PHHs). Here, we link loss of hepatic functionality to drastic loss in Pf permissiveness, which we effectively prevent by using a novel medium containing serum-replacement and signal transduction inhibitors. Integrating transcriptomic analysis and phenotypic assessment of infection outcome, we identified several host signa~~l~~ling pathways that influence Pf liver stage development. Inhibition of the Wnt pathway in particular plays a major role in determining the size and maturity of Pf-liver schizonts, via retaining metabolic activity and epithelial nature of hepatocytes. Host signa~~l~~ling pathways determining Pf liver stage permissiveness provide insight into the complex host-parasite interaction and may accelerate development of novel therapeutic strategies for Pf-liver stages. ~~(145)~~

Formatted: English (United Kingdom)

Formatted: Font color: Text 1

Authors' summary

Despite substantial elimination and control measures malaria still causes substantial mortality and global disease burden. Liver stage development of the malaria parasite is a critical first step preceding clinical symptoms and is the prime target of antimalarial vaccine/drug development. We, however, lack sufficient understanding of critical components of the host liver environment supporting or preventing parasite development. In this study, we observed substantial decline in the ability of cultured human hepatocytes to support parasite development and identified molecular signatures and altered activity of host signalling pathways associated with the loss of permissiveness. We, in particular, demonstrate the substantial impact of the Wnt pathway on the growth and maturation of liver-stage parasites. We hence provide insight into how host tissue homeostasis can impact on parasite development and pave the way for further dissection of host pathogen interaction and development of improved intervention strategies.

Introduction

The mosquito-borne disease, malaria, remains a devastating global burden with approximately 250 million cases and 620,000 deaths annually [1]. Most of the deaths are due to the parasite *Plasmodium falciparum* (Pf), which begins its developmental life-cycle inside liver cells (hepatocytes) after initial deposition into the skin by an infected mosquito. Over a period of approximately one week, a small number of these parasites (sporozoites) will replicate and differentiate into intracellular schizont, each containing approximately 90000 blood-infective daughter merozoites [2]. The release of these merozoites into the circulation and their subsequent cyclical asexual replication in red blood cells (RBCs) is responsible for clinical symptoms associated with the disease.

Despite its fundamental importance in enabling effective infection, currently fundamental understanding of the complex parasite-host interaction within the infected hepatocytes remains elusive. Studies with the rodent *Plasmodium berghei* (Pb) model have been informative as a proxy for Pf but findings are often not translatable to Pf-host interactions as in particular the rodent parasite has only a 2-day liver-stage period compared to 7-days for Pf [3]. Specific host membrane receptors have been identified to be involved in Pb sporozoite invasion of hepatocytes such as CD81 and Scavenger Receptor B1 (SR-B1) [4-6]; similarly, host factors involved in nutrient acquisition [7-10] and/or prevention of host cell death pathways [11-14].

Research in Pf primarily involves *in vivo* models such as mice with humanised liver [2, 15, 16] or *in vitro* models where hepatocyte cell-lines such as HC-04 [17, 18] and primary human hepatocytes (PHHs) are used. The usage of cell-lines has been limited to the identification of host receptors involved in parasite invasion such as EphA2 [4, 19] and glypican 3 [18]. Contrastingly, PHHs are considered the gold standard in drug assays [3, 20] and used to identify host factors needed for the development of Pf liver schizont such as SR-B1 [10, 21, 22] and glutamine synthetase [23]. A well-known phenomenon of *in vitro* cultured PHHs, however, is the quick loss of hepatic features [24, 25], yet its impact has not been explored in Pf liver stages and may hinder the discovery of host factors that are important for full maturation of liver schizonts. Establishment of an *in vitro* hepatocyte model that preserve hepatocyte features ~~to allow for late-stage Pf maturation~~ is therefore a prerogative and the next logical frontier in research. The loss of functional hepatic features is controlled by specific host signa~~l~~ling pathways [24] of which, their potential impact on parasite development have not been previously examined.

Here, we set out to 1) identify the impact of loss of hepatic functions in *in vitro* hepatocytes and their permissiveness to Pf infection, 2) improve and stabilize hepatocyte culture conditions for Pf permissiveness, facilitating complete schizont development and 3) identify host pathways in hepatocytes that influence the developmental kinetics of Pf liver stages. Using RNA_sequencing to compare the transcriptomics profile between the freshly isolated and cultured PHH, we identified upregulation of the Wnt (Wingless and Int 1), TGF-β (Transforming Growth Factor Beta), Notch and Rho signal~~l~~ing pathways. Treatment with specific chemical antagonists of some pathways led to improvement in Pf schizont development for some pathways. Furthermore, the Wnt pathway was shown to be most influential in determining the size of the growing Pf schizont.

Results:

In vitro cultured PHHs lose permissibility for Pf liver stage infection and development

Traditionally fresh primary human hepatocytes (PHH) are cultured in standard medium containing human sera (WBH) [23, 26, 27] and infected with Pf at 48 hours after plating. However, cultured hepatocytes quickly lose their cellular identity, and this may impact on the host cells' permissibility to sustain Pf infection. To investigate the effect of longer culturing, we infected PHH at different time points post-plating (p.p) with different Pf strains with

different developmental kinetics (PfNF54, PfNF175 and PfNF135 [23]); schizont numbers were evaluated at five days post-infection (p.i) (Figure 1A). We observed a sharp decrease in number of schizonts in relation to the p.p. period for all Pf strains tested with the most drastic decline occurring between days 2 and 5 p.p. This reduction in the number of infected cells could not be explained by a decrease in the number of viable hepatocytes, which became prominent only after day 9 p.p (Figure 1C).

A previous study has shown that medium containing human sera drastically affects function of hepatocytes [21]. Therefore, we examined the impact of sera on hepatocyte permissiveness for parasite development via the removal of sera (WB versus WBH) (Figure S1). Only mild improvements in schizont numbers and the presence of the maturation marker PfMSP1 were observed in sera negative medium (WB)~~ as well as presence of the maturation marker PfMSP1~~. We hence concluded that a novel hepatic culture medium is needed to ~~return~~ restore schizont numbers to those numbers seen on~~of~~ day 2 p.p. PHH. A serum replacement supplement (B27) on a William's E Glutamax base was tested given its beneficial effect for ~~hepatic~~ differentiation of stem cells [28] and hepatic organoids into hepatocytes [29, 30] as well as other hepatic culture systems [24]. Addition of B27 to into the basal medium William's E Glutamax substantially delayed the loss of hepatocyte permissiveness by at least 7 days (Figure 1D). However, this was not due to changes in hepatocyte numbers which remained comparable to the WBH medium thus reflecting a change in the phenotype of the PHHs (Figure 1E).

Irrespective of these positive effects on Pf infections, uninfected PHHs cultured for 7 days displayed a transcriptional profile that was vastly different from freshly isolated PHH showing an upregulation of ~~2403~~ 2420 gene transcripts and a down regulation of ~~2732~~ 2756 genes (Figure 1G). The most significantly up-regulated genes were those involved in actin filament organization, cell-substrate adhesions, wound healing and extracellular matrix organization (Figure 1H). All these pathways are classically associated with epithelial to mesenchymal transition (EMT) [3]. As for down-regulated genes, we found a strong enrichment for those involved in metabolism i.e. ~~small molecule catabolism,~~ fatty acid metabolism, lipid catabolism, ~~organic acid catabolism~~organic anion transport and alcohol metabolism among others (Figure 1F). Together, while serum replacement (via B27) has a substantial positive effect on PHH permissiveness to Pf infection, it cannot fully prevent the loss of hepatic functions, which may be potentially relevant for the maturation of liver schizonts.

Specific host signal transduction pathways influence Pf liver stage development

~~Testing the potential relevance of specific signalling pathways in culture-induced upregulation of genes~~Transcriptomic data (Figure 1F) showed a strong enrichment of genes associated with Wnt (Wingless and Int-1) signa~~l~~ling, Ras protein signa~~l~~ling, transforming growth factor beta (TGF-β), ~~intrinsic~~ apoptotic signa~~l~~ling pathway and Notch signa~~l~~ling pathways (Figure 2A). Compared to the fresh PHH, genes involved in the Wnt, TGF-β and Notch pathways were strongly upregulated (Figure 2B-D), while BMP and Adenylyl-cyclase pathways mildly upregulated in B27 cultured PHH (Figure 2E). To examine the impact of these host pathway on Pf liver stage development, we treated PHH for 6-8 days p.p., with selective modulators of the individual pathways (IWP2, SB431542, DAPT, LDN193189 and Forskolin) (Figure 2F-J). Quantitative measurements assessed the replication (size, nuclear DNA content via DAPI; Figure 2F-G), health (human glutamine synthetase: hGS; Figure S2B-C) and maturation (PfMSP1; Figure 2I) status of the schizonts, respectively (Figure 2F-J, Figure S3). Modulation of both the BMP and the adenylyl-cyclase pathways through the usage of LDN193189 and Forskolin, respectively did not result in significant improvements in neither the number of schizonts (Figure 2F) nor phenotypic parameters. DAPT-treated hepatocytes have significant improvements in the number and size of schizonts observed but not in other parasite phenotypic parameters. ~~DAPT-treated hepatocytes contained parasites displayed limited, but significant improvements in the number and the size of the schizonts.~~ Improvements were found by SB431542 treatment for all five parameters most notably in number of schizonts and expression of maturation marker, PfMSP1. Similarly, in IWP2-treated hepatocytes ~~schizonts~~ showed ~~improvements~~ significant improvements ~~especially~~ in the sizes of the schizonts, DNA content per schizont and expression of PfMSP1 per schizont. Finally, we tested a previously published cocktail containing all five of these inhibitors (5C) [24] (Figure S3). Interestingly, hepatocyte monolayers cultured in the presence of 5C showed poor support of PfNF175 schizont size and numbers (Figure S4D-J). Together, these data demonstrated that inhibition of specific host pathways, and in particular Wnt, TGF-β, are important for supporting Pf permissiveness and/or schizont development.

Inhibition of the Wnt preserves metabolic profile and epithelial nature of PHHs and improves developmental kinetics of Pf

To study the earliest impact of the Wnt pathway on Pf liver stage development, PHHs were treated with ~~either~~ IWP2 (6 days) prior to and during Pf infection (Figure 3A-F). IWP2

treatment promoted parasite development (size, nuclear content and PfMSP1 expression) from day 3 p.i. onwards for all Pf strains. To further capture the transcriptional changes associated with IWP2 treatment, RNA sequencing was performed on hepatocytes cultured in B27 ("mock") or treated with IWP2 or 5C cocktail at day 5 p.i. The original experiment was performed with five replicates per culture/treatment condition, but upon closer inspection we decided to exclude replicate 2 from our analysis owing to suboptimal quality (Figure S6A). Based on spatial proximity in the PCA space, B27 cultured cells were very distinct from freshly isolated "fresh" hepatocytes (67% of variance in the PC1 axis, Figure 3G) as also evident from Figure 1F-H. Upon IWP2 treatment we observed a notable shift of B27 cultured samples towards fresh samples on the PCA space. To quantify this drift, we calculated the sample distance scores which indicated increased similarity between IWP2-treated cells and fresh hepatocyte (mean distance score: 168) as compared to B27 cultured cells (mean distance score: 147) (lower scores define higher similarity between samples). Thus, treatment with IWP2 suppressed the transcriptional changes observed during the *in vitro* culture in B27 medium ~~(possibly associated with loss of native tissue context)~~. Interestingly, 5C-treatment elicited additional transcriptional changes apparently not beneficial for Pf development (Figure 3G). In IWP2-treated cells, 394 genes were upregulated and 344 genes downregulated relative to B27 cultured ("mock") cells, (Figure 3I). Gene Ontology analysis of the genes upregulated in IWP2 treated cells revealed enrichment of metabolism associated terms like fatty acid, alcohol and steroid metabolism (Figure 3J). Among the biological processes suppressed upon IWP2 treatment, we found enrichment of extra-cellular matrix organization, surface-adhesion, epithelium migration, response to TGF-β and Wnt-signaling (Figure 3H). ~~Furthermore~~Importantly, IWP2 treatment mainly suppressed ~~a subset of~~ genes (252 genes) that are otherwise upregulated during in vitro culture in B27 medium (Figure 3K).

~~We subsequently investigated the impact of IWP2 treatment on elements of the Wnt signaling pathway and identified modulation of core signaling components (e.g. suppressed expression of ligand: WNT; receptor: Frizzled; transcription factor: TCF/LEF) (Figure 3L). The most likely effect of the repression of the signaling pathway is the downregulation of associated target genes and consequent alteration of the cellular state. To assess this we investigated the activity of key transcription factors (via quantifying the deregulation of their targets genes). As expected the Wnt-associated transcription factors (e.g. beta catenin/CTNNB1, TFC7L2) displayed increased activity during *in vitro* culturing and most of their target genes displayed clear down-regulation upon IWP2 treatment (Figure S6B-D). Next to these we also observed altered activity of the SMAD (TGF-β) and AP1 (noncanonical WNT) transcription factors,~~

which is likely due to indirect effect of WNT deregulation (Figure S6B). This is in agreement with published literature stating the interlinked nature of the two pathways (WNT and TGF-β). However, given the lack of impact of TGF-β inhibitor (SB431542) alone on schizont size (Figure 2G), we are confident of the WNT pathway's impact on parasite development. We, hence, concluded that IWP2 treatment improves the metabolic profile of cultured hepatocytes while simultaneously suppressing dedifferentiation processes like epithelium to mesenchymal transition, which associates with an improved developmental kinetics of liver schizonts. This observation is directly linked with suppression of core Wnt signaling elements and downstream gene regulatory activity.

Activation status of the Wnt pathway determines the size of Pf schizonts

We subsequently investigated the impact of IWP2 treatment on elements of the Wnt signaling pathway and identified modulation of core signaling components (e.g. suppressed expression of ligand: WNT; receptor: Frizzled; transcription factor: TCF/LEF) (Figure 3L). The most likely effect of the repression of the signaling pathway is the downregulation of associated target genes and consequent alteration of the cellular state. To assess this we investigated the activity of key transcription factors (via quantifying the deregulation of their targets genes). As expected the Wnt-associated transcription factors (e.g. beta-catenin/CTNNB1, TFC7L2) displayed increased activity during *in vitro* culturing and most of their target genes displayed clear down-regulation upon IWP2 treatment (Figure S6B-D). Next to these we also observed altered activity of the SMAD (TGF-β) and AP1 (noncanonical WNT) transcription factors, which is likely due to indirect effect of WNT deregulation (Figure S6B). This is in agreement with published literature stating the interlinked nature of the two pathways (WNT and TGF-β, REFs) [31-33](Guo Cell Research 2009; Luo CSH 2017; Guo Genes and Dev 2008). However, given the lack of impact of TGF-β inhibitor (SB431542) alone on schizont size (Figure 2G), we are confident of the WNT pathway's impact on parasite development.

To further examine the impact of the Wnt pathway on Pf schizont size, we treated PHHs with the Wnt activator CHIR99021 prior and during PfNF135 and PfNF175 infection (Figure 4A-D). Compared to the B27 control, the number of schizonts were mildly increased for IWP2 treated PHHs but not consistently changed for CHIR99021 (Figure S4A-B). As predicted, schizonts in CHIR99021-treated PHHs were slightly but significantly smaller than B27 cultured schizonts on day 5 p.i. for both Pf strains. There was also a significant size decrease for PfNF135 but not for PfNF175 at day 7 which may be the result of arrested development of

Formatted: Highlight

PfNF175 under B27 conditions (Figure S3). CHIR99021 treatment also resulted in a relative decrease in PfMSP1 expression significant for PfNF175. Furthermore, pretreatment of hepatocytes with IWP2 or CHIR99021 was followed by return to WBH after NF175 infection, schizont features (i.e. size, DAPI content and PfMSP1 level) were comparable to schizonts observed in hepatocytes cultured in WBH the entire time. Thus, both inhibition and activation of the Wnt pathway in hepatocytes affects predominantly schizont size and maturation. Collectively, it demonstrates that the essential role played by the Wnt signa~~l~~ling pathway of host hepatocytes in determining schizont size and potentially developmental kinetics.

Discussion

In this study, we show that primary human hepatocytes in *in vitro* culture quickly lose their ability to sustain a Pf infection, irrespective of the parasite strain. This loss of permissibility coincides with up-regulation of specific signa~~l~~ling pathways in hepatocytes. Inhibition of individual up-regulated pathways (Wnt, TGF-β, and Notch) results in the enhanced or maintained hepatocyte permissibility and/or improvement in schizont development. In addition, we identify the Wnt-pathway as an important host factor that determines the size and maturity of the developing schizonts.

~~Although~~ The combined blockage of ~~all these~~ four of the up-regulated pathways (Wnt, TGF-β, Notch and BMP) with an adenylate cyclase activator (Forskolin) i.e. the 5C cocktail greatly benefits hepatitis B virus infection as shown previously [19].~~,~~ However, this~~it~~ appears detrimental to hepatocytes permissiveness and development of Pf as shown in this study (Figure S4). This could be due to the differing intracellular host requirements by the two distinct pathogens: for example, the intrahepatic growth of HBV is 72 hours compared to the 7 days of Pf. Furthermore, 5C treatment appears to exert transcriptional and/or cellular changes beyond the prevention of EMT (~~Figure 3A~~Figure S6). In case of Pf, we show here that specific individual host pathways rather than the combination of all pathways bring strong improvement on parasite development.

We find that the inhibition of the Wnt pathway in cultured hepatocytes have a marked effect on parasite size and maturation. This increase in parasites size is concordant with the upregulation of genes involved in lipid, alcohol and steroid metabolism (Figure 4). While general metabolic health of hepatocyte is conceivably beneficial for parasite growth, fatty acid synthesis in

particular is relevant to parasite development and formation of hepatic merozoites [34]. Furthermore, upon Wnt inhibition we find a downregulation of genes typical for EMT transition. In the context of *in vitro* cultures EMT is mainly due to the loss of tissue environment. EMT however can also occur *in vivo* during liver fibrosis [35] and might influence liver stage infection.

While our study highlights the relevance of the inhibition of the Wnt signaling pathway in *in vitro* cultures, this observation might also be relevant during *in vivo* zonal differentiation of hepatocytes. It is well established that within the liver lobule hepatocytes display marked difference in gene expression and metabolism along the portal central axis (also refer to as zonation, [36]) and components of Wnt pathway are predominantly present in zone 3 hepatocytes and heavily involved in maintaining hepatic zonation [37]. We have previously shown that Pf parasites strongly prefer zone 3 (pericentral) hepatocytes resulting in better parasite development [23], as similarly shown for *P. berghei* [38]. While zone 3 hepatocytes possess all components of the Wnt pathway i.e. the surface receptor and intracellular signalling components, the actual external Wnt ligand/signalling proteins may be possibly secreted by hepatocytes from neighbouring zones or other non-hepatocyte cells present in the liver [39] and may therefore also play a role in determining schizont size.

The slight increase in schizont size observed with the other pathway inhibitors may possibly also relate to the Wnt status. Schizonts grown in PHHs treated with forskolin (adenylate cyclase activator) or LDN193189 (inhibitor of Bone Morphogenic Protein pathway) are the relatively smaller with lower PfMSP1 expression. In forskolin treated schizonts, activated adenylate cyclase leads to increased levels of cAMP which in turn can activate the Wnt pathway[40, 41]. However, the relationship between the BMP pathway (LDN193189) and the Wnt pathway is less obvious as the former can either inhibit or activate the Wnt pathway depending on the presence of wildtype of p53 or the loss of SMAD4 respectively [42]. This may explain the large variation observed in schizonts grown with LDN193189. Inhibition of the Notch pathway (by DAPT) maintains PHHs in their hepatocyte lineage (as opposed to a more bile ductal phenotype) as well as indirect inhibition the Wnt pathway [43]. This could hence explain the observation that, schizonts grown in DAPT-treated PHHs are larger compared to the control B27 medium. Finally, TGF-β signalling leads to the activation of the Wnt pathway [44]

therefore its inhibition results in relative better schizonts as for size, nuclear content, and maturation.

Despite their differences in infectivity and phenotype, schizonts of all tested Pf strains are significantly larger and more mature in IWP2-treated PHHs than their counterparts in B27-treated PHHs. This larger size becomes significant during the process of schizont growth i.e. after day 3 p.i. and becomes prominent at day 5 p.i.; blockage of Wnt communication between infected- and uninfected cell could allow for better expansion of the growing schizont with less of host restriction. It has been estimated that an infected hepatocyte can expand up to 200 times its original volume to accommodate the growing Pf schizont [45]. This could necessitate some communication between the infected and uninfected hepatocyte and the Wnt signalling pathway may serve here as communication platform [33,35-37]. IWP2 specifically inhibits the enzyme, porcupine (Porcn), which is involved in transport of the wnt ligands [46]. One could hypothesize that neighbouring hepatocytes keep their strict size and volume via the steady basal level of wnt signalling and thereby limiting the growth of Pf schizonts [47]. A possible mechanism of action is summarised in Figure 5.

In conclusion, we show that PHHs cultured in the standard medium (WBH) rapidly lose their permissiveness to Pf liver stage development. This loss, however, can be substantially delayed by the usage of a defined serum replacement (B27) on top of a William's E Glutamax base. Furthermore, we identify three specific host signalling pathways (Wnt, TGF-β and Notch) to modulate Pf liver stage development. In particular, the Wnt signalling pathway is shown in a novel role to control the size of the developing Pf schizont and is therefore a key factor in determining the Pf developmental kinetics for multiple parasite strains. Transcriptome analysis of Wnt inhibitor treated hepatocytes indicates that improvement of hepatic metabolism, prevention of EMT and cell division may lead to the positive effects on parasite size and maturation. Together, these findings provide steps forward in understanding the intricate interaction between host and parasite within infected hepatocytes which may benefit rational approaches for future therapeutic interventions.

Materials and Methods

Ethics

Primary human liver cells were freshly isolated from remnants of surgical material. The samples were anonymised and general approval for their use was granted in accordance with the Dutch ethical legislation as described in the Medical Research (Human Subjects) Act. It was confirmed by the Committee on Research involving Human Subjects in the region of Arnhem-Nijmegen, the Netherlands. Approval for use of remnant, anonymized surgical material for transcriptome analysis was specifically confirmed by the Committee on Research involving Human Subjects, in the region of Arnhem-Nijmegen, the Netherlands (CMO-2019-5908).

PHH isolation from liver segments

Primary human hepatocytes were isolated from patients undergoing elective partial hepatectomy as previously described [23, 27]. Freshly isolated hepatocytes are suspended in complete William's B medium (WB): William's E with glutamax (ThermoFisher Scientific: 32551087), 1x Insulin/transferrin/selenium (ThermoFisher Scientific: 41400045), 1mM sodium pyruvate (ThermoFisher Scientific: 11360070), 1x MEM Non-essential amino acid solution (ThermoFisher Scientific: 11140035), 100 units/ml Penicillin-Streptomycin (ThermoFisher Scientific: 15140122) and 1.6µM of dexamethasone (Sigma Aldrich: D4902). PHHs were plated at 62,500 cells per well in 96 well format and kept in a 37°C (5% CO_2) incubator with daily medium changes 96-well plates (Falcon: 353219) precoated with Type 1 collagen solution from rat tail (Sigma Aldrich: C3867).

Generation of sporozoites for liver infection

Pf asexual and sexual stages were cultured in a semi-automatic system as described [48-50]. *Anopheles stephensi* mosquitoes were reared at the Radboud University Medical Center Insectary (Nijmegen, the Netherlands) in accordance with standard operating procedures. Salivary glands from infected mosquitoes (day 14 to day 23 post infection) were hand-dissected and collected in WB medium. Collected glands were homogenized using home-made glass grinders and sporozoites were counted in a Burker-Turk chamber using phase-contrast microscopes. Immediately prior to infection of human hepatocytes, the sporozoites were supplemented with heat-inactivated human sera (HIHS) at 10% of the total volume (i.e. WBH). The multiplicity of infection is 0.8 (e.g. 50.000 spz to 62500 hepatocytes) or 1 (62500 spz to 62500 hepatocytes) depending on the yield of the sporozoite batches. The MOI is the same

within an experiment/biological replicate but may differ between experiments (as it depended on the sporozoite yield of the dissected mosquitoes.

Medium compositions and duration

Different plating time after isolation (Figure 1B, C): Isolated PHH were plated in WB medium. The following day, it was changed to WBH: this medium was refreshed daily until the end of the experiment on day 26 post plating. In figure 1D and E, isolated PHH were again plated in WB medium. The following day, it was changed to William's E with glutamax (ThermoFisher Scientific: 32551087) supplemented with 1x B27~~TM~~ Supplement (ThermoFisher Scientific: 17504044) and 100 units/ml Penicillin-Streptomycin (ThermoFisher Scientific: 15140122) which was referred to as B27 medium or mock in the article. On the day of infection, B27 medium was removed and replaced with sporozoites suspended in WBH for three hours. After the infection process has occurred (i.e. after three hours), the WBH (sporozoite) medium was replaced with B27 medium until the conclusion of the experiment.

Different medium treatments: Isolated PHH were plated in WB medium. The following day, it was changed to the following medium conditions: B27 or B27 supplemented with 20µM of Forskolin (Enzo Life Sciences: BML-CN100-0010), or 10µM of SB431542 (Tocris: 1614), or 0.5µM of IWP2 (Tocris: 3533), or 5µM of DAPT (Tocris: 2634) or 0.1µM of LDN193189 (Tocris: 6053) or the combination of all the compound inhibitors (i.e. 5C). The hepatocytes were kept on this treatment for another 6 days (i.e. 7 days post plating) and then infected with sporozoites where the medium composition changes to WBH for three hours. After three hours, the monolayers were returned to their respective treatments. Media was refreshed on a daily (24hour) basis.

Investigating the Wnt pathway: Isolated PHH were plated in WB medium. The following day, it was changed to the following medium conditions: B27 or B27 supplemented with 0.5µM of IWP2 or 3µM CHIR-99021 (Sigma-Aldrich: SML1046). The hepatocytes were kept on this treatment for another 6 days (i.e. 7 days post plating) and then infected with sporozoites where the medium composition changes to WBH for three hours. After three hours, the monolayers were returned to their respective treatments. Media was refreshed on a daily (24hour) basis.

Immunofluorescence readout

Monolayers were fixed with 4% paraformaldehyde (ThermoFisher Scientific: 28906) for 10 minutes and permeabilised using 1% Triton for 5 minutes. The samples are stained with the various primary Pf or human antibodies: Rabbit PfHSP70 at 1:75 dilution (StressMarq Biosciences: SPC186), Mouse PfMSP1 at 1:100 dilution (Sanaria and NIH/NIAD: AD233), and mouse human glutamine synthethase at 1:100-250 dilution (Abcam: ab64613). Secondary antibodies were used at these following dilutions: Goat anti-rabbit Alexa Fluor 594 at 1:200 dilution (ThermoFisher Scientific: A11012) and goat anti-mouse Alexa Fluor 488 at 1:200 dilution (ThermoFisher Scientific: A11029). DAPI was used at 300 nM to stain the nuclear material of the monolayer.

Microscopy

The Zeiss LSM880 with Airyscan at 63x objectives (oil) and 2x zoom were used for detailed images. For high content images, the Zeiss Axio Observer Inverted Microscope Platform with AI assisted experimental startup was used. The images were acquired at 20x objectives with a numerical aperture of 0.8.

Data analysis using FIJI

Infection rate: For each well, 77 images were acquired in a tiled format. Approximately half (i.e. 39) images were counted on FIJI [51] for NF135 and NF175 infections. All the tiles were counted for NF54 due to the lower infection rate. The number of hepatic nuclei were counted for 1% of the total image (i.e. 7-10 images) and then extrapolated to get number of PHHs per well.

Measurement of schizont size: See Yang et al for further details [23]. Images obtained on the high content microscope were opened in FIJI. Random images were chosen until at least 100 schizonts were measured (per well) unless the infection is with NF54 (at least 50 schizonts). Schizonts were selected using the region of interest (ROI) tool based on PfHSP70 positivity (red channel) and measured. This ROI mask is applied onto the other colour channels i.e. blue and green to obtain values of nuclear content (DAPI) and hGS or PfMSP1 signal. For hGS and PfMSP1, background non-specific staining was considered and subtracted from the final signal. Further details regarding the methods can be found in [21, 23].

RNA Isolation and RNA sequencing library preparation

Freshly isolated or in vitro cultured hepatocytes (mock or treatment) were homogenized in TRIzol solution and cryopreserved at -80°C. RNA was subsequently isolated using the Zymo research Direct zol RNA purification kit (Cat. No. #R2053). The isolated RNA was assessed for quality and quantified using Nanodrop and agarose gel electrophoresis. RNA sequencing libraries from isolated/purified RNA were prepared using the Kapa mRNA Hyperprep Kit (Cat. No. #KK8581) using slight modifications of the manufacturer's instructions for mRNA enrichment method. The input total RNA for the various libraries was selected between 150ng, 250ng or 500ng. The enriched mRNA was fragmented at 94°C for 6min and dA tailing performed at 55°C for 15min. In order to better capture the AT rich *Pf* transcriptome we modified the PCR protocol as follows: Initial denaturation at 98°C for 2min; Cycling denaturation at 98°C for 20sec, annealing + extension at 62°C for 2min; final extension at 62°C for 3min. We selected PCR cycles for library amplification (12, 11 and 10 respectively) based on starting input total RNA amount. The amplified libraries were quantified using Denovix dsDNA high sensitivity kit (Cat. No. #TN145)on a Qubit fluorometer. Further qualitative assessment of library size distribution was performed using the Agilent high sensitivity DNA kit (Cat. No. #5067-4626) on the Agilent 2100 bioanalyzer platform. Each library was sequenced in 42bp paired-end format for roughly 18 million reads on the Illumina Nextseq 500 platform.

RNA sequencing data analysis

The sequencing endline fastq files were quality checked using FASTQC (ver. 0.11.9). The reads were subsequently trimmed for quality (-q 30) and adapter removal using the Trim_Galore software (ver. 0.6.7). The trimmed reads were aligned onto the *Homo sapiens* ver. 38 genome using STAR aligner (ver. 2.7.10a). The reads mapped onto the respective genomes were counted using the –quantMode GeneCounts option in STAR. Reads mapping only to sense strand were corrected for batch effects stemming from different hepatocyte donors using the Combat-seq tool (sva package ver. 3.44.0). The batch corrected counts were used for subsequent differential expression analysis (for conditions/treatments) in DESEq2 package (ver. 1.36.0) in RStudio (ver. 4.2.2 "Innocent and Trusting"). PCA plot for the various sequenced libraries/samples was generated using plotPCA command. Volcano plots for differentially expressed genes and genes of interest were generated using custom scripts and ggplot2 (ver. 3.4.2) in R. Gene Ontology enrichment analysis was performed in using the clusterProfiler tool (ver. 4.4.4) with subsequent dot-plots generated using inbuilt functions in ggplot2. Heatmaps for differentially expressed genes were generated using the Morpheus

online tool by Broad Institute and custom scripts in R (ggplot2). KEGG pathway schematic for Wnt signaling was generated using the pathview tool package (ver. 1.46.0) in R. Transcription factor activity analysis was performed using the decoupleR package (ver. 2.6.0 utilizing collecTRI database) from the Julio Saez-Rodriguez lab [52, 53]. Bar-plot visualization for TF activity scores was done in GraphPad Prism 8.

Statistical analysis

For the majority of the experiments, at least three biological replicates were performed with two technical replicates. All statistical tests were performed using Prism 10. See figure legends for details regarding the statistical tests.

Data availability

The next-generation sequencing dataset associated with this study has been uploaded to Gene Expression Omnibus: GSE263643

The dataset comprises of:

The metadata sheet with information on experiments and samples

Raw data files: fastq files for RNA-sequencing

Processed data files: i) raw counts files generated from Combat-seq and ii) normalized count files generated from DESeq2

Author contributions:

AK, GJG, HW, AS, and ASPY performed the experiments. JHWdW coordinated the collection of the fresh human liver segments. ASPY and AK performed the analysis on the data. TB was involved in the writing and reading of the manuscript. AK, HW, RWS, RB and ASPY were involved in the conceptualization and writing of the manuscript.

Acknowledgements:

We are grateful for R. Stoter, W. Graumans, M. Vegte-Bolmer, A. Pouwelsen, L. Pelser-Posthumus and J. Kuhnen of the Malaria and Insectary Unit at the Radboud University Medical Center for parasite, mosquito and sporozoite production. We would like to thank the Microscopic Imaging Center (MIC) of the Radboud University for access to its facilities. A.S.P.Y was a recipient of the Veni grant from the Dutch Research Council (NWO) talent

program (VI.Veni.192.171) and ZonMW Off-Road grant (04510012010050). A.K was supported by the Dutch Research Council (ZonMW-TOP-grant #91218010).

Financial Disclosure:

The salary of A.S.P.Y was supported by the Dutch Research Council (NWO) talent program (VI.Veni.192.171) and ZonMW Off-Road grant. A.K. was supported by the Dutch Research Council (ZonMW-TOP-grant #91218010). However, the funders had no role in study design, data collection, analysis, decision to publish or preparation of the manuscript.

References:

1. WHO, W.H.O., *World Malaria Report 2022*. 2022.
2. Vaughan, A.M., et al., *Complete Plasmodium falciparum liver-stage development in liver-chimeric mice.* J Clin Invest, 2012. **122**(10): p. 3618-28.
3. Mo, A.X. and G. McGugan, *Understanding the Liver-Stage Biology of Malaria Parasites: Insights to Enable and Accelerate the Development of a Highly Efficacious Vaccine.* Am J Trop Med Hyg, 2018. **99**(4): p. 827-832.
4. Langlois, A.C., et al., *Plasmodium sporozoites can invade hepatocytic cells independently of the Ephrin receptor A2.* PLoS One, 2018. **13**(7): p. e0200032.
5. Silvie, O., et al., *Expression of human CD81 differently affects host cell susceptibility to malaria sporozoites depending on the Plasmodium species.* Cell Microbiol, 2006. **8**(7): p. 1134-46.
6. Silvie, O., et al., *Hepatocyte CD81 is required for Plasmodium falciparum and Plasmodium yoelii sporozoite infectivity.* Nat Med, 2003. **9**(1): p. 93-6.
7. Mancio-Silva, L., et al., *Nutrient sensing modulates malaria parasite virulence.* Nature, 2017. **547**(7662): p. 213-216.
8. Lahree, A., J. Mello-Vieira, and M.M. Mota, *The nutrient games - Plasmodium metabolism during hepatic development.* Trends Parasitol, 2023. **39**(6): p. 445-460.
9. Posfai, D., et al., *Plasmodium parasite exploits host aquaporin-3 during liver stage malaria infection.* PLoS Pathog, 2018. **14**(5): p. e1007057.
10. Rodrigues, C.D., et al., *Host scavenger receptor SR-BI plays a dual role in the establishment of malaria parasite liver infection.* Cell Host Microbe, 2008. **4**(3): p. 271-82.
11. Real, E., et al., *Plasmodium UIS3 sequesters host LC3 to avoid elimination by autophagy in hepatocytes.* Nat Microbiol, 2018. **3**(1): p. 17-25.
12. Thieleke-Matos, C., et al., *Host cell autophagy contributes to Plasmodium liver development.* Cell Microbiol, 2016. **18**(3): p. 437-50.
13. van de Sand, C., et al., *The liver stage of Plasmodium berghei inhibits host cell apoptosis.* Mol Microbiol, 2005. **58**(3): p. 731-42.
14. Ebert, G., et al., *Targeting the Extrinsic Pathway of Hepatocyte Apoptosis Promotes Clearance of Plasmodium Liver Infection.* Cell Rep, 2020. **30**(13): p. 4343-4354 e4.
15. Soulard, V., et al., *Plasmodium falciparum full life cycle and Plasmodium ovale liver stages in humanized mice.* Nat Commun, 2015. **6**: p. 7690.
16. Sacci, J.B., Jr., et al., *Plasmodium falciparum infection and exoerythrocytic development in mice with chimeric human livers.* Int J Parasitol, 2006. **36**(3): p. 353-60.
17. Sattabongkot, J., et al., *Establishment of a human hepatocyte line that supports in vitro development of the exo-erythrocytic stages of the malaria parasites Plasmodium falciparum and P. vivax.* Am J Trop Med Hyg, 2006. **74**(5): p. 708-15.
18. Tweedell, R.E., et al., *The Selection of a Hepatocyte Cell Line Susceptible to Plasmodium falciparum Sporozoite Invasion That Is Associated With Expression of Glypican-3.* Front Microbiol, 2019. **10**: p. 127.
19. Kaushansky, A., et al., *Malaria parasites target the hepatocyte receptor EphA2 for successful host infection.* Science, 2015. **350**(6264): p. 1089-92.
20. Roth, A., et al., *A comprehensive model for assessment of liver stage therapies targeting Plasmodium vivax and Plasmodium falciparum.* Nat Commun, 2018. **9**(1): p. 1837.

21. Yang, A.S.P., et al., *Development of Plasmodium falciparum liver-stages in hepatocytes derived from human fetal liver organoid cultures.* Nat Commun, 2023. **14**(1): p. 4631.
22. Yalaoui, S., et al., *Scavenger receptor BI boosts hepatocyte permissiveness to Plasmodium infection.* Cell Host Microbe, 2008. **4**(3): p. 283-92.
23. Yang, A.S.P., et al., *Zonal human hepatocytes are differentially permissive to Plasmodium falciparum malaria parasites.* EMBO J, 2021. **40**(6): p. e106583.
24. Xiang, C., et al., *Long-term functional maintenance of primary human hepatocytes in vitro.* Science, 2019. **364**(6438): p. 399-402.
25. Gomez-Lechon, M.J., et al., *Competency of different cell models to predict human hepatotoxic drugs.* Expert Opin Drug Metab Toxicol, 2014. **10**(11): p. 1553-68.
26. Mazier, D., et al., *Complete development of hepatic stages of Plasmodium falciparum in vitro.* Science, 1985. **227**(4685): p. 440-2.
27. Walk, J., et al., *Modest heterologous protection after Plasmodium falciparum sporozoite immunization: a double-blind randomized controlled clinical trial.* BMC Med, 2017. **15**(1): p. 168.
28. Mallanna, S.K. and S.A. Duncan, *Differentiation of hepatocytes from pluripotent stem cells.* Curr Protoc Stem Cell Biol, 2013. **26**: p. 1G 4 1-1G 4 13.
29. Huch, M., et al., *Long-term culture of genome-stable bipotent stem cells from adult human liver.* Cell, 2015. **160**(1-2): p. 299-312.
30. Hu, H., et al., *Long-Term Expansion of Functional Mouse and Human Hepatocytes as 3D Organoids.* Cell, 2018. **175**(6): p. 1591-1606 e19.
31. Guo, X. and X.-F. Wang, *Signaling cross-talk between TGF-β/BMP and other pathways.* Cell research, 2009. **19**(1): p. 71-88.
32. Luo, K., *Signaling cross talk between TGF-β/Smad and other signaling pathways.* Cold Spring Harbor perspectives in biology, 2017. **9**(1): p. a022137.
33. Guo, X., et al., *Axin and GSK3-β control Smad3 protein stability and modulate TGF-β signaling.* Genes & development, 2008. **22**(1): p. 106-120.
34. van Schaijk, B.C., et al., *Type II fatty acid biosynthesis is essential for Plasmodium falciparum sporozoite development in the midgut of Anopheles mosquitoes.* Eukaryot Cell, 2014. **13**(5): p. 550-9.
35. Fabris, L., et al., *Revisiting Epithelial-to-Mesenchymal Transition in Liver Fibrosis: Clues for a Better Understanding of the "Reactive" Biliary Epithelial Phenotype.* Stem Cells Int, 2016. **2016**: p. 2953727.
36. Gebhardt, R., *Metabolic zonation of the liver: regulation and implications for liver function.* Pharmacol Ther, 1992. **53**(3): p. 275-354.
37. Russell, J.O. and S.P. Monga, *Wnt/beta-Catenin Signaling in Liver Development, Homeostasis, and Pathobiology.* Annu Rev Pathol, 2018. **13**: p. 351-378.
38. Afriat, A., et al., *A spatiotemporally resolved single-cell atlas of the Plasmodium liver stage.* Nature, 2022. **611**(7936): p. 563-569.
39. Zeng, G., et al., *Wnt'er in liver: Expression of Wnt and frizzled genes in mouse.* Hepatology, 2007. **45**(1): p. 195-204.
40. Suzuki, A., et al., *PTH/cAMP/PKA signaling facilitates canonical Wnt signaling via inactivation of glycogen synthase kinase-3beta in osteoblastic Saos-2 cells.* J Cell Biochem, 2008. **104**(1): p. 304-17.
41. Guo, R., et al., *Targeting Adenylate Cyclase Family: New Concept of Targeted Cancer Therapy.* Front Oncol, 2022. **12**: p. 829212.

42. Voorneveld, P.W., et al., *The BMP pathway either enhances or inhibits the Wnt pathway depending on the SMAD4 and p53 status in CRC.* Br J Cancer, 2015. **112**(1): p. 122-30.
43. Geisler, F. and M. Strazzabosco, *Emerging roles of Notch signaling in liver disease.* Hepatology, 2015. **61**(1): p. 382-92.
44. Bogaerts, E., et al., *The roles of transforming growth factor-beta, Wnt, Notch and hypoxia on liver progenitor cells in primary liver tumours (Review).* Int J Oncol, 2014. **44**(4): p. 1015-22.
45. Vaughan, A.M. and S.H.I. Kappe, *Malaria Parasite Liver Infection and Exoerythrocytic Biology.* Cold Spring Harb Perspect Med, 2017. **7**(6).
46. Wang, X., et al., *The development of highly potent inhibitors for porcupine.* J Med Chem, 2013. **56**(6): p. 2700-4.
47. Acebron, S.P., et al., *Mitotic wnt signaling promotes protein stabilization and regulates cell size.* Mol Cell, 2014. **54**(4): p. 663-74.
48. Ifediba, T. and J.P. Vanderberg, *Complete in vitro maturation of Plasmodium falciparum gametocytes.* Nature, 1981. **294**(5839): p. 364-6.
49. Ponnudurai, T., et al., *Cultivation of fertile Plasmodium falciparum gametocytes in semi-automated systems. 1. Static cultures.* Trans R Soc Trop Med Hyg, 1982. **76**(6): p. 812-8.
50. Ponnudurai, T., et al., *Infectivity of cultured Plasmodium falciparum gametocytes to mosquitoes.* Parasitology, 1989. **98 Pt 2**: p. 165-73.
51. Schindelin, J., et al., *Fiji: an open-source platform for biological-image analysis.* Nat Methods, 2012. **9**(7): p. 676-82.
52. Müller-Dott, S., et al., *Expanding the coverage of regulons from high-confidence prior knowledge for accurate estimation of transcription factor activities.* Nucleic Acids Research, 2023. **51**(20): p. 10934-10949.
53. Badia-i-Mompel, P., et al., *decoupleR: ensemble of computational methods to infer biological activities from omics data.* Bioinformatics advances, 2022. **2**(1): p. vbac016.

Figure 1: *Pf*-schizont development post-hepatocyte plating and transcriptomic profiles of primary human hepatocytes (PHH). A) The solid arrows show the number of days after liver isolation (post-plating) and the lines with the clear triangles show the period of schizont development. B) The number of schizonts in PHH on day 5 post infection (p.i.) when infected on different days p.p. for parasite strains: NF175 (magenta;), NF135 (green;) and NF54 (blue) in two independent experiments (except NF175). C) The number of PHHs present on day 5 p.i. on indicated days p.p for the specific parasite strains. For each independent experiment, the mean of three replicates is shown along with the standard deviation. The number of schizonts (D) and hepatocytes (E) on day 5 post invasion (p.i.) for NF135 and NF54 (n=1) for different infection times post plating (p.p.) cultured in B27 medium (grey). Dot-plots depicting the biological processes downregulated (F) and upregulated (H) in cultured hepatocytes as compared to freshly-isolated hepatocytes. (G) Volcano plot highlighting the differentially

expressed genes between hepatocytes cultured in B27 medium (“mock”) and freshly isolated hepatocytes (“fresh”).

Figure 2: Effect of signal~~l~~ling pathways on Pf schizont development. A) Dot-plot depicting the signaling pathways exhibiting higher activity in *in vitro* cultured hepatocytes as compared to freshly isolated hepatocytes. Heatmaps representing normalized expression of genes from (B)Wnt, (C)TGFβ, (D)Notch, (E)BMP and Adenylate cyclase signal~~l~~ling pathways deregulated in cultured vs freshly isolated hepatocytes. The data is scaled and representative of 2 biological replicates of RNA sequencing. The effect of specific host pathway inhibitors (IWP2, SB431542, DAPT, LDN193189 and Forskolin) on respectively schizont numbers (F; each dot is the average count of two technical replicates), size of schizonts (G; each dot is the median of at least 100 schizonts), total DAPI per schizont (H; each dot is the median of at least 100 schizonts), and total PfMSP1 per schizont (I; each dot is the median of at least 100 schizonts) on day 5 p.i.. The grey dotted line (per graphs F-I) shows the median measurements of schizonts grown in B27 control (from four independent experiments). The p-values from a Dunnett’s multiple comparisons test are shown – see supplementary information for full p values. J) Representative confocal images (from four independent experiments) showing schizonts grown in B27 alone and B27 supplemented with Forskolin, SB431542, IWP2, DAPT and LDN193189 on day 5 p.i. stained with PfMSP1, PfHSP70 and DAPI. Scale bar is 25 microns.

Figure 3: Effect of IWP2 on developmental kinetics of NF175 (A-B). NF135 (C-D) and NF54 (E-F) schizonts. For each Pf strain, the number schizonts, the size of schizonts, the total DAPI content and total PfMSP1 content are shown from left to right. The schizonts are either grown in hepatocytes cultured in either B27 (grey) or B27 supplemented with IWP2 (orange) for 6 days prior to infection and also during schizont development on days 3, 5 and 7 p.i.. Note briefly that day 3 schizonts of NF175 was not examined. For the number of schizonts, the mean of three independent experiments, each with 2-3 internal replicates is shown. For the schizont size and the total DAPI per schizont, at least 100 schizonts per each of three independent experiments (with two replicates per experiment) were measured. The mean of the median from each experiment is plotted and the graph shows the mean with standard deviation. For the total MSP1 content, at least 100 schizonts per each of three independent experiments were measured. The median is plotted and the graph shows the mean with standard deviation. A Dunnett’s multiple comparisons test is performed: see supplementary information for full p values. B, D, F) Representative confocal images (from three independent experiments)

showing NF175, NF135 and NF54 schizonts grown in B27 alone and B27 supplemented with IWP2 on day 7 p.i. stained with MSP1, HSP70 and DAPI respectively. Scale bar is 25 microns. (G) PCA plot highlighting the unique transcriptional states of freshly isolated ("fresh"), cultured ("mock"), IWP2- or 5C-treated hepatocytes. Dot-plots depicting the biological processes downregulated (H) or upregulated (J) in IWP2-treated as compared to "mock" cultured hepatocytes. I) Volcano plot highlighting differential gene expression between IWP2-treatmentand "mock"-cultured hepatocytes. K) Gene expression heatmap highlighting the genes upregulated in mock cultured hepatocytes and suppressed upon IWP2 treatment. The expression counts have been normalized and scaled for plotting. L) Schematic overview of key genes involved in the Wnt signaling pathway that are modulated by IWP2 treatment. The Fold-change (log2) for the genes are scaled and plotted in blue-red color schematic.

Figure 4: The impact of the Wnt signalling pathway on schizont size. Hepatocytes treated with B27 or B27 supplemented with IWP2/CHIR99021 and infected with either NF175 (A) or NF135 (B). Schizont size (left), and total DAPI (middle) content are measured on days 5 and 7 post infection. The total amount of PfMSP1 per schizont was measured on day 7 p.i. (right). Each dot represents a schizont with at least 100 schizonts measured for three independent experiments. The median is shown, and a Dunn's multiple comparisons test is performed with the p values displayed: see supplementary information for full p values. Representative confocal images (from three independent experiments) showing NF175 (C) and NF135 (D) schizonts B27 or B27 supplemented with IWP2/CHIR99021 on day 7 p.i. stained with MSP1, HSP70 and DAPI. Scale bar is 25 microns.

Figure 5: Schematic of a possible mechanism in which the Wnt pathway controls Pf schizont development. A) Under untreated and uninfected condition, there is a steady level of beta-catenin (β-catenin). In addition to its role in gene transcription, β-catenin is the intracellular component that links the cadherin adhesion molecules to actin filaments and therefore controls the "rigidity" of the cells in relation to its neighbours. B) In an infected hepatocyte (after 3 days post infection), the growing Pf schizont takes up so much of the hepatocyte volume that the trafficking of the Wnt ligands to the surface is disrupted. Wnt receptor of the neighbouring uninfected cells are not activated, and β-catenin molecules are degraded due to the phosphorylation of the enzyme, glycogen synthase kinase 3 (GSK3): this allows some flexibility in the uninfected neighbouring cells to accommodate the growth of the infected cell. However in these (uninfected cells), Wnt ligands are still present and can interact

with the existing Wnt receptors on the infected hepatocytes to maintain some β-catenin in the infected cell, thus limiting the growth/size of the schizont. C) Under IWP2 treatment, Wnt ligands are not trafficked to the surface of both uninfected and infected hepatocytes due to the inhibition of the enzyme porcupine (target of IWP2). Porcupine "labels" (via palmitoylation) Wnt ligands for correct trafficking to the plasma membrane. As a result, Wnt receptors on both uninfected and infected cells are not activated and existing β-catenin are degraded leading to reduced connection between cadherin and actin filaments (i.e. cell-cell contacts) and ultimately reducing rigidity. D) Under CHIR99021 (GSK3 inhibitor) treatment, β-catenin in both uninfected and infected cells cannot be phosphorylated nor degraded. Both cell types are more rigid due to the improve connection between actin filaments and cadherin which severly limits the size of the Pf schizont.

Supplementary Figures

Figure S1: The impact of heat inactivated human sera on Pf schizont development. PHH monolayers were cultured in either William's B (WB) or William's B supplemented with 10% heat inactivated sera (WBH) for six days p.p. and infected with either NF175 (A) or NF135 (B) or NF54 (C). Data are shown for respectively schizont number, size, total DAPI content, percentage of human glutamine synthetase (hGS) positive schizonts, total hGS level per schizont and total merozoite surface protein 1 (MSP1) level per schizont. As for the number of schizonts, each dot represents an independent experiment showing the mean of two replicates. As for the size and total DAPI content graphs, each dot represents an independent experiment showing the mean of the median of at least 100 schizonts per replicate (2 replicates) were measured (except in NF54 condition where there is not enough schizonts present). For the percentage of hGS positive schizonts, each dot represents an independent experiment where at least 100 schizonts were examined from one replicate. For the total hGS level and total MSP1 level, each dot represents an independent experiment of the median from at least 100 schizonts (except in NF54 condition where there is not enough schizonts present). The error bars show the mean with the standard deviation. The p-values are generated by performing an unpaired t-test between WBH and WB and can be found in the supplementary information excel sheet (*** = 0.0001).

Figure S2: Effect of specific inhibitors of PHH gene-signalling pathways on the expression Pf schizont markers A) Schematic experimental setup of Figure 3 and S3. PHHs were treated for 6 days p.p with either B27 or B27 supplemented

IWP2/SB431542/Forskolin/DAPT/LDN193189. The experiment was analysed at day 5 p.i.. with NF175 B) Total number of hepatocytes per well with each dot representing the average three wells of one biological replicate. C)Total hGS per schizont with each dot representing the median of at least 100 schizonts. The percentage of hGS (D) and MSP1 (E) positive schizonts: each dot is an independent experiment where at least 100 schizont is measured. The grey dotted line (per graphs C-E) shows the median measurements of schizonts grown in B27 (from four independent experiments) as each host pathway inhibitor treatment was made in media containing B27. The mean and standard deviation is shown for each graph. The p-values from a Dunnett's multiple comparisons test are displayed. F) Representative confocal images (from four independent biological experiments) showing schizonts grown in B27 alone and B27 supplemented with IWP2, SB431542, DAPT, LDN193189, Forskolin stained with hGS, HSP70 and DAPI. Scale bar is 25 microns.

Figure S3: The effect of B27 or B27 supplemented with 5C on *Pf*-permissiveness and schizont development in PHH. The number of schizonts (A) and hepatocytes (B) on day 5 p.i. for NF135 and NF54 (n=1) at indicated time points p.p. when cultured in either B27 medium (grey) or B27 supplemented with 5C (blue). Each dot shows the mean of three replicates and the error bars show the standard deviation. C) Experimental setup of graphs D-L. PHHs were cultured for 6 days p.p. with either B27 or B27 with 5C and infected with NF175 parasites and analysed at day 5 p.i.. D) Total number of hepatocytes per well with each dot representing the average three wells of one biological replicate. E)The number of schizonts per well was normalised to the number of schizonts counted in WBH (red dotted line). Each dot is the mean of an independent experiment with two replicates. For the schizont size (F) and total DAPI content per schizont (G), each dot represents the mean of an independent experiment of at least 100 schizonts. For the total hGS (H) and MSP1 (I) per schizont, each dot represents the median of an independent experiment of at least 100 schizont For the percentage of hGS (J) and MSP1 (K) positive cells, each dot represents an independent experiment of at least 100 schizonts. D-K shows the mean with the standard deviation). Representative confocal images (from four independent experiments) showing schizonts grown in WBH or B27 alone or B27 supplemented with 5C on day 5 p.i. stained with hGS (L) or MSP1 (M), HSP70 and DAPI. Scale bar is 25 microns.

Figure S4: The impact of the Wnt signalling pathway on numbers of developing schizonts. A) The number of schizonts in NF135 infected PHHs treated with B27 or B27 supplemented

with IWP2/CHIR99021. Each graph shows an independent experiment, each with duplicates per condition. B) Number of schizonts (left) and number of hepatocytes (right) in NF175-infected PHHs treated with B27 or B27 supplemented with IWP2/CHIR99021. Each graph shows the mean and the standard deviation of three independent experiments: each experiments have duplicate wells. There was no statistic difference between conditions within the same day post infection. Representative confocal images (from three independent biological experiments) showing NF135 (C) and NF175 (D) schizonts B27 or B27 supplemented with IWP2/CHIR99021 on day 5 p.i. stained with hGS, HSP70 and DAPI. Scale bar is 25 microns.

Figure S5: The impact of Wnt signa~~l~~ling on determining NF175 development in the liver A) schematic showing the media treatment timing and conditions. Hepatocytes are treated with B27 or B27 supplemented with IWP2/CHIR99021. After exposure to NF175, cultures were either kept in B27 or B27 supplemented with IWP2/CHIR99021 (closed circles) or returned to WBH (IWP2* and CHIR99021* - open circles). The number of schizonts (B), size of schizonts (C), total nuclear content per schizont (D) and total MSP1 per schizont (E) on day 5 post infection. The number of schizonts (F), size of schizonts (G), total nuclear content per schizont (H) and total MSP1 per schizont (I) on day 7 post infection. Each dot represents an independent biological donor where the median of at least 100 schizonts were measured. Dunnett's multiple comparison test (to WBH) were performed for each graph and the statistical significance is indicated: see supplemental information excel sheet for the full p values.

Figure S6: IWP2 treatment modulates expression of target genes downstream of Wnt responsive transcription factors. A) PCA plot of all replicates of freshly-isolated, "mock-cultured", IWP2 or 5C-treated hepatocytes. B) Bar-plot depicting the scaled activity scores of key Wnt, TGFβ and non-canonical Wnt transcription factors across freshly-isolated hepatocytes, "mock-" and IWP2-treated hepatocytes. C-D) Volcano plots depicting a repressive effect of IWP2 treatment on the target genes underlying key Wnt signaling transcription factors CTNNB1 (β-catenin; C) and TCF7L2 (D). Genes that are positively modulated by these TFs and suppressed upon IWP2 treatment are highlighted in blue.

Supplementary information on statistical tests

Each tab of the excel sheet presents a table of the statistical tests performed per figure with the associated P-values. ~~**Figure**~~

Formatted: Font: Not Bold

Commented [KA(1]: The main manuscript refers to this as Figure 5 while the Figure legend refers to this as Figure S6 (originally).

Change this to Figure 5 and move up the figure legend section.

Formatted: Font: (Default) +Body (Calibri)

Formatted: Default Paragraph Font, Font: Bold, Ligatures: Standard + Contextual

Formatted: Font: (Default) +Body (Calibri)
