# Supplementary material for: Inhibition of Wnt signaling in primary human hepatocytes promotes Plasmodium falciparum liver stage development
Source: PLoS Pathog. 2025 Dec 22;21(12):e1013800. doi: 10.1371/journal.ppat.1013800 (PMC12795456; doi:10.1371/journal.ppat.1013800)
Supplement: Attachment — Submitted filename: Response to the reviewers_final.docx [file ppat.1013800.s012.docx]

We thank all reviewers for their critical, but overall positive evaluation of our manuscript. Below please find our answers to their suggestions and our plan to revise the manuscript accordingly.

Reviewer #1 (Evidence, reproducibility and clarity (Required)):

Challenges associated with growing the liver stage of human malaria parasites in culture is a major roadblock to research. Primary human hepatocytes are currently the model of choice. This manuscript addresses the problem that these cells dedifferentiate quickly in culture, which correlates with a much-reduced ability to support parasite development. They use transcriptomics to describe the dedifferentiation process, conclude a potential role for Wnt signalling as a mediator and demonstrate that the Wnt inhibitor IWP2 preservers the ability of HPPs to support parasite growth over a longer period. They also use a Wnt activator, whose ability to reduce parasite development is less consistent.

Overall, this study provides an interesting chemical fix for increasing culture efficiency for P. falciparum, which labs working with this model may choose to adopt. However, the study does not go beyond presenting pharmacological evidence. Title and abstract claim that the work establishes a causal link between Wnt signalling, hepatocyte differentiation state and parasite development. It is my opinion that claims of a mechanism or causal link need to be supported by more than pharmacological evidence, and that in this case transcriptomics as the only way of assessing the state of the cells is insufficient.

Questions that come to mind are whether nuclear translocation of catenin really occurs in an inhibitor-sensitive way, whether specific known Wnt targets are regulated at the protein/biochemical pathway level and which of these specific targets/pathways are important for parasite development. However, I am no expert on the cell biology of Wnt signalling, neither am I aware of all the tools available.

We appreciate the reviewer’s concern about the chemical compounds (namely IWP2 and CHIR99021) and their specificity in targeting Wnt pathway. While we agree with the reviewer that in general chemical inhibition is not a full-proof approach on its own, considering the substantial amount of published literature and our transcriptomics assessment of the excerpted effects (see later), we are confident that IWP2 and CHIR99021 are modulating the Wnt pathway as we claimed by the targeting of key effectors of this pathway.

The original paper by Chen et al^1^ characterizing IWP2 as WNT inhibitor showed a decrease in B catenin level after 24 hours at 5uM (10 fold higher than what is used in this study). It also demonstrated IWP2 specificity by showing that overexpression of the compound target, Porcupine can restore the loss of wnt activity (measured using a Wnt/B catenin pathway responsive firefly luciferase reporter) at 0.25, 0.5 and 1uM treatment with IWP2. An extensive but not all-inclusive literature search (n= 9^1-9^ including the original Chen et al) showed the usage of IWP2 (0.1uM to 20uM from 24 hours to 5-6 days) on a variety of cell types (mostly cancerous cell-lines with the Wnt/B catenin pathway responsive firefly luciferase reporter) showing either a loss of B catenin and/or loss of luciferase activity due to lack of activation of the reporter construct. We picked a concentration of IWP2 that has been used and published in primary human hepatocytes^9^ as it was the most relevant cell type for this study.

With regards to the usage of CHIR99021, the original paper by Ring and colleagues^10^ showed that this compound exhibited 500 to >10000 fold selective for its target GSK3; being equally potent to both isoforms of the enzyme but concurrently able to discriminate between GSK and its closest homologues (cdc2 and erk2). There, the authors concluded that CHIR99021 was a highly potent and selective inhibitor of GSK3. Again, an extensive (but not all-inclusive^2,3,6,8,11-14^) literature search shows that CHIR99021 is used on a variety of cell types at a concentration of 2-3 uM (3uM was used in our manuscript). Of note, Lee et al^2^ and Arensmen et al^3^ have already shown the increase in B catenin levels (in their respective cells used) using western blot, immunofluorescence and/or the transfected Wnt/B catenin pathway responsive firefly luciferase reporter.

Notwithstanding the need for some clarification, as far as they go, the data linking pharmacological agents and parasite development appear sound and I would say the same for the transcriptomic analysis as such. My concern is that neither go deep enough to claim more than preliminary evidence for a link between hepatocyte Wnt signalling and parasite development. I could imagine this manuscript rewritten as a more technical report on how to improve P. falciparum liver stage culture in practice or as a starting point for a deeper study into mechanism. The latter would be needed to support the claims from title and abstract.

With all due respect to the reviewer, we feel that our manuscript goes way beyond a simple technical note. We would have loved to confirm our observations regarding WNT inhibition using genetic tools, but these are very challenging in this experimental system. Genetic deletion of the B-catenin (key component of the wnt signalling i.e. mimicking the IWP2 condition) in mice results in severe defect in hepatocyte maturation^15^, and therefore highly likely to prevent the study of Pf liver stage development. We showed previously that Pf sporozoites prefers mature hepatocytes for successful development and maturation^16^. Importantly, efficient overexpression or depletion of target proteins in primary human hepatocytes are non-existent technologies currently. Notwithstanding our conviction that genetic manipulation would yield very similar result, we will mention the potential limitation of chemical inhibitors in the manuscript and indicate the validation via genetic means is desired in the future.

Furthermore, we now also provide data demonstrating that Pf parasites do not show differences in size when grown in PHHs with pre-treatment with IWP2 or CHIR99021 but then returned to the standard growth media of WBH (Figure S5) i.e. the chemical (ant)agonists must be present during parasite development to have the indicated impact. This further cements the importance of Wnt pathway in determining parasite development.

Major points

The rapid dedifferentiation of primary human hepatocytes poses a challenge to their application in toxicology, studies of human liver functions and to develop 3D tissue culture models. How to maintain hepatic functions is a long-standing problem and previous attempts at solving it have included small molecules to maintain the cells differentiated state for longer (for instance PMID: 31432507). The author here use development of P. falciparum liver stages as readout, but there must be a broader context of published work from other uses of PHHs, which the authors should consider, including possibly transcriptomics. A quick search of GEO finds >2000 projects related to "primary human hepatocytes", for instance PMID: 36241695. The authors appear to be taking a rather narrow view of past work. Placing their data in a broader context would help the non-expert feel reassured they are not reinventing the wheel.

We are certainly agree with the reviewer that there are plentiful transcriptomic data available in the literature and might be relevant to use. However, with all honesty, we are unclear as to what the reviewer is proposing here. To the best of our knowledge there is no transcriptomic data available in the literature using these specific inhibitors on PHHs, but even if such data would be available, given potential donor and experimental condition-specific differences, we feel it is still valuable to repeat these experiments in the very same cell culture system where the infections are also performed.

What does it mean in terms of mechanism if Wnt signalling is associated with the transcriptomic signature of the cultured cells. Are genes of the signalling pathway enriched or beta-catenin targets, for instance?

The most likely effect of the repression of the signalling pathway is the downregulation of associated target genes and consequent alteration of the cellular state. To assess this we have reanalysed the RNA-seq data and investigated the relative activity of transcription factors (via quantifying the deregulation of their targets genes). As expected the Wnt-associated transcription factors (e.g. beta-catenin/CTNNB1, TFC7L2) displayed increased activity during *in vitro* culturing and most of their target genes displayed clear down-regulation upon IWP2 treatment (**Figure 2**). Next to these we also observed altered activity of the SMAD (TGFb) and AP1 (noncanonical WNT) transcription factors, which is likely due to indirect effect of WNT deregulation. This is in agreement with published literature stating the interlinked nature of the two pathways (WNT and TGFb). However, given the lack of impact of TGFb inhibitor (SB) alone on schizont size (Figure 2G of the manuscript), we are confident of the WNT pathway’s impact on parasite development.

***Figure 2****:* ***IWP2 treatment does supress expression of WNT associate transcription factor target genes****.* ***A)*** *Scaled activity scores for canonical Wnt/TGFβ signalling and non-canonical Wnt (AP1 family) transcription factors.* ***B)*** *Volcano plot depicting the deregulated expression of CTNNB1(beta-catenin) target genes in IWP2 treated cells compared to culture in B27 supplemented medium. Target genes which are stimulated by CTNNB1 and downregulated in IWP2 treatment (demonstrating at suppression of CTNNB1 activity) are highlighted in amber.*

Next to changes in WNT target gene expression IWP2 treatment also reduced the expression of core WNT pathway components (e.g. ligand WNT, receptor: Frizzled) providing further evidence for the effect of IWP2 on this pathway.

***Figure 3****: Schematic of elements of Wnt signalling pathway deregulated in IWP2-treated vs mock treated cultured PHHs. Overall decreased expression of several canon signalling elements is highlighted in green and upregulation of pathway inhibitors in red.*

I am not convinced from Fig. 3G that the treated cells are transcriptomically more like the starting condition than the mock treated cells. The statement in line 155 that "that IWP2 suppressed the transcriptional changes observed during the in vitro culture in B27" needs to be qualified and supported with data. The populations overlap on the PCA and both are clearly distinct from the starting condition. They go on to show through analysis of the transcriptomic data that differences between treated vs untreated cells are consistent with reduced Wnt signalling and less dedifferentiation. However, from the PCA plot this would appear to be on a background of much variation. Can the authors come up with a better measure of overall similary?

Based on spatial proximity in the PCA space, B27 cultured cells were distinct from fresh hepatocytes (Figure 3G in the manuscript). Upon IWP2 treatment we observed a notable shift of cultured samples towards fresh samples on the PCA space. To quantify this, we calculated the sample distance scores which further indicated increased similarity between IWP2-treated cells and fresh hepatocyte (mean distance score: 156) as compared to mock treatment (mean distance score: 137) (Note that lower distance scores define higher similarity between pairwise samples under comparison). Furthermore, treatments with IWP2 suppressed the deregulation of many genes that were otherwise upregulated during *in vitro* culture in B27 medium (**Figure 3**). We will include these extra information in the revised manuscript to further substantiate our conclusion.

***Figure 4:*** ***Upregulation of numerous genes upregulated during* in vitro *culture is effectively inhibited by IWP2 treatment.***

*Gene expression heatmap of fresh (brown) and in vitro cultured mock as well as IWP2 treated PHH samples (orange and grey, respectively). The gene set under investigation here is restricted to genes originally identified to be upregulated in in vitro cultured PHHs compared to fresh hepatocytes. As apparent from the heatmap, upregulation of these genes is efficiently suppressed by IWP2 treatment.*

Minor points:

Errors related to numerus are in the title and in lines 63, 126, 142, Fig. S1 legend.

Incomplete sentences or sentences needing rephrasing starting lines 65, 96, 147, 210, 224

Line 99: Why "return"? Use more precise language. Once gone, schizonts cannot be recreated.

Line 101: "to into"?

Line 116: "cannot fully prevent loss of hepatic functions"... Rephrase. Functions were not measured.

Line 153: reference to Fig. 4A must be 3G.

Line 193: How does Fig. S4 relate to "all these up-regulated pathways"? The authors should state which pathways and which of their data they mean. Is this about the inhibitor cocktail? What if its effect is off target and not due to the precise combined inhibition of ""all these up-regulated pathways"? There is not much one can conclude from an inhibitor cocktail or even a single pharmacological agent.

We have now added “Furthermore, 5C treatment appear to exert transcriptional and/or cellular changes beyond the prevention of EMT (data not shown).”

Fig. S1: Panel lettering in the figure and legend do not match.
In the legend, the sencence starting "As for..." is garbled. Also, what is "per replicate (2 replicates)" and how does it relate to there being more than 2 dots? What is ***?

We have created an excel sheet (Supplementary information) where all the statistical tests and their corresponding p values are listed per figure. We hope that creates more clarity.

Fig. S4: Rephrase "Data show mean" and provide a legend for A and B.

We thank the reviewer for all the minor points raised, which we will all effectively address in the revised manuscript.

**Referees cross-commenting**

The reviews do not strike me as incongruous, in that there is general agreement that the experimental work is sound and of practical utility for parasitologists studying Pf liver stages.

Whether one accepts pharmacological data as sufficient to claim a mechanistic insight is a matter of judgement. I would not be comfortable with this work entering the literature as evidence for a role for Wnt signalling in host-parasite interactions, as implied in the title because, a) it only investigates how to fix the problem with a culture system and b) it does so without the rigor human cell biologists can apply to questions of signal transduction and cellular differentiation.

I'd have no problem with this if the authors wanted to publish their work under the title "An inhibitor of Wnt signalling improves Pf development in primary human hepatocytes". The challenge I see is that journals using Review Commons tend to want mechanistic insight.

Reviewer #1 (Significance (Required)):

Time required for revision depends on whether the authors choose to revise their presentation and claims or provide deeper insights into mechanism of the pharmacological agent used.

Reviewer #2 (Evidence, reproducibility and clarity (Required)):

This paper reports on the role of cellular state of hepatocytes on their infectability by the human malaria parasite P. falciparum. The authors find that longer cultivation of primary hepatocytes leads to a drop in infectivity by parasites. Several gene regulatory networks were found to be associated to this phenomenon and blocking of some of them, most prominently wnt signalling affects the development of P. falciparum. The data are clear and statistically significant

The paper is well written and nearly ready to publish. I only have a few minor comments:

Line 57: rephrase Eph A2 or also cite the study calling this discovery into question
We will cite the study calling this discovery into question.

Figure 1:
Please be coherent in writing, e.g. schizonts vs Schizonts in Y-axis of panels
We will correct this inconsistency.

Figure panels 1F-H , 2A, 3G-J: not sure the grey bars on top and right of panel are needed. I would advise to delete

We are uncertain as to which grey bar the reviewer is referring to and these might only be visible after pdf conversion or in a specific computer environment. Would these still appear in the revised manuscript we will find a way to delete them.

Figure 5, should the label 'b-catenin' not be changed to 'b-catenin linked actin filaments' (or similar)?

We will change the “b-catenin” to “b-catenin linked actin filaments”.

The paper will be interested to researchers investigating the wnt signaling pathway (>42000 Pubmed entries on 'wnt signalling') and malaria liver stages (>2000 Pubmed entries). Hence interest should transcend the infectious disease community.
I am working on the cell biology of malaria parasites.

**Referees cross-commenting**

I agree with the note of reviewer 1.

Reviewer #2 (Significance (Required)):

in this paper the authors provide a well conducted study in the effects of de-differentiation of primary hepatocytes for their capacity to serve as host cells for P. falciparum, the parasite eliciting the most deadly form of human malaria. The parasite undergo a massive first expansion in the liver, which is clinically silent, before they burst out into the blood stream, where they cause the symptoms of this disease. The molecular details of parasite-host cell crosstalk in the liver are studied by only a few brave groups due to the fact that parasites need to be grown in mosquitoes in high security insectaries and primary liver cells are also not easy to get. The paper is the first to my knowledge that implements wnt signaling, an ancient signaling cascade, in the growth of liver stage parasites using a mix of infection studies and transcriptome analysis.
We thank the reviewer for his/her kind comments regarding the importance of finding Wnt signalling impact on the growth of liver stage parasites and its potential follow-on impact on other liver pathogens.

Reviewer #3 (Evidence, reproducibility and clarity (Required)):

In this study, the authors address the host-parasite interaction in the context of malaria liver stage and more precisely which host signaling pathways could be involved in the development of the intracellular Plasmodium parasite within human hepatocytes. To do so, they use an in vitro culture system relying on freshly isolated primary human hepatocytes (PHH) infected at different time points post-plating with Plasmodium falciparum sporozoites.

- for all the study they compare the susceptibility of PHH to different strains of P. falciparum (PfNF54, PfNF175 and PfNF135) which are known for displaying different levels of infection and growth kinetics in PHH;

- they use appropriate markers for estimating liver stage parasite maturation : the size of the intracellular schizont, the number of parasite nuclei contained in the schizont and the expression of a middle-late liver stage antigen, the MSP1 protein; for estimating hepatocyte quality they chose the number of nuclei as a surrogate for cell viability and human glutamine synthetase expression for quantitative analysis of hepatocyte health;
- as stated by the authors, it is indeed well known that PHH rapidly loose their phenotype after in vitro culture. Their first show that PHH susceptibility to P. falciparum (Pf) infection, regardless of the PHH batch and Pf strain, is severely diminished as soon as day 5-7 post-hepatocyte plating (p.p.) compared to day 2 and this is not due to a decrease in hepatocyte survival;

- then they identify a culture medium (referred as "B27") that improves infection levels and hepatocyte survival up to day 7 post-plating but still these two parameters dramatically decline after day 7 p.p..

- in order to identify the hepatocyte factors that would be responsible for this loss in differentiated hepatocyte phenotype and Pf susceptibility, the authors compared the transcriptomic profiles of uninfected PHH on the day of isolation (day 1) with day 7;
- their strategy then was to counterbalance the upregulation of some genes observed on day 7 to try to recover the phenotype of day 1 PHH. They thus identified 5 pathways (Wnt, TGF-b, Notch, BMP, Adenylyl-cyclase) and tested each using specific antagonists. Their data are consistent with those formerly published by Xiang et al (Nat Com 2019);
- The antagonists for the TGF-beta and Wnt pathways were the two showing the most significant effect on parasite's number, size and maturation (MSP1+), as well as hGS expression by infected cell. However the mix of the 5 molecules, previously described to promote functional maintenance of PHH in vitro together with VHB replication life cycle (Xiang et al, Nat Com 2019), did not improve susceptibility to Pf nor its development within treated PHH;

- Then, they confirmed that the transcriptome of PHH treated or not with Wnt antagonists are different and highlight genes and biological processes that are either upregulated or suppressed in Wnt antagonist treated cells;

- Finally, they confirm that when hepatocytes are treated with a Wnt activator then parasite number and size are decreased albeit to a lesser extent when compared to the opposite effect of Wnt inhibitor.

Major comments:

- Are the key conclusions convincing? Yes.

- Should the authors qualify some of their claims as preliminary or speculative, or remove them altogether? No, excepted maybe the Figure 5 which can be considered too much speculative.

Figure 5 provides mechanistic insight that and a potential explanation of how the molecular effectors of the Wnt pathway interact to regulate parasite growth and development. It is (what we feel) as a nice summary to end with.

- Would additional experiments be essential to support the claims of the paper? Request additional experiments only where necessary for the paper as it is, and do not ask authors to open new lines of experimentation.

The effect of the different treatments on hepatocyte health could be more documented (nuclei number shown for all tested experimental conditions and/or LDH quantification in the supernatant to assess for cell viability and/or albumin quantification in the supernatant.

We agree with the reviewer that such extra information could be useful, but we feel that it is not strictly essential to support our main conclusions as regards to parasite growth. Notably, providing such data would require repeating the experiments (since supernatants are not preserved), an effort, in our opinion, not proportional to the information gained. We will however, provide numbers of hepatocyte count as suggested below.

- Are the suggested experiments realistic in terms of time and resources? It would help if you could add an estimated cost and time investment for substantial experiments.
Measure of nuclei number should be accessible without the need for performing additional experiments as long as the authors stored the plates. Other experiments could be possible if the authors stored culture supernatants frozen; also both LDH and Albumin quantification are quite expensive.

We have analysed the existing data to show the number of hepatocytes per well as a measure of hepatocyte health. We will insert this appropriately in a supplementary figure. This graph has been now split into Figure S2, 3 and 4, depending on the treatment condition.

- Are the data and the methods presented in such a way that they can be reproduced?
Yes. The authors should specify how many sporozoites they inoculate to the hepatocyte cultures and how often they renew the culture medium.

We agree with the reviewer and will add these details to the methods section.

- Are the experiments adequately replicated and statistical analysis adequate?
The number of replicates allow to have confidence in the data. This represents a big amount of work given the nature of the study, e.g. working with freshly isolated PHH and mosquitoes infected with different strains of Pf. One of the challenge when working with fresh human hepatocytes is the variability from one batch to another. Here the authors provide data from three independent experiments per results which can be considered sufficiently robust and moreover they obtain consistent results for these different batches, which also consolidates the data.

We thank the reviewer for recognising the challenge with working with fresh human hepatocytes and with getting consistent data despite the donor-to-donor variations. Like you mentioned, the consistency achieved in this data set irrespective of the donor gave us great confidence (despite lack of genetic data) that the wnt pathway is involved in Pf liver stage development.

- Other major comment:

The authors should explain how they chose the antagonists or activator that they use and also to which extent they are specific for each pathway. They also should specify how they chose the concentration of each antagonist that they use. The authors should explain why they chose to treat PHH with Wnt antagonist for a duration of 6-8 days before performing infection with Pf parasites.

We apologise for lack of details in the antagonist and activator used in this paper. We will rectify in the introduction/discussion (see reviewer 1, point 1). Similarly, we will motivate in the result section why we treated for 6-8 days: based on Figure 1B, untreated PHH showed the lowest permissibility for parasite development (lowest number of schizonts) by day 7 post plating, therefore we wanted to make sure the treatment was robust enough to overcome this phenomenon. The range of treatment days were due to practical/logistic reasons such as when sporozoites or primary human hepatocytes are available.

Minor comments: We agree with the reviewer’s minor comments and will address accordingly.

- Specific experimental issues that are easily addressable. See above.
Host nuclei counts are now provided. (see Figure S2-4).

- Are prior studies referenced appropriately? Yes albeit the authors could have discussed their data in comparison with those of other transcriptomic studies on human malaria liver stage addressing the response of the hepatocyte to the infection.
The majority of these studies are performed in the rodent models of malaria and may not be translatable.

- Are the text and figures clear and accurate?

Yes but there are many sentences that could benefit from rewriting/reformulation to gain in clarity, mainly in the results section:

Lines 120-122: "Testing the potential relevance of specific signalling pathways in culture-induced upregulation of genes (Figure 1F) showed a strong enrichment of genes associated with Wnt signalling."

Line 134: "DAPT-treated hepatocytes contained parasites displayed limited, but significant.."

Lines 137-138: "Similarly, in IWP2-treated hepatocytes schizonts showed improvements especially in size ..."

Lines 147-148: "PHHs were treated with either IWP2 (6 days) prior to and during Pf infection"; the authors should remove "either" from the sentence

Line 195: "appear" should be corrected for "appears"

- Do you have suggestions that would help the authors improve the presentation of their data and conclusions?

No.


Reviewer #3 (Significance (Required)):

- Describe the nature and significance of the advance (e.g. conceptual, technical, clinical) for the field.

The nature of the advance is conceptual. This work provides interesting results regarding the biological pathways of the hepatocyte that are involved in the development of Plasmodium falciparum, a field for which very little is known. By highlighting the role of the Wnt pathway on the parasite development it provides new data to the field of research in malaria liver stage.

- Place the work in the context of the existing literature (provide references, where appropriate).
There is very few studies addressing the interactions between hepatocyte and Plasmodium falciparum during the liver stage.

- State what audience might be interested in and influenced by the reported findings.
This work will be beneficial for the community working on malaria liver stage, for basic research but could also provide new avenues of research aiming at identifying novel antimalarial therapies.

- Define your field of expertise with a few keywords to help the authors contextualize your point of view. Indicate if there are any parts of the paper that you do not have sufficient expertise to evaluate.

The key words describing my area of expertise are:

Malaria liver stage, Plasmodium species that infect humans, PHH, immunity, in vitro and in vivo models.

I do not have sufficient expertise in RNA seq analyses to evaluate this part of the work.

We thank the reviewer for his/her recognition of the strength of our dataset, for highlighting the lack of existing knowledge on Pf liver stage development and therefore the impact of our findings for the field.

1	Chen, B. *et al.* Small molecule-mediated disruption of Wnt-dependent signaling in tissue regeneration and cancer. *Nat Chem Biol* **5**, 100-107, doi:10.1038/nchembio.137 (2009).

2	Lee, H. J., Wolosin, J. M. & Chung, S. H. Divergent effects of Wnt/beta-catenin signaling modifiers on the preservation of human limbal epithelial progenitors according to culture condition. *Sci Rep* **7**, 15241, doi:10.1038/s41598-017-15454-x (2017).

3	Arensman, M. D. *et al.* WNT7B mediates autocrine Wnt/beta-catenin signaling and anchorage-independent growth in pancreatic adenocarcinoma. *Oncogene* **33**, 899-908, doi:10.1038/onc.2013.23 (2014).

4	Kleszcz, R., Szymanska, A., Krajka-Kuzniak, V., Baer-Dubowska, W. & Paluszczak, J. Inhibition of CBP/beta-catenin and porcupine attenuates Wnt signaling and induces apoptosis in head and neck carcinoma cells. *Cell Oncol (Dordr)* **42**, 505-520, doi:10.1007/s13402-019-00440-4 (2019).

5	Zhang, J. *et al.* DKK1 promotes migration and invasion of non-small cell lung cancer via beta-catenin signaling pathway. *Tumour Biol* **39**, 1010428317703820, doi:10.1177/1010428317703820 (2017).

6	Shin, Y. S. *et al.* The Wnt/beta-catenin signaling pathway plays a role in drug-induced liver injury by regulating cytochrome P450 2E1 expression. *Toxicol Res* **39**, 443-453, doi:10.1007/s43188-023-00180-6 (2023).

7	Janda, C. Y. *et al.* Surrogate Wnt agonists that phenocopy canonical Wnt and beta-catenin signalling. *Nature* **545**, 234-237, doi:10.1038/nature22306 (2017).

8	Xu, H. *et al.* Unfolded protein response is required for the definitive endodermal specification of mouse embryonic stem cells via Smad2 and beta-catenin signaling. *J Biol Chem* **289**, 26290-26301, doi:10.1074/jbc.M114.572560 (2014).

9	Xiang, C. *et al.* Long-term functional maintenance of primary human hepatocytes in vitro. *Science* **364**, 399-402, doi:10.1126/science.aau7307 (2019).

10	Ring, D. B. *et al.* Selective glycogen synthase kinase 3 inhibitors potentiate insulin activation of glucose transport and utilization in vitro and in vivo. *Diabetes* **52**, 588-595, doi:10.2337/diabetes.52.3.588 (2003).

11	Huang, J., Guo, X., Li, W. & Zhang, H. Activation of Wnt/beta-catenin signalling via GSK3 inhibitors direct differentiation of human adipose stem cells into functional hepatocytes. *Sci Rep* **7**, 40716, doi:10.1038/srep40716 (2017).

12	Touboul, T. *et al.* Stage-specific regulation of the WNT/beta-catenin pathway enhances differentiation of hESCs into hepatocytes. *J Hepatol* **64**, 1315-1326, doi:10.1016/j.jhep.2016.02.028 (2016).

13	Ma, Y. *et al.* CHIR-99021 regulates mitochondrial remodelling via beta-catenin signalling and miRNA expression during endodermal differentiation. *J Cell Sci* **132**, doi:10.1242/jcs.229948 (2019).

14	Jin, C., Samuelson, L., Cui, C. B., Sun, Y. & Gerber, D. A. MAPK/ERK and Wnt/beta-Catenin pathways are synergistically involved in proliferation of Sca-1 positive hepatic progenitor cells. *Biochem Biophys Res Commun* **409**, 803-807, doi:10.1016/j.bbrc.2011.05.094 (2011).

15	Tan, X. *et al.* Beta-catenin deletion in hepatoblasts disrupts hepatic morphogenesis and survival during mouse development. *Hepatology* **47**, 1667-1679, doi:10.1002/hep.22225 (2008).

16	Yang, A. S. P. *et al.* Development of Plasmodium falciparum liver-stages in hepatocytes derived from human fetal liver organoid cultures. *Nat Commun* **14**, 4631, doi:10.1038/s41467-023-40298-7 (2023).
