# Supplementary material for: Inhibition of Wnt signaling in primary human hepatocytes promotes Plasmodium falciparum liver stage development
Source: PLoS Pathog. 2025 Dec 22;21(12):e1013800. doi: 10.1371/journal.ppat.1013800 (PMC12795456; doi:10.1371/journal.ppat.1013800)
Supplement: Attachment — Submitted filename: Response_to_the_reviewers_final_auresp_2.docx [file ppat.1013800.s014.docx]

We would like to thank all three reviewers for their appraisal of our manuscript and comments. Below please find our response to their comments and suggestions:

**Part I – Summary**

Reviewer #1: This paper addresses an important problem - the low efficacy of hepatocyte infection by Plasmodium falciparum and finds a way to improve it. Along this way the authors find two signaling pathways that are playing a role in improved infection. The problem is that these pathways are activated in vivo anyway and thus the paper gives no real insights into a biological problem.

Reviewer #2: This is a revised version of a manuscript where the authors explore the mechanisms by which primary human hepatocytes rapidly lose their ability to support P. falciparum (Pf) liver stage development in culture conditions.

The authors used transcriptomics and identified several pathways potentially involved in the loss of function of PHH cultures, including Wnt. They demonstrate that the Wnt inhibitor IWP2 results in much improved schizont growth and maturation in vitro. In contrast, a Wnt activator has only a modest effect. Importantly, the effect of IWP2 is confirmed using different hepatocyte donors and with three different Pf strains. The manuscript is clearly written and the work is well performed.

The authors addressed in part the comments of the previous reviewers. The authors are to be commended for this extensive work that tackles a major roadblock in malaria research. The work will be extremely useful for researchers working on Pf liver stage biology. However, the biological impact of the data, beyond technical improvement of the culture conditions, is much less clear to me.

We are happy to see that both reviewers recognise the value of our manuscript in highlighting the relevance of certain host signalling pathways and the technical advancements they bring about. With this technical advancement, we have drastically extended the period (from two days post plating to 14 days) in which PHHs can be used for Pf infection after culture, offering much needed flexibility for the researcher. The reviewers however are wondering about the direct relevance of these findings to the *in vivo* situation. This is indeed difficult to assess given the inaccessible nature of human livers *in vivo* for research purposes. Therefore, *in vitro* models of hepatocytes are the only readily available host source in our arsenal to perform research on Pf liver stage biology, currently. Regardless, the pathways demonstrated in our manuscript are known and important host pathways to the field of hepatology and are involved in many aspects of liver biology and therefore offering a glimpse of the *in vivo* situation. However, we understand the reviewers point and have toned down our statements regarding the specific pathways and rather focus on the cellular changes they bring about and consequent impact of parasite development.

Reviewer #3: I have made numerous and extensive edits to the PDF I was sent to review and the editors and authors are welcome to read them and I feel the authors should address them to improve the paper.

Ultimately, I believe that the study is fascinating but lacks scientific rigor and the results section is poorly written which made it challenging for me to fully digest the importance of the work.

We thank this reviewer for his/her positive opinion about our study and the extensive editing of the manuscript which we have taken maximally into consideration during this revision.

**Part II – Major Issues: Key Experiments Required for Acceptance**

Reviewer #1: please change the title, this is misleading as you don't provide a role for wnt signaling in vivo but in vitro. I think the paper is a great and helpful contribution but please don't make it sounds like it's more than it is. Adapt the abstract accordingly, ie tone it down somewhat

We have toned down our statement regarding the wnt pathway in particular and focus on the general cellular changes it brings about. We have also changed the title of the manuscript to accommodate this critique: “ Inhibition of Wnt signaling in primary human hepatocytes promotes *Plasmodium falciparum* liver stage development”.

Reviewer #2: In culture, PHH rapidly undergo epithelial-mesenchymal transition (EMT) associated with loss of hepatic functions, resulting in poor Pf susceptibility. The main claim of the paper is that Pf parasites depend on Wnt pathway regulation for efficient liver stage development. The claim is based on the observation that pharmacological inhibition of Wnt results in highly improved liver stage development. This effect is clearly documented throughout the manuscript. However, it is unclear whether this is a direct effect of the Wnt pathway on parasite development or merely the result of preserved hepatic functions due to inhibition of EMT. Some elements are missing in the demonstration to help the reader distinguish between direct or indirect effects. In particular, in Fig 1, a comparison of the size of the schizonts when infection is performed in fresh (day 2) versus plated (Day 5-7) PHH in the different conditions (with or without IWP2) would be helpful to distinguish whether IWP2 preserves or promotes liver stage development.

IWP2 treatment can also promote parasite growth via maintaining certain cellular features of the host hepatocytes: this we cannot rule out and we have now more clearly mentioned this in the manuscript. However, our data does imply a “direct” impact of Wnt pathway on parasite development. Due to the complexity of these infection assays, we do not have corresponding fresh PHHs infected with the different Pf strains in the absence or presence of IWP2. However, we have now plotted this to historical published data (Yang 2021 EMBOJ) which shows the size of the parasites in freshly cultured PHHs (without IWP2). This is comparable to the sizes observed in PHHs cultured for 5-7 days in WBH media (prior and during infection) for all three parasite strains (Figure 1). This combined with the reduction of schizont size down to WBH levels upon removal of IWP2 from the medium during infection (Figure S5) are all suggestive of the Wnt pathway having a direct impact on schizont size and maturation.

A stronger demonstration of the role of the Wnt pathway during infection would be to use a model where hepatocytes do not dedifferentiate, such as humanized mouse models. In this regard, how do day 5 schizonts in IWP2-treated PHH cultures compare in terms of size with schizonts in liver-humanized mice? Another way to look at this would be to test the effect of CHIR99021 on freshly isolated PHH.

We thank the reviewer for these suggestions. Unfortunately, testing the impact of Wnt pathway in vivo is far from trivial. Direct comparison of the size of schizont on PHHs and humanised mice is unlikely to be conclusive in this regard. Next to this while humanised mice is a great model in many ways but to our knowledge the zonation pattern which is mainly regulated by the wnt pattern is “messed up” in their reconstitutes liver, severely complicating conclusive experiment. To make these experiments somewhat conclusive one would need to genetically manipulate the wnt pathway in vivo, which is challenging enough *in vitro* let alone *in vivo*.

Testing the effect of CHIR99021 on freshly isolated PHH from these mice is a possibility but one must realise that as soon as PHHs are removed from the segment and plated, the process of de-differentiation begins.

In the absence of in-depth characterization of the Wnt pathway I agree with Reviewer 1 that the manuscript should rather be presented as a technical report investigating the mean to improve the Pf liver stage culture system.

We respectfully disagree that our manuscript should be presented as technical note, but did tone down our statements regarding the wnt pathway.

Fig 1B shows a drop in the number of schizonts when the age of PHH culture increases, pointing at a defect in invasion and/or survival of the parasite. While IWP2 does not seem to overtly affect the number of schizonts, this is an important feature of the PHH culture ageing. B27 medium clearly preserves the number of schizonts, at least for 1 week. Did the authors check for expression of entry/survival factors ? There are clearly two distinct features of PHH that seem to be regulated differently (at least in part), i.e. susceptibility to infection versus ability to promote parasite maturation, and this should be at least discussed in the manuscript.

We have investigated the expression of four host encoded *Plasmodium* receptors (SR-B1, CD81, CD36 and EPHA2) across the culture conditions. We can corroborate dramatic drop in expression of all of these receptors in *in vitro* cultured (B27-mock) hepatocytes compared to freshly isolated primary hepatocytes. This likely explains the drop in parasite invasion rates over extended culture duration.

IWP2 treatment had minor significant improvement on SR-B1 expression and a minor non-significant improvement to CD81 expression. IWP2 treatment resulted in no change to expression of CD36 and suppressed EPHA2 expression (both being ancillary invasion receptors).

Additionally we noted the significant drop in expression of AQP3 during upon vitro culture (compared to fresh). AQP3 is a host factor co-opted by Pf for supporting its intra-hepatic growth. IWP2 treatment resulted in a minor but non-significant increase in expression of this host factor. This could in part explain the better parasite growth in IWP2 treated hepatocytes.

Is it possible that IWP2 has a direct effect on the parasite? Stopping treatment when sporozoites are added to the culture results in the drop of the effect (Fig S5, IWP2* condition). What happens in the reciprocal condition (addition of IWP2 only with the parasite)? Is there an improvement of parasite maturation?

This is an interesting question and appears to relate to the point raised above. While we cannot exclude the possibility for a direct effect of IWP2 on parasite development we do not have any reasons to suspect it either for the following reasons: i) we do show that IWP2 does have a clear effect on the cellular and transcriptional state of the host cell, which is likely influences parasite development; ii) Since activation of the Wnt pathway via and agonist leads to the opposite effect on parasite development, it appears more likely that the modulation of the host cell rather than the direct effect of IWP2 would be responsible for the increase growth phenotype; iii) IWP2 is a very specific inhibitor of porcupine a protein only present in multicellular animals; iv) would IWP2 have any direct effect on the parasite, it would most likely be a negative effect and we have a hard time imagining how would it promote parasite growth.

Unfortunately, due to the obligate relationship between the parasite and host cell it is impossible the differentiate between the direct and indirect effect, which we now briefly mention in the manuscript.

Reviewer #3: I have made numerous and extensive edits to the PDF I was sent to review and the editors and authors are welcome to read them and I feel the authors should address them to improve the paper.

In one of the key experiments, outlined in Figure 4 and highlighted in my edited PDF, media composition was changed and the reason was not explained. I believe this experiment should be repeated.

We believe there is a misunderstanding: the media composition was not changed. IWP2 (Wnt inhibitor) was replaced with CHIR99021 (Wnt activator) because we would like to further conclude the impact of the Wnt pathway on determining Pf liver stage schizont development. Since we showed that inhibition of the Wnt pathway through IWP2 enhances liver stage schizont size and maturation, it is logical that activating the same pathway will lead to the opposite effect.

No discussion is given as to why three strains of Pf were used in the study.

These parasite strains have distinct (*in vitro*) growth characteristics and hence to show that the effect of the tested inhibitors is robust across the strains we included multiple strains. We wanted to identify a host pathway that is influential for multiple Pf strains. This is now briefly explained in the manuscript.

**Part III – Minor Issues: Editorial and Data Presentation Modifications**

We thank the reviewers for spotting these minor issues, which we have corrected.

Reviewer #1: small errors should be corrected such as

115: delete 'into'

146: rephrase 'neither / nor'

156: add 'signaling' after wnt and tgf-b

159: add 'pathway' after wnt

Reviewer #2: The authors use either “permissiveness” or “permissibility”. This could be homogenized.

Line 115: to into

Line 131. It would be logical in the reading flow to include parasite maturation (size) in Fig 1.

Line 150-157. The text should refer clearly to numbers versus size of the schizonts.

Line 174-175. Lower scores define higher similarity between samples. Then please check the numbers (IWP2: 168; B27: 147).

The 5C cocktail seems to prevent EMT, yet has not such a positive effect on Pf infection in vitro. This raises the possibility that one of the regulated pathways could be required for parasite maturation. Did the authors try different combinations of the inhibitors to search for the most suitable Pf-adapted cocktail?

We did try some but not all combinations of individual inhibitors, but found that IWP2 on its own had the most prominent effect of parasite growth.

Discussion line 257-263. The observation that Pf prefers zone 3 hepatocytes, where Wnt signaling is more prominent, seems to contradict the in vitro data and the claim of the manuscript. Could the authors clarify this discrepancy?

While it is true that Pf prefers zone 3 hepatocytes where Wnt signaling is more prominent, the main conclusion from Yang et al 2021 EMBOJ was that the parasites prefer these cells due to the presence of human glutamine synthetase which is imported into the developing parasites and make no claims regarding Wnt signalling. While Wnt is also important for the establishment of zonality in the liver it is by far not the only factor contributing the establishment of the specific cellular and tissue environment “preferred” by the parasite.

Reviewer #3: I have made numerous and extensive edits to the PDF I was sent to review and the editors and authors are welcome to read them and I feel the authors should address them to improve the paper.
