# Supplementary material for: Inhibition of Wnt signaling in primary human hepatocytes promotes Plasmodium falciparum liver stage development
Source: PLoS Pathog. 2025 Dec 22;21(12):e1013800. doi: 10.1371/journal.ppat.1013800 (PMC12795456; doi:10.1371/journal.ppat.1013800)
Supplement: Attachment — Submitted filename: Kanyal_20250919_clean_rev.docx [file ppat.1013800.s013.docx]

Inhibition of Wnt signaling in primary human hepatocytes promotes *Plasmodium falciparum* liver stage development

Abhishek Kanyal^1^*, Geert-Jan van Gemert^2^*, Haoyu Wu^1,3^*, Alex van der Starre^2^, Johannes HW de Wilt^4^, Teun Bousema^2^, Robert W. Sauerwein^2,^, Richárd Bártfai^1^*, Annie SP Yang^2,5,6^*

*these authors contributed equally

1. Department of Molecular Biology, Faculty of Science, Radboud Institute for Molecular Life Science, Radboud University, Nijmegen, The Netherlands

2. Radboud Center of Infectious Diseases, Department of Medical Microbiology, Radboud University Medical Center, Nijmegen, the Netherlands

3. CAS Key Laboratory of Regenerative Biology, Guangzhou Institutes of Biomedicine and Health, Chinese Academy of Sciences, Guangzhou 510530, China

4. Department of Surgery, Radboud University Medical Center, Nijmegen, the Netherlands

5. Present address: Leiden University Center for Infectious Diseases (LUCID), Leiden University Medical Center, Leiden, the Netherlands

6. Lead contact.

Correspondence: a.s.yang@lumc.nl (ASPY) and r.bartfai@science.ru.nl (RB)

**Abstract**

After infection of the human host, the first stage of the *Plasmodium falciparum* (Pf) lifecycle takes place in the liver. However, understanding host-parasite interactions during liver stage development is limited by the rapid loss of functionality when cultured primary human hepatocytes (PHHs) are used for liver stage propagation. Here, we link loss of hepatic functionality to the loss in Pf permissiveness, which we prevent by using a medium containing serum-replacement and signal transduction inhibitors. Integrating transcriptomic analysis and phenotypic assessment of infection outcome, we identified several host signaling pathways that influence Pf liver stage development. Specifically, inhibition of the Wnt pathway had a major impact on the size and maturity of Pf liver stage schizonts, by retaining the metabolic activity and epithelial nature of PHHs. Understanding how the alteration of PHH cellular state via signalling pathways determines Pf liver stage permissiveness provides insight into host-parasite interactions and could accelerate development of novel therapeutic strategies via improved *in vitro* liver stage models.

**Authors’ summary**

Despite substantial elimination and control measures malaria still causes mortality and global disease burden. Liver stage development of the malaria parasite is a critical first step preceding clinical symptoms and is the prime target of antimalarial vaccine/drug development. However, we lack sufficient understanding of critical components of the host hepatocyte environment supporting or preventing liver stage parasite development. In this study, we observed substantial decline in the ability of cultured primary human hepatocytes to support parasite development and identified molecular signatures and altered activity of host signaling pathways associated with this loss of permissiveness. Specifically, we demonstrate the impact of the Wnt pathway on the growth and maturation of liver stage parasites in cultured primary human hepatocytes. We thus provide insight into how host hepatocyte homeostasis can impact liver stage parasite development and pave the way for further dissection of host pathogen interactions and development of improved intervention strategies.

**Introduction**

The mosquito-borne disease, malaria, remains a devastating global burden with approximately 250 million cases and 620,000 deaths annually [1]. Most of the deaths are due to the parasite *Plasmodium falciparum* (Pf), which establishes itself inside hepatocytes as a liver stage after the initial deposition of sporozoites into the host skin by an infected mosquito. Over a period of approximately one week, the intracellular liver stage parasite replicates and differentiates into a mature schizont, that contains approximately 90,000 exoerythrocytic merozoites capable of invading red blood cells (RBCs) [2]. The release of these merozoites into the circulation and their subsequent cyclical asexual replication in RBCs is responsible for all clinical symptoms associated with the disease.

Despite its fundamental importance in enabling effective infection, our understanding of the complex parasite-host interactions within the infected hepatocyte remain elusive. Studies with the rodent malaria model *Plasmodium berghei* (Pb) model have been informative as a proxy for Pf but findings are often not translatable to Pf-host interactions: the rodent parasite has only a 2-day liver stage maturation time compared to 7-days for Pf [3]. Specific host membrane receptors have been identified to be involved in Pb sporozoite invasion of hepatocytes such as CD81 and Scavenger Receptor B1 (SR-B1) [4-6]; similarly, host factors involved in nutrient acquisition [7-10] and/or prevention of host cell death pathways [11-14].

Research in Pf liver stage development primarily involves *in vivo* models such as mice with humanised liver [2, 15, 16], or *in vitro* models where hepatocyte cell-lines such as HC-04 [17, 18] and primary human hepatocytes (PHHs) are used. The use of cell lines has been limited to the identification of host receptors involved in parasite invasion such as EphA2 [4, 19] and glypican 3 [18]. PHHs are considered the gold standard in drug assays [3, 20] and were used to identify host factors needed for the development of Pf liver stage schizonts such as SR-B1 [10, 21, 22] and glutamine synthetase [23]. A well-known phenomenon of *in vitro* cultured PHHs, however, is the quick loss of hepatic features [24, 25], yet the impact of this has not been explored in Pf liver stages development and may hinder the discovery of host factors that are important for full maturation of liver stage schizonts. Establishment of an *in vitro* hepatocyte model that preserves hepatocyte features is therefore needed. The loss of functional hepatic features is controlled by specific host signaling pathways [24] of which, their potential impact on parasite development have not been previously examined.

Here, we set out to 1) identify the impact of loss of hepatic functions in *in vitro* PHHs and their permissiveness to Pf infection, 2) improve and stabilize hepatocyte culture conditions for Pf permissiveness, facilitating complete schizont development and 3) identify host pathways in hepatocytes that influence the developmental kinetics of Pf liver stages. Using RNA sequencing to compare the transcriptomic profiles between freshly isolated and cultured PHH, we identified upregulation of the Wnt (Wingless and Int 1), TGF-β (Transforming Growth Factor Beta), Notch and Rho signaling pathways. Treatment with specific chemical antagonists of some pathways led to an improvement in Pf schizont development. Furthermore, the Wnt pathway was shown to be most influential in determining the size and maturation of the Pf liver stage schizont.

**Results:**

***In vitro cultured PHHs lose permissibility for Pf liver stage infection and development***

Traditionally fresh PHHs are cultured in standard medium containing human sera (William’s B with Human sera; WBH) [23, 26, 27] and infected with Pf 48 hours after plating. However, PHHs quickly lose their cellular identity, and this may impact permissibility to Pf infection. To investigate the effect of culturing time, we infected PHHs at different time points post-plating (p.p.) with Pf strains, chosen for their differences in developmental kinetics (PfNF54, PfNF175 and PfNF135 [23]). Schizont numbers were evaluated at five days post-infection (p.i.) (Figure 1A). We observed a sharp decrease in number of schizonts in relation to the p.p. period for all Pf strains tested (n = 2 for PfNF54 and PfNF135; n =1 for PfNF175) with the most drastic decline occurring between days 2 and 5 p.p. This reduction in the number of infected cells could not be explained by a decrease in the number of viable hepatocytes, which became prominent only after day 9 p.p. (Figure 1C).

A previous study showed that medium containing human sera affects hepatocyte functionality [21]. Therefore, we examined the impact of sera on hepatocyte permissiveness for liver stage development via the removal of sera (WB versus WBH) (Figure S1). Non-significant improvements in schizont numbers and the presence of the maturation marker PfMSP1 were observed in sera negative medium (WB). We hence concluded that a novel hepatic culture medium is needed to restore schizont numbers to those seen on day 2 p.p. A serum replacement supplement (B27) on a William’s E Glutamax base was tested given its beneficial effect for the differentiation of stem cells [28] and hepatic organoids into hepatocytes [29, 30] and other hepatic culture systems [24]. Addition of B27 to the basal medium William’s E Glutamax substantially delayed the loss of hepatocyte permissiveness by at least 7 days (Figure 1D). This was not due to changes in hepatocyte numbers which remained comparable to the WBH medium thus reflecting a change in the phenotype of the PHHs (Figure 1E).

To understand the cellular changes in PHHs that may influence Pf permissiveness and intracellular development, we compared the transcriptional profile of freshly isolated PHHs (n = 2) with uninfected PHHs cultured in B27 for 7 days p.p. This time point was selected since at this point the PHHs display a maximal reduction to Pf permissiveness. The transcriptional profile showed an upregulation of 2420 gene transcripts and a down regulation of 2756 genes (Figure 1F; cultured versus fresh). The most significantly up-regulated genes were those involved in actin filament organization, cell-substrate adhesions, wound healing and extracellular matrix organization (Figure 1G). All these pathways are classically associated with the epithelial to mesenchymal transition (EMT) which is a sign of trans-differentiation [3]. For down-regulated genes, we found a strong enrichment for those involved in metabolism i.e., fatty acid metabolism, lipid catabolism, organic anion transport and alcohol metabolism, among others (Figure 1H). These pathways are linked with metabolic functionalities of hepatocytes, and their downregulation indicates a decay of core hepatic traits. This could additionally alter the amount and type of metabolites available for the liver stage parasite. Together, while serum replacement with B27 had a substantial positive effect on PHH permissiveness to Pf infection, the transcriptional profile observed indicates significant differences to freshly isolated PHHs, pointing to a loss of hepatic functions which could affect liver stage size and maturation.

***Specific host signal transduction pathways influence Pf liver stage development***

To narrow down the regulatory processes that could lead to the observed gene expression changes we filtered our gene ontology enrichment analysis (for genes upregulated in cultured PHH) for hits against signalling pathway terms. Genes upregulated in cultured PHHs (Figure 2A) showed a strong enrichment for signaling pathways via Wnt (Wingless and Int-1), Ras, transforming growth factor beta (TGF-β), apoptosis and Notch. These pathways were chosen for further investigation due to the availability of commercial agonists and antagonists, their published role in hepatocyte functionalities and the significance of their up-regulation [24]. Compared to the fresh PHH, genes involved in the Wnt, TGF-β and Notch pathways were strongly upregulated (Figure 2B-D) in cultured PHH, while Bone Morphogenic Protein (BMP) and adenylyl-cyclase pathways were mildly upregulated (Figure 2E). While some donor-to-donor variation in the deregulation of individual genes was observed, a general up-regulation for all pathways was apparent (Figure 2B-D). To examine the impact of these host pathways on Pf liver stage development, we treated PHH for 6-8 days p.p., with selective agonists and antagonists of the individual pathways. Wnt signalling was inhibited with IWP2, TGFβ signalling was inhibited with SB431542, Notch signalling was inhibited with DAPT, BMP signalling was inhibited with LDN193189 and adenylyl-cyclase signaling was activated with Forskolin (Figure 2F-J). Quantitative measurements assessed the replication (size, nuclear DNA content via DAPI; Figure 2G, H), health (human glutamine synthetase: hGS; Figure S2B-C) and maturation (PfMSP1; Figure 2I) status of the liver stage schizonts, respectively. Modulation of both the BMP and the adenylyl-cyclase pathways through the usage of LDN193189 and Forskolin, respectively did not result in significant improvements in any of the parameters tested. DAPT-treated hepatocytes showed significant improvements in the number and size of schizonts observed but not in other parasite phenotypic parameters. Improvements were seen for SB431542 treatment for all five parameters most notably in the number of schizonts and expression of the maturation marker, PfMSP1. Similarly, IWP2-treated hepatocytes showed significant improvements in the schizont size, DNA content per schizont and expression of PfMSP1 per schizont but no significant increase in schizont numbers. Finally, we tested a previously published cocktail containing all five of these inhibitors (5C) [24] (Figure S3). Hepatocyte monolayers cultured in the presence of 5C showed poor support of PfNF175 schizont size and numbers (Figure S4D-J) contrary to its beneficial effect on viral infection reported earlier [24]. Together, these data demonstrated that inhibition of specific host pathways, and particularly Wnt (inhibited by IWP2), TGF-β (inhibited by SB431542) signalling are important for supporting Pf liver stage permissiveness and schizont development. Here we chose to further focus on the effect of the Wnt pathway as it had a more profound effect of parasite growth.

***Inhibition of the Wnt signalling pathway preserves the metabolic profile and epithelial nature of PHHs and improves the developmental kinetics of Pf liver stage development***

To study the earliest impact of Wnt pathway suppression on Pf liver stage development, PHHs were treated with IWP2 6 days prior to and during Pf infection (Figure 3A-F). IWP2 treatment promoted parasite development (size, nuclear content and PfMSP1 expression), from day 5 p.i. onwards for three Pf strains tested. To further capture the transcriptional changes associated with IWP2 treatment, RNA sequencing was performed on hepatocytes cultured in B27 (“mock”) or treated with B27 plus IWP2 at day 5 p.i. Furthermore, we also analysed the transcriptional changes brought about by 5C to understand why treatment with this cocktail was not beneficial for parasite growth. The original experiment was performed with five replicates per culture/treatment condition, but upon closer inspection we excluded replicate 2 from our analysis owing to suboptimal quality (Figure S6A). Based on spatial proximity in the PCA space, B27 cultured cells were very distinct from freshly isolated hepatocytes (67% of variance in the PC1 axis, Figure 3G) as also evident from Figure 1F-H. Upon IWP2 treatment we observed a notable shift of B27 cultured samples towards fresh samples on the PCA space although it remained closer to B27 than to fresh (Figure 3G). To quantify this shift, we calculated the sample distance scores which indicated an increased similarity between IWP2-treated cells and fresh hepatocyte (mean distance score: 147) as compared to B27 cultured cells (mean distance score: 168) (lower scores define higher similarity between samples). Interestingly, 5C-treatment elicited additional transcriptional changes as indicated by an increased separation of the samples away from fresh hepatocytes along the PC2 axis (Figure 3G). In IWP2-treated cells, 394 genes were upregulated, and 344 genes downregulated relative to B27 cultured (“mock”) cells, (Figure 3H). Gene Ontology analysis of the genes upregulated in IWP2 treated cells revealed enrichment of metabolism associated terms including fatty acid, alcohol and steroid metabolism (Figure 3I). Among the biological processes suppressed upon IWP2 treatment, we found enrichment of extra-cellular matrix organization, surface-adhesion, epithelium migration and response to TGF-β and Wnt-signaling (Figure 3J). This indicates that IWP2 treatment is able to partially revert the deregulation of key biological pathways observed in PHHs during culture. IWP2 treatment mainly suppressed genes (252 genes) that are otherwise upregulated during *in vitro* culture in B27 medium (as described above, Figure 1G, H). We, hence, conclude that IWP2 treatment improves the metabolic profile of cultured hepatocytes by suppressing the epithelium to mesenchymal transition, and is associated with an improved developmental kinetics of liver schizonts.

***The activation status of the Wnt pathway determines the size of Pf liver stage schizonts***

We subsequently projected the gene-expression changes induced by the IWP2 treatment on elements of the Wnt signaling pathway and identified a modulation of core signaling components (e.g. suppressed expression of ligand: WNT; receptor: Frizzled; transcription factor: TCF/LEF) (Figure 3L). The most likely effect of the repression of the signaling pathway is the downregulation of associated target genes and consequently, an alteration of the cellular state. To assess this, we investigated the activity of key transcription factors (via quantifying the deregulation of their targets genes). As expected, the Wnt-associated transcription factors (e.g., beta-catenin/CTNNB1, TFC7L2) displayed increased activity during *in vitro* culturing and most of their target genes displayed clear down-regulation upon IWP2 treatment (Figure S6B-D). Next to these we also observed altered activity of the SMAD (TGF-β) and AP1 (noncanonical WNT) transcription factors as inferred from deregulation of their target genes, which is likely due to an indirect effect of WNT deregulation (Figure S6B). This agrees with published literature that shows the interlinked nature of the WNT and TGF-β pathways [31-33]. However, given the lack of impact of the TGF-β inhibitor (SB431542) alone on schizont size (Figure 2G), we are confident of the WNT pathway’s impact on parasite development.

To further examine the impact of the Wnt pathway on Pf schizont size, we treated PHHs with the well-known Wnt activator CHIR99021 prior and during PfNF135 and PfNF175 infection, given that inhibitor of Wnt (IWP2) showed enhanced schizont size and maturation (Figure 4A-D). PfNF54 in this context was not tested due to lack of sporozoite availability. Compared to the B27 control, the number of schizonts were not altered after CHIR99021 treatment but (Figure S4A-B) schizonts in CHIR99021-treated PHHs were significantly smaller than B27 cultured schizonts on day 5 p.i. for both Pf strains. There was also a significant size decrease for PfNF135 (Figure 4A) but not for PfNF175 (Figure 4B) at day 7 which may be the result of arrested development of PfNF175 under B27 conditions. CHIR99021 treatment also resulted in a relative decrease in PfMSP1 expression significant for PfNF175 on day 7 p.i. Furthermore, pretreatment of hepatocytes with IWP2 or CHIR99021 was followed by return to WBH after NF175 infection, schizont features (i.e. size, DAPI content and PfMSP1 level) were comparable to schizonts observed in hepatocytes cultured in WBH the entire time. WBH was chosen as opposed to B27, it was obvious that this media was insufficient to support full schizont development. Thus, both inhibition and activation of the Wnt pathway in hepatocytes affects predominantly schizont size and maturation. Collectively, this demonstrates the influence of the host Wnt signaling pathway on schizont development for two parasite strains, PfNF135 and Pf175, in PHHs.

**Discussion**

In this study, we show that PHHs in *in vitro* culture quickly lose their ability to sustain a Pf liver stage infection, irrespective of the parasite strain. This loss of permissibility coincides with the up-regulation of specific signaling pathways in PHHs. Inhibition of individual up-regulated pathways (Wnt, TGF-β, and Notch) results in enhanced or maintained hepatocyte permissibility leading to an improvement in schizont development. In addition, we identify the Wnt-pathway as an important host factor regulating cellular features of the host hepatocyte important for liver stage maturation.

The combined repression of four of the up-regulated pathways (Wnt, TGF-β, Notch and BMP) with an adenylate cyclase activator (Forskolin) i.e. the 5C cocktail greatly benefits hepatitis B virus infection as shown previously [19]. However, this is detrimental to hepatocyte permissiveness and development of Pf as shown in this study (Figure S4). This could be due to the differing intracellular host requirements by the two distinct pathogens: for example, the intrahepatic growth of HBV is 72 hours compared to the 7 days of Pf. Furthermore, 5C treatment exerted transcriptional and/or cellular changes beyond the prevention of EMT (Figure S6). In case of Pf, we show that specific individual host pathways rather than the combination of all pathways bring strong improvement on parasite development.

The inhibition of the Wnt pathway in cultured hepatocytes has a marked effect on parasite size and maturation. This increase in parasite size is concordant with the upregulation of genes involved in lipid, alcohol and steroid metabolism (Figure 4). While general metabolic health of the hepatocyte is conceivably beneficial for parasite growth, fatty acid synthesis is relevant to parasite development and formation of hepatic merozoites [34]. Furthermore, upon Wnt inhibition we find a downregulation of genes typical for the EMT. In the context of *in vitro* cultures, the EMT is mainly due to the loss of tissue environment. EMT however can also occur *in vivo* during liver fibrosis [35] and might influence liver stage infection.

While the most logical explanation the effect of IWP2 is via the modulation of the signalling pathway and cellular state of the host hepatocyte, we cannot exclude the possibility for a direct effect of IWP2 on parasite development. However, we have no reasons to suspect it to be the case for the following: i) we show that IWP2 has a clear effect on the cellular and transcriptional state of the host cell, which likely influences parasite development; ii) since activation of the Wnt pathway via an agonist leads to the opposite effect on parasite development, it appears more likely that the modulation of the host cell rather than the direct effect of IWP2 would be responsible for the increase growth phenotype; iii) IWP2 is a specific inhibitor of porcupine, a protein only present in multicellular animals [36].

While our study highlights the relevance of the inhibition of the Wnt signaling pathway in *in vitro* cultures, this observation might also be relevant during *in vivo* zonal differentiation of hepatocytes. It is well established that within the liver lobule hepatocytes display marked differences in gene expression and metabolism along the portal central axis (also referred to as zonation, [37]) and components of the Wnt pathway are predominantly present in zone 3 hepatocytes and heavily involved in maintaining hepatic zonation [38]. We have previously shown that Pf parasites strongly prefer zone 3 (pericentral) hepatocytes resulting in better parasite development [23], as similarly shown for Pb [39]. While zone 3 hepatocytes possess all components of the Wnt pathway i.e., the surface receptor and intracellular signaling components, it is possible that external Wnt ligand/signaling proteins may be secreted by hepatocytes from neighbouring zones or other non-hepatocyte cells present in the liver [40] and may therefore also play a role in determining schizont size.

Inhibition of the TGF-β (SB431542), Notch (DAPT), BMP (LDN193189) and the activation of the adenylate cyclase (Forskolin) pathways all showed improved schizont size compared to B27. This could be attributed to their dynamic interaction with the Wnt signaling pathway. In forskolin treated schizonts, activated adenylate cyclase leads to increased levels of cAMP which in turn can activate the Wnt pathway [41, 42]. The relationship between the BMP pathway (LDN193189) and the Wnt pathway is less obvious as the former can either inhibit or activate the Wnt pathway depending on the presence of wildtype of p53 or the loss of SMAD4 respectively [43]. This may explain the large variation observed in schizonts grown with LDN193189 as it is dependent on the donor status. Inhibition of the Notch pathway (by DAPT) maintains PHHs in their hepatocyte lineage (as opposed to a more bile ductal phenotype) as well as indirectly inhibiting the Wnt pathway [44]. This could hence explain the significant increase in schizonts grown in DAPT-treated PHHs compared to the control B27 medium. Finally, TGF-β signaling leads to the activation of the Wnt pathway [45] therefore its inhibition results in schizonts with increased size, nuclear content, and maturation.

Despite their differences in infectivity and phenotype, schizonts of all tested Pf strains are significantly larger and more mature in IWP2-treated PHHs than their counterparts in B27-treated PHHs. This larger size becomes significant during the process of schizont growth, i.e., after day 3 p.i. and becomes prominent from day 5 p.i. onwards; blockage of Wnt communication between infected- and uninfected cell could allow for better expansion of the growing schizont with less of host restriction. It has been estimated that an infected hepatocyte can expand up to 200 times its original volume to accommodate the growing Pf schizont [46]. This must necessitate communication between the infected and surrounding uninfected hepatocyte and the Wnt signaling pathway may serve here as communication platform [33,35-37]. IWP2 specifically inhibits the enzyme porcupine (Porcn), which is involved in transport of the wnt ligands [47]. One could hypothesize that neighbouring hepatocytes keep their strict size and volume via the steady basal level of wnt signaling and thereby limiting the growth of Pf schizonts [48]. The above hypothesis is summarised in Figure 5 which shows a potential mechanism of action of Wnt in determining schizont size.

In conclusion, we show that PHHs cultured in standard medium (WBH) rapidly lose their permissiveness to Pf liver stage development. This loss, however, can be substantially delayed by the usage of a defined serum replacement (B27) on top of a William’s E Glutamax base. Furthermore, we identify three specific host signaling pathways (Wnt, TGF-β and Notch) to modulate Pf liver stage development. Inhibition of Wnt signaling controls the size of the developing Pf schizont and is therefore a key factor in determining Pf developmental kinetics for multiple parasite strains. Transcriptome analysis of Wnt inhibitor treated PHHs indicates that improvement of hepatic metabolism, prevention of EMT and cell division lead to the production of more mature liver stage parasites. Together, these findings provide steps forward in our understanding of the intricate interaction between hepatocyte and liver stage parasite, unlocking rational approaches for future therapeutic interventions.

**Materials and Methods**

**Ethics**

Primary human liver cells were freshly isolated from remnants of surgical material. The samples were anonymised and general approval for their use was granted in accordance with the Dutch ethical legislation as described in the Medical Research (Human Subjects) Act. It was confirmed by the Committee on Research involving Human Subjects in the region of Arnhem-Nijmegen, the Netherlands. Approval for use of remnant, anonymized surgical material for transcriptome analysis was specifically confirmed by the Committee on Research involving Human Subjects, in the region of Arnhem-Nijmegen, the Netherlands (CMO-2019-5908).

**PHH isolation from liver segments**

Primary human hepatocytes were isolated from patients undergoing elective partial hepatectomy as previously described [23, 27]. Freshly isolated hepatocytes are suspended in complete William’s B medium (WB): William’s E with Glutamax (ThermoFisher Scientific: 32551087), 1x Insulin/transferrin/selenium (ThermoFisher Scientific: 41400045), 1mM sodium pyruvate (ThermoFisher Scientific: 11360070), 1x MEM Non-essential amino acid solution (ThermoFisher Scientific: 11140035), 100 units/ml Penicillin-Streptomycin (ThermoFisher Scientific: 15140122) and 1.6μM of dexamethasone (Sigma Aldrich: D4902). PHHs were plated at 62,500 cells per well in 96 well format and kept in a 37°C (5% CO_2_) incubator with daily medium changes 96-well plates (Falcon: 353219) precoated with Type 1 collagen solution from rat tail (Sigma Aldrich: C3867).

**Generation of sporozoites for liver infection**

Pf asexual and sexual stages were cultured in a semi-automatic system as described [49-51]. *Anopheles stephensi* mosquitoes were reared at the Radboud University Medical Center Insectary (Nijmegen, the Netherlands) in accordance with standard operating procedures. Salivary glands from infected mosquitoes (day 14 to day 23 post infection) were hand-dissected and collected in WB medium. Collected glands were homogenized using home-made glass grinders and sporozoites were counted in a Burker-Turk chamber using phase-contrast microscopes. Immediately prior to infection of human hepatocytes, the sporozoites were supplemented with heat-inactivated human sera (HIHS) at 10% of the total volume (i.e. WBH). The multiplicity of infection is 0.8 (e.g., 50,000 sporozoites to 62,500 hepatocytes) or 1 (62,500 sporozoites to 62,500 hepatocytes) depending on the yield of the sporozoite batches. The MOI is the same within an experiment/biological replicate but may differ between experiments (as it depended on the sporozoite yield of the dissected mosquitoes.

**Medium compositions and duration**

*Different plating time after isolation (Figure 1B, C)*: Isolated PHH were plated in WB medium. The following day, it was changed to WBH: this medium was refreshed daily until the end of the experiment on day 26 post plating. In figure 1D and E, isolated PHH were again plated in WB medium. The following day, it was changed to William’s E with Glutamax (ThermoFisher Scientific: 32551087) supplemented with 1x B27 Supplement (ThermoFisher Scientific: 17504044) and 100 units/ml Penicillin-Streptomycin (ThermoFisher Scientific: 15140122) which was referred to as B27 medium or mock in the article. On the day of infection, B27 medium was removed and replaced with sporozoites suspended in WBH for three hours. After the infection process has occurred (i.e. after three hours), the WBH (sporozoite) medium was replaced with B27 medium until the conclusion of the experiment.

*Different medium treatments:* Isolated PHH were plated in WB medium. The following day, it was changed to the following medium conditions: B27 or B27 supplemented with 20μM of Forskolin (Enzo Life Sciences: BML-CN100-0010), or 10μM of SB431542 (Tocris: 1614), or 0.5μM of IWP2 (Tocris: 3533), or 5μM of DAPT (Tocris: 2634) or 0.1μM of LDN193189 (Tocris: 6053) or the combination of all the compound inhibitors (i.e. 5C). The hepatocytes were kept on this treatment for another 6 days (i.e. 7 days post plating) and then infected with sporozoites where the medium composition changes to WBH for three hours. After three hours, the monolayers were returned to their respective treatments. Media was refreshed on a daily (24hour) basis.

*Investigating the Wnt pathway:* Isolated PHH were plated in WB medium. The following day, it was changed to the following medium conditions: B27 or B27 supplemented with 0.5μM of IWP2 or 3μM CHIR-99021 (Sigma-Aldrich: SML1046). The hepatocytes were kept on this treatment for another 6 days (i.e. 7 days post plating) and then infected with sporozoites where the medium composition changes to WBH for three hours. After three hours, the monolayers were returned to their respective treatments. Media was refreshed on a daily (24hour) basis.

**Immunofluorescence readout**

Monolayers were fixed with 4% paraformaldehyde (ThermoFisher Scientific: 28906) for 10 minutes and permeabilised using 1% Triton for 5 minutes. The samples are stained with the various primary Pf or human antibodies: Rabbit PfHSP70 at 1:75 dilution (StressMarq Biosciences: SPC186), Mouse PfMSP1 at 1:100 dilution (Sanaria and NIH/NIAD: AD233), and mouse human glutamine synthetase at 1:100-250 dilution (Abcam: ab64613). Secondary antibodies were used at these following dilutions: Goat anti-rabbit Alexa Fluor 594 at 1:200 dilution (ThermoFisher Scientific: A11012) and goat anti-mouse Alexa Fluor 488 at 1:200 dilution (ThermoFisher Scientific: A11029). DAPI was used at 300 nM to stain the nuclear material of the monolayer.

**Microscopy**

The Zeiss LSM880 with Airyscan at 63x objectives (oil) and 2x zoom were used for detailed images. For high content images, the Zeiss Axio Observer Inverted Microscope Platform with AI assisted experimental startup was used. The images were acquired at 20x objectives with a numerical aperture of 0.8.

**Data analysis using FIJI**

*Infection rate:* For each well, 77 images were acquired in a tiled format. Approximately half (i.e. 39) images were counted on FIJI [52] for NF135 and NF175 infections. All the tiles were counted for NF54 due to the lower infection rate. The number of hepatic nuclei were counted for 1% of the total image (i.e. 7-10 images) and then extrapolated to get number of PHHs per well.

*Measurement of schizont size:* See Yang et al for further details [23]. Images obtained on the high content microscope were opened in FIJI. Random images were chosen until at least 100 schizonts were measured (per well) unless the infection is with NF54 (at least 50 schizonts). Schizonts were selected using the region of interest (ROI) tool based on PfHSP70 positivity (red channel) and measured. This ROI mask is applied onto the other colour channels i.e. blue and green to obtain values of nuclear content (DAPI) and hGS or PfMSP1 signal. For hGS and PfMSP1, background non-specific staining was considered and subtracted from the final signal. Further details regarding the methods can be found in [21, 23].

**RNA Isolation and RNA sequencing library preparation**

Freshly isolated or in vitro cultured hepatocytes (mock or treatment) were homogenized in TRIzol solution and cryopreserved at -80°C. RNA was subsequently isolated using the Zymo research Direct zol RNA purification kit (Cat. No. #R2053). The isolated RNA was assessed for quality and quantified using Nanodrop and agarose gel electrophoresis. RNA sequencing libraries from isolated/purified RNA were prepared using the Kapa mRNA Hyperprep Kit (Cat. No. #KK8581) using slight modifications of the manufacturer’s instructions for mRNA enrichment method. The input total RNA for the various libraries was selected between 150ng, 250ng or 500ng. The enriched mRNA was fragmented at 94°C for 6min and dA tailing performed at 55°C for 15min. In order to better capture the AT rich *Pf* transcriptome, we modified the PCR protocol as follows: Initial denaturation at 98°C for 2min; Cycling denaturation at 98°C for 20sec, annealing + extension at 62°C for 2min; final extension at 62°C for 3min. We selected PCR cycles for library amplification (12, 11 and 10 respectively) based on starting input total RNA amount. The amplified libraries were quantified using Denovix dsDNA high sensitivity kit (Cat. No. #TN145)on a Qubit fluorometer. Further qualitative assessment of library size distribution was performed using the Agilent high sensitivity DNA kit (Cat. No. #5067-4626) on the Agilent 2100 bioanalyzer platform. Each library was sequenced in 42bp paired-end format for roughly 18 million reads on the Illumina Nextseq 500 platform.

**RNA sequencing data analysis**

The sequencing endline fastq files were quality checked using FASTQC (ver. 0.11.9). The reads were subsequently trimmed for quality (-q 30) and adapter removal using the Trim_Galore software (ver. 0.6.7). The trimmed reads were aligned onto the *Homo sapiens* ver. 38 genome using STAR aligner (ver. 2.7.10a). The reads mapped onto the respective genomes were counted using the –quantMode GeneCounts option in STAR. Reads mapping only to sense strand were corrected for batch effects stemming from different hepatocyte donors using the Combat-seq tool (sva package ver. 3.44.0). The batch corrected counts were used for subsequent differential expression analysis (for conditions/treatments) in DESEq2 package (ver. 1.36.0) in RStudio (ver. 4.2.2 “Innocent and Trusting”). PCA plot for the various sequenced libraries/samples was generated using plotPCA command. Volcano plots for differentially expressed genes and genes of interest were generated using custom scripts and ggplot2 (ver. 3.4.2) in R. Gene Ontology enrichment analysis was performed in using the clusterProfiler tool (ver. 4.4.4) with subsequent dot-plots generated using inbuilt functions in ggplot2. Heatmaps for differentially expressed genes were generated using the Morpheus online tool by Broad Institute and custom scripts in R (ggplot2). KEGG pathway schematic for Wnt signaling was generated using the pathview tool package (ver. 1.46.0) in R. Transcription factor activity analysis was performed using the decoupleR package (ver. 2.6.0 utilizing collecTRI database) from the Julio Saez-Rodriguez lab [53, 54]. Bar-plot visualization for TF activity scores was done in GraphPad Prism 8.

**Statistical analysis**

For the majority of the experiments, at least three biological replicates were performed with two technical replicates. All statistical tests were performed using Prism 10. See figure legends for details regarding the statistical tests.

**Data availability**

The next-generation sequencing dataset associated with this study has been uploaded to Gene Expression Omnibus: GSE263643

The dataset comprises of:

The metadata sheet with information on experiments and samples

Raw data files: fastq files for RNA-sequencing

Processed data files: i) raw counts files generated from Combat-seq and ii) normalized count files generated from DESeq2

**Author contributions:**

AK, GJG, HW, AS, and ASPY performed the experiments. JHWdW coordinated the collection of the fresh human liver segments. ASPY and AK performed the analysis on the data. TB was involved in the writing and reading of the manuscript. AK, HW, RWS, RB and ASPY were involved in the conceptualization and writing of the manuscript.

**Acknowledgements:**

We are grateful for R. Stoter, W. Graumans, M. Vegte-Bolmer, A. Pouwelsen, L. Pelser-Posthumus and J. Kuhnen of the Malaria and Insectary Unit at the Radboud University Medical Center for parasite, mosquito and sporozoite production. We would like to thank the Microscopic Imaging Center (MIC) of the Radboud University for access to its facilities. A.S.P.Y was a recipient of the Veni grant from the Dutch Research Council (NWO) talent program (VI.Veni.192.171) and ZonMW Off-Road grant (04510012010050). A.K was supported by the Dutch Research Council (ZonMW-TOP-grant #91218010).

**Financial Disclosure:**

The salary of A.S.P.Y was supported by the Dutch Research Council (NWO) talent program (VI.Veni.192.171) and ZonMW Off-Road grant. A.K. was supported by the Dutch Research Council (ZonMW-TOP-grant #91218010). However, the funders had no role in study design, data collection, analysis, decision to publish or preparation of the manuscript.

References:

1.	WHO, W.H.O., *World Malaria Report 2022*. 2022.

2.	Vaughan, A.M., et al., *Complete Plasmodium falciparum liver-stage development in liver-chimeric mice.* J Clin Invest, 2012. **122**(10): p. 3618-28.

3.	Mo, A.X. and G. McGugan, *Understanding the Liver-Stage Biology of Malaria Parasites: Insights to Enable and Accelerate the Development of a Highly Efficacious Vaccine.* Am J Trop Med Hyg, 2018. **99**(4): p. 827-832.

4.	Langlois, A.C., et al., *Plasmodium sporozoites can invade hepatocytic cells independently of the Ephrin receptor A2.* PLoS One, 2018. **13**(7): p. e0200032.

5.	Silvie, O., et al., *Expression of human CD81 differently affects host cell susceptibility to malaria sporozoites depending on the Plasmodium species.* Cell Microbiol, 2006. **8**(7): p. 1134-46.

6.	Silvie, O., et al., *Hepatocyte CD81 is required for Plasmodium falciparum and Plasmodium yoelii sporozoite infectivity.* Nat Med, 2003. **9**(1): p. 93-6.

7.	Mancio-Silva, L., et al., *Nutrient sensing modulates malaria parasite virulence.* Nature, 2017. **547**(7662): p. 213-216.

8.	Lahree, A., J. Mello-Vieira, and M.M. Mota, *The nutrient games - Plasmodium metabolism during hepatic development.* Trends Parasitol, 2023. **39**(6): p. 445-460.

9.	Posfai, D., et al., *Plasmodium parasite exploits host aquaporin-3 during liver stage malaria infection.* PLoS Pathog, 2018. **14**(5): p. e1007057.

10.	Rodrigues, C.D., et al., *Host scavenger receptor SR-BI plays a dual role in the establishment of malaria parasite liver infection.* Cell Host Microbe, 2008. **4**(3): p. 271-82.

11.	Real, E., et al., *Plasmodium UIS3 sequesters host LC3 to avoid elimination by autophagy in hepatocytes.* Nat Microbiol, 2018. **3**(1): p. 17-25.

12.	Thieleke-Matos, C., et al., *Host cell autophagy contributes to Plasmodium liver development.* Cell Microbiol, 2016. **18**(3): p. 437-50.

13.	van de Sand, C., et al., *The liver stage of Plasmodium berghei inhibits host cell apoptosis.* Mol Microbiol, 2005. **58**(3): p. 731-42.

14.	Ebert, G., et al., *Targeting the Extrinsic Pathway of Hepatocyte Apoptosis Promotes Clearance of Plasmodium Liver Infection.* Cell Rep, 2020. **30**(13): p. 4343-4354 e4.

15.	Soulard, V., et al., *Plasmodium falciparum full life cycle and Plasmodium ovale liver stages in humanized mice.* Nat Commun, 2015. **6**: p. 7690.

16.	Sacci, J.B., Jr., et al., *Plasmodium falciparum infection and exoerythrocytic development in mice with chimeric human livers.* Int J Parasitol, 2006. **36**(3): p. 353-60.

17.	Sattabongkot, J., et al., *Establishment of a human hepatocyte line that supports in vitro development of the exo-erythrocytic stages of the malaria parasites Plasmodium falciparum and P. vivax.* Am J Trop Med Hyg, 2006. **74**(5): p. 708-15.

18.	Tweedell, R.E., et al., *The Selection of a Hepatocyte Cell Line Susceptible to Plasmodium falciparum Sporozoite Invasion That Is Associated With Expression of Glypican-3.* Front Microbiol, 2019. **10**: p. 127.

19.	Kaushansky, A., et al., *Malaria parasites target the hepatocyte receptor EphA2 for successful host infection.* Science, 2015. **350**(6264): p. 1089-92.

20.	Roth, A., et al., *A comprehensive model for assessment of liver stage therapies targeting Plasmodium vivax and Plasmodium falciparum.* Nat Commun, 2018. **9**(1): p. 1837.

21.	Yang, A.S.P., et al., *Development of Plasmodium falciparum liver-stages in hepatocytes derived from human fetal liver organoid cultures.* Nat Commun, 2023. **14**(1): p. 4631.

22.	Yalaoui, S., et al., *Scavenger receptor BI boosts hepatocyte permissiveness to Plasmodium infection.* Cell Host Microbe, 2008. **4**(3): p. 283-92.

23.	Yang, A.S.P., et al., *Zonal human hepatocytes are differentially permissive to Plasmodium falciparum malaria parasites.* EMBO J, 2021. **40**(6): p. e106583.

24.	Xiang, C., et al., *Long-term functional maintenance of primary human hepatocytes in vitro.* Science, 2019. **364**(6438): p. 399-402.

25.	Gomez-Lechon, M.J., et al., *Competency of different cell models to predict human hepatotoxic drugs.* Expert Opin Drug Metab Toxicol, 2014. **10**(11): p. 1553-68.

26.	Mazier, D., et al., *Complete development of hepatic stages of Plasmodium falciparum in vitro.* Science, 1985. **227**(4685): p. 440-2.

27.	Walk, J., et al., *Modest heterologous protection after Plasmodium falciparum sporozoite immunization: a double-blind randomized controlled clinical trial.* BMC Med, 2017. **15**(1): p. 168.

28.	Mallanna, S.K. and S.A. Duncan, *Differentiation of hepatocytes from pluripotent stem cells.* Curr Protoc Stem Cell Biol, 2013. **26**: p. 1G 4 1-1G 4 13.

29.	Huch, M., et al., *Long-term culture of genome-stable bipotent stem cells from adult human liver.* Cell, 2015. **160**(1-2): p. 299-312.

30.	Hu, H., et al., *Long-Term Expansion of Functional Mouse and Human Hepatocytes as 3D Organoids.* Cell, 2018. **175**(6): p. 1591-1606 e19.

31.	Guo, X. and X.-F. Wang, *Signaling cross-talk between TGF-β/BMP and other pathways.* Cell research, 2009. **19**(1): p. 71-88.

32.	Luo, K., *Signaling cross talk between TGF-β/Smad and other signaling pathways.* Cold Spring Harbor perspectives in biology, 2017. **9**(1): p. a022137.

33.	Guo, X., et al., *Axin and GSK3-β control Smad3 protein stability and modulate TGF-β signaling.* Genes & development, 2008. **22**(1): p. 106-120.

34.	van Schaijk, B.C., et al., *Type II fatty acid biosynthesis is essential for Plasmodium falciparum sporozoite development in the midgut of Anopheles mosquitoes.* Eukaryot Cell, 2014. **13**(5): p. 550-9.

35.	Fabris, L., et al., *Revisiting Epithelial-to-Mesenchymal Transition in Liver Fibrosis: Clues for a Better Understanding of the "Reactive" Biliary Epithelial Phenotype.* Stem Cells Int, 2016. **2016**: p. 2953727.

36.	Chen, B., et al., *Small molecule-mediated disruption of Wnt-dependent signaling in tissue regeneration and cancer.* Nat Chem Biol, 2009. **5**(2): p. 100-7.

37.	Gebhardt, R., *Metabolic zonation of the liver: regulation and implications for liver function.* Pharmacol Ther, 1992. **53**(3): p. 275-354.

38.	Russell, J.O. and S.P. Monga, *Wnt/beta-Catenin Signaling in Liver Development, Homeostasis, and Pathobiology.* Annu Rev Pathol, 2018. **13**: p. 351-378.

39.	Afriat, A., et al., *A spatiotemporally resolved single-cell atlas of the Plasmodium liver stage.* Nature, 2022. **611**(7936): p. 563-569.

40.	Zeng, G., et al., *Wnt'er in liver: Expression of Wnt and frizzled genes in mouse.* Hepatology, 2007. **45**(1): p. 195-204.

41.	Suzuki, A., et al., *PTH/cAMP/PKA signaling facilitates canonical Wnt signaling via inactivation of glycogen synthase kinase-3beta in osteoblastic Saos-2 cells.* J Cell Biochem, 2008. **104**(1): p. 304-17.

42.	Guo, R., et al., *Targeting Adenylate Cyclase Family: New Concept of Targeted Cancer Therapy.* Front Oncol, 2022. **12**: p. 829212.

43.	Voorneveld, P.W., et al., *The BMP pathway either enhances or inhibits the Wnt pathway depending on the SMAD4 and p53 status in CRC.* Br J Cancer, 2015. **112**(1): p. 122-30.

44.	Geisler, F. and M. Strazzabosco, *Emerging roles of Notch signaling in liver disease.* Hepatology, 2015. **61**(1): p. 382-92.

45.	Bogaerts, E., et al., *The roles of transforming growth factor-beta, Wnt, Notch and hypoxia on liver progenitor cells in primary liver tumours (Review).* Int J Oncol, 2014. **44**(4): p. 1015-22.

46.	Vaughan, A.M. and S.H.I. Kappe, *Malaria Parasite Liver Infection and Exoerythrocytic Biology.* Cold Spring Harb Perspect Med, 2017. **7**(6).

47.	Wang, X., et al., *The development of highly potent inhibitors for porcupine.* J Med Chem, 2013. **56**(6): p. 2700-4.

48.	Acebron, S.P., et al., *Mitotic wnt signaling promotes protein stabilization and regulates cell size.* Mol Cell, 2014. **54**(4): p. 663-74.

49.	Ifediba, T. and J.P. Vanderberg, *Complete in vitro maturation of Plasmodium falciparum gametocytes.* Nature, 1981. **294**(5839): p. 364-6.

50.	Ponnudurai, T., et al., *Cultivation of fertile Plasmodium falciparum gametocytes in semi-automated systems. 1. Static cultures.* Trans R Soc Trop Med Hyg, 1982. **76**(6): p. 812-8.

51.	Ponnudurai, T., et al., *Infectivity of cultured Plasmodium falciparum gametocytes to mosquitoes.* Parasitology, 1989. **98 Pt 2**: p. 165-73.

52.	Schindelin, J., et al., *Fiji: an open-source platform for biological-image analysis.* Nat Methods, 2012. **9**(7): p. 676-82.

53.	Müller-Dott, S., et al., *Expanding the coverage of regulons from high-confidence prior knowledge for accurate estimation of transcription factor activities.* Nucleic Acids Research, 2023. **51**(20): p. 10934-10949.

54.	Badia-i-Mompel, P., et al., *decoupleR: ensemble of computational methods to infer biological activities from omics data.* Bioinformatics advances, 2022. **2**(1): p. vbac016.

**Figure 1:** ***Pf-*schizont development post-hepatocyte plating and transcriptomic profiles of primary human hepatocytes (PHH).** A) The solid arrows show the number of days after liver isolation (post-plating) and the lines with the clear triangles show the period of schizont development. B) The number of schizonts in PHH on day 5 post infection (p.i.) when infected on different days p.p. for parasite strains: NF175 (magenta;), NF135 (green;) and NF54 (blue) in two independent experiments (except NF175). C) The number of PHHs present on day 5 p.i. on indicated days p.p for the specific parasite strains. For each independent experiment, the mean of three replicates is shown along with the standard deviation. The number of schizonts (D) and hepatocytes (E) on day 5 post invasion (p.i.) for NF135 and NF54 (n=1) for different infection times post plating (p.p.) cultured in B27 medium (grey). (F) Volcano plot highlighting the differentially expressed genes between hepatocytes cultured in B27 medium (“mock”) and freshly isolated hepatocytes (“fresh”). Dot-plots depicting the biological processes and upregulated (G) and downregulated (H) in cultured hepatocytes as compared to freshly-isolated hepatocytes.

**Figure 2:** **Effect of signaling pathways on Pf schizont development.** A) Dot-plot depicting the signaling pathways exhibiting higher activity in *in vitro* cultured hepatocytes as compared to freshly isolated hepatocytes. Heatmaps representing normalized expression of genes from (B)Wnt, (C)TGFβ, (D)Notch, (E)BMP and Adenylate cyclase signaling pathways deregulated in cultured vs freshly isolated hepatocytes. The data is scaled and representative of 2 biological replicates of RNA sequencing. The effect of specific host pathway inhibitors (IWP2, SB431542, DAPT, LDN193189 and Forskolin) on respectively schizont numbers (F; each dot is the average count of two technical replicates), size of schizonts (G; each dot is the median of at least 100 schizonts), total DAPI per schizont (H; each dot is the median of at least 100 schizonts), and total PfMSP1 per schizont (I; each dot is the median of at least 100 schizonts) on day 5 p.i.. For each graph, the treatments were displayed from highest differences from B27 treatment condition (left to right). The grey dotted line (per graphs F-I) shows the median measurements of schizonts grown in B27 control (from four independent experiments). The p-values from a Dunnett’s multiple comparisons test are shown – see supplementary information for full p values. J) Representative confocal images (from four independent experiments) showing schizonts grown in B27 alone and B27 supplemented with Forskolin, SB431542, IWP2, DAPT and LDN193189 on day 5 p.i. stained with PfMSP1, PfHSP70 and DAPI. Scale bar is 25 microns.

**Figure 3:** **Effect of IWP2 on developmental kinetics of NF175 (A-B). NF135 (C-D) and NF54 (E-F) schizonts.** For each Pf strain, the number schizonts, the size of schizonts, the total DAPI content and total PfMSP1 content are shown from left to right. The schizonts are either grown in hepatocytes cultured in either B27 (grey) or B27 supplemented with IWP2 (orange) for 6 days prior to infection and during schizont development on days 3, 5 and 7 p.i. Note briefly that day 3 schizonts of NF175 was not examined. For the number of schizonts, the mean of three independent experiments, each with 2-3 internal replicates is shown. For the schizont size and the total DAPI per schizont, at least 100 schizonts per each of three independent experiments (with two replicates per experiment) were measured. The mean of the median from each experiment is plotted and the graph shows the mean with standard deviation. For the total MSP1 content, at least 100 schizonts per each of three independent experiments were measured. The median is plotted, and the graph shows the mean with standard deviation. A Dunnett’s multiple comparisons test is performed: see supplementary information for full p values. B, D, F) Representative confocal images (from three independent experiments) showing NF175, NF135 and NF54 schizonts grown in B27 alone and B27 supplemented with IWP2 on day 7 p.i. stained with MSP1, HSP70 and DAPI respectively. Scale bar is 25 microns. (G) PCA plot highlighting the unique transcriptional states of freshly isolated (“fresh”), cultured (“mock”), IWP2 or 5C-treated hepatocytes. H) Volcano plot highlighting differential gene expression between IWP2-treatmentand ”mock”-cultured hepatocytes. Dot-plots depicting the biological processes upregulated (I) or downregulated (J) in IWP2-treated as compared to “mock” cultured hepatocytes. K) Gene expression heatmap highlighting the genes upregulated in mock cultured hepatocytes and suppressed upon IWP2 treatment. The expression counts have been normalized and scaled for plotting. L) Schematic overview of key genes involved in the Wnt signaling pathway that are modulated by IWP2 treatment. The Fold-change (log2) for the genes are scaled and plotted in blue-red color schematic.

**Figure 4: The impact of the Wnt signaling pathway on schizont size.** Hepatocytes treated with B27 or B27 supplemented with IWP2/CHIR99021 and infected with either NF175 (A) or NF135 (B). Schizont size (left), and total DAPI (middle) content are measured on days 5 and 7 post infection. The total amount of PfMSP1 per schizont was measured on day 7 p.i. (right). Each dot represents a schizont with at least 100 schizonts measured for three independent experiments. The median is shown, and a Dunn’s multiple comparisons test is performed with the p values displayed: see supplementary information for full p values. Representative confocal images (from three independent experiments) showing NF175 (C) and NF135 (D) schizonts B27 or B27 supplemented with IWP2/CHIR99021 on day 7 p.i. stained with MSP1, HSP70 and DAPI. Scale bar is 25 microns.

**Figure 5: Schematic of a possible mechanism in which the Wnt pathway controls Pf schizont development.** A) Under untreated and uninfected condition, there is a steady level of beta-catenin (β-catenin). In addition to its role in gene transcription, β-catenin is the intracellular component that links the cadherin adhesion molecules to actin filaments and therefore controls the “rigidity” of the cells in relation to its neighbours. B) In an infected hepatocyte (after 3 days post infection), the growing Pf schizont takes up so much of the hepatocyte volume that the trafficking of the Wnt ligands to the surface is disrupted. Wnt receptor of the neighbouring uninfected cells are not activated, and β-catenin molecules are degraded due to the phosphorylation of the enzyme, glycogen synthase kinase 3 (GSK3): this allows some flexibility in the uninfected neighbouring cells to accommodate the growth of the infected cell. However, in these (uninfected cells), Wnt ligands are still present and can interact with the existing Wnt receptors on the infected hepatocytes to maintain some β-catenin in the infected cell, thus limiting the growth/size of the schizont. C) Under IWP2 treatment, Wnt ligands are not trafficked to the surface of both uninfected and infected hepatocytes due to the inhibition of the enzyme porcupine (target of IWP2). Porcupine “labels” (via palmitoylation) Wnt ligands for correct trafficking to the plasma membrane. As a result, Wnt receptors on both uninfected and infected cells are not activated and existing β-catenin are degraded leading to reduced connection between cadherin and actin filaments (i.e. cell-cell contacts) and ultimately reducing rigidity. D) Under CHIR99021 (GSK3 inhibitor) treatment, β-catenin in both uninfected and infected cells cannot be phosphorylated nor degraded. Both cell types are more rigid due to the improve connection between actin filaments and cadherin which severely limits the size of the Pf schizont.

**Supplementary Figures**

**Figure S1:** **The impact of heat inactivated human sera on Pf schizont development.** PHH monolayers were cultured in either William’s B (WB) or William’s B supplemented with 10% heat inactivated sera (WBH) for six days p.p. and infected with either NF175 (A) or NF135 (B) or NF54 (C). Data are shown for respectively schizont number, size, total DAPI content, percentage of human glutamine synthetase (hGS) positive schizonts, total hGS level per schizont and total merozoite surface protein 1 (MSP1) level per schizont. As for the number of schizonts, each dot represents an independent experiment showing the mean of two replicates. As for the size and total DAPI content graphs, each dot represents an independent experiment showing the mean of the median of at least 100 schizonts per replicate (2 replicates) were measured (except in NF54 condition where there is not enough schizonts present). For the percentage of hGS positive schizonts, each dot represents an independent experiment where at least 100 schizonts were examined from one replicate. For the total hGS level and total MSP1 level, each dot represents an independent experiment of the median from at least 100 schizonts (except in NF54 condition where there is not enough schizonts present). The error bars show the mean with the standard deviation. The p-values are generated by performing an unpaired t-test between WBH and WB and can be found in the supplementary information excel sheet (*** = 0.0001).

**Figure S2:** **Effect of specific inhibitors of PHH gene-signaling pathways on the expression Pf schizont markers** A) Schematic experimental setup of Figure 3 and S3. PHHs were treated for 6 days p.p with either B27 or B27 supplemented IWP2/SB431542/Forskolin/DAPT/LDN193189. The experiment was analysed at day 5 p.i. with NF175 B) Total number of hepatocytes per well with each dot representing the average three wells of one biological replicate. C)Total hGS per schizont with each dot representing the median of at least 100 schizonts. The percentage of hGS (D) and MSP1 (E) positive schizonts: each dot is an independent experiment where at least 100 schizont is measured. The grey dotted line (per graphs C-E) shows the median measurements of schizonts grown in B27 (from four independent experiments) as each host pathway inhibitor treatment was made in media containing B27. The mean and standard deviation is shown for each graph. The p-values from a Dunnett’s multiple comparisons test are displayed. F) Representative confocal images (from four independent biological experiments) showing schizonts grown in B27 alone and B27 supplemented with IWP2, SB431542, DAPT, LDN193189, Forskolin stained with hGS, HSP70 and DAPI. Scale bar is 25 microns.

**Figure S3:** **The effect of B27 or B27 supplemented with 5C on *Pf-*permissiveness and schizont development in PHH.** The number of schizonts (A) and hepatocytes (B) on day 5 p.i. for NF135 and NF54 (n=1) at indicated time points p.p. when cultured in either B27 medium (grey) or B27 supplemented with 5C (blue). Each dot shows the mean of three replicates and the error bars show the standard deviation. C) Experimental setup of graphs D-L. PHHs were cultured for 6 days p.p. with either B27 or B27 with 5C and infected with NF175 parasites and analysed at day 5 p.i. D) Total number of hepatocytes per well with each dot representing the average three wells of one biological replicate. E)The number of schizonts per well was normalised to the number of schizonts counted in WBH (red dotted line). Each dot is the mean of an independent experiment with two replicates. For the schizont size (F) and total DAPI content per schizont (G), each dot represents the mean of an independent experiment of at least 100 schizonts. For the total hGS (H) and MSP1 (I) per schizont, each dot represents the median of an independent experiment of at least 100 schizont. For the percentage of hGS (J) and MSP1 (K) positive cells, each dot represents an independent experiment of at least 100 schizonts. D-K shows the mean with the standard deviation). Representative confocal images (from four independent experiments) showing schizonts grown in WBH or B27 alone or B27 supplemented with 5C on day 5 p.i. stained with hGS (L) or MSP1 (M), HSP70 and DAPI. Scale bar is 25 microns.

**Figure S4: The impact of the Wnt signaling pathway on numbers of developing schizonts.** A) The number of schizonts in NF135 infected PHHs treated with B27 or B27 supplemented with IWP2/CHIR99021. Each graph shows an independent experiment, each with duplicates per condition. B) Number of schizonts (left) and number of hepatocytes (right) in NF175-infected PHHs treated with B27 or B27 supplemented with IWP2/CHIR99021. Each graph shows the mean and the standard deviation of three independent experiments: each experiments have duplicate wells. There was no statistic difference between conditions within the same day post infection. Representative confocal images (from three independent biological experiments) showing NF135 (C) and NF175 (D) schizonts B27 or B27 supplemented with IWP2/CHIR99021 on day 5 p.i. stained with hGS, HSP70 and DAPI. Scale bar is 25 microns.

**Figure S5: The impact of Wnt signaling on determining NF175 development in the liver** A) schematic showing the media treatment timing and conditions. Hepatocytes are treated with B27 or B27 supplemented with IWP2/CHIR99021. After exposure to NF175, cultures were either kept in B27 or B27 supplemented with IWP2/CHIR99021 (closed circles) or returned to WBH (IWP2* and CHIR99021* - open circles). The number of schizonts (B), size of schizonts (C), total nuclear content per schizont (D) and total MSP1 per schizont (E) on day 5 post infection. The number of schizonts (F), size of schizonts (G), total nuclear content per schizont (H) and total MSP1 per schizont (I) on day 7 post infection. Each dot represents an independent biological donor where the median of at least 100 schizonts were measured. Dunnett’s multiple comparison test (to WBH) were performed for each graph, and the statistical significance is indicated: see supplemental information excel sheet for the full p values.

**Figure S6: IWP2 treatment modulates expression of target genes downstream of Wnt responsive transcription factors.** A) PCA plot of all replicates of freshly-isolated, “mock-cultured”, IWP2 or 5C-treated hepatocytes. B) Bar-plot depicting the scaled activity scores of key Wnt, TGFβ and non-canonical Wnt transcription factors across freshly-isolated hepatocytes, “mock-” and IWP2-treated hepatocytes. C-D) Volcano plots depicting a repressive effect of IWP2 treatment on the target genes underlying key Wnt signaling transcription factors CTNNB1 (β-catenin; C) and TCF7L2 (D). Genes that are positively modulated by these TFs and suppressed upon IWP2 treatment are highlighted in blue.

**Supplementary information on statistical tests**

Each tab of the excel sheet presents a table of the statistical tests performed per figure with the associated P-values.
