# Supplementary material for: Inhibition of Wnt signaling in primary human hepatocytes promotes Plasmodium falciparum liver stage development
Source: PLoS Pathog. 2025 Dec 22;21(12):e1013800. doi: 10.1371/journal.ppat.1013800 (PMC12795456; doi:10.1371/journal.ppat.1013800)
Supplement: Attachment — Submitted filename: Response to reviewer_20251210.docx [file ppat.1013800.s015.docx]

Reviewer 3:

This is the second revision of a paper that describes how inhibiting wnt signaling in primary human hepatocyte cultures can improve Plasmodium falciparum liver stage development. The primary outcome of the inhibition is clear and unequivocal. However, the paper is still poorly written and changes that reviewers have asked for on two separate occasions have still not been addressed. In many instances the writing lacks clarity and is opaque. The experimentation is sound but the manuscript needs work.

We thank the reviewer for his/her patience in helping us improve the manuscript. We have adopted the changes as requested and added extra explanation in unclear points for easier reading.

Reviewer 2:

The authors have toned down their statements to reflect more accurately the data and changed the title accordingly.
They bring interesting information in their rebuttal, which unfortunately was not included in the manuscript.
In particular, the observation that IWP2 increases schizont size when compared to (historical) fresh hepatocytes is important as it suggests a direct impact of Wnt inhibition on parasite development.
Also, I still believe that the discussion should include a paragraph to discuss parasite numbers (invasion) versus size (development), perhaps showing the entry factor expression data shown in the rebuttal as supplementary data. This is important as well as it shows that the invasion rate might still be a limiting factor when using aged hepatocyte cultures, even with Wnt inhibitors.

We have added two paragraphs (lines 306-314 and 339-349) to reflect the data presented in the rebuttal, as requested by the reviewer.
